# Mixed Signals? Morphological and Molecular Evidence Suggest a Color Polymorphism in Some Neotropical *Polythore* Damselflies

**DOI:** 10.1371/journal.pone.0125074

**Published:** 2015-04-29

**Authors:** Melissa Sánchez Herrera, William R. Kuhn, Maria Olalla Lorenzo-Carballa, Kathleen M. Harding, Nikole Ankrom, Thomas N. Sherratt, Joachim Hoffmann, Hans Van Gossum, Jessica L. Ware, Adolfo Cordero-Rivera, Christopher D. Beatty

**Affiliations:** 1 Department of Biological Sciences, Rutgers University, Newark, New Jersey, United States of America; 2 Grupo de Ecoloxía Evolutiva e da Conservación, Departamento de Ecoloxía e Bioloxía Animal, Universidade de Vigo, Galiza, Spain; 3 Department of Evolution, Ecology and Behaviour, Institute of Integrative Biology, University of Liverpool, Liverpool, United Kingdom; 4 Department of Biology, Santa Clara University, Santa Clara, California, United States of America; 5 Department of Biology, Carleton University, Ottawa, Ontario, Canada; 6 ALAUDA—Arbeitsgemeinschaft für landschaftsökologische Untersuchungen und Datenanalysen, Hamburg, Germany; 7 Evolutionary Ecology Group, University of Antwerp, Antwerp, Belgium; Field Museum of Natural History, UNITED STATES

## Abstract

The study of color polymorphisms (CP) has provided profound insights into the maintenance of genetic variation in natural populations. We here offer the first evidence for an elaborate wing polymorphism in the Neotropical damselfly genus *Polythore*, which consists of 21 described species, distributed along the eastern slopes of the Andes in South America. These damselflies display highly complex wing colors and patterning, incorporating black, white, yellow, and orange in multiple wing bands. Wing colors, along with some components of the male genitalia, have been the primary characters used in species description; few other morphological traits vary within the group, and so there are few useful diagnostic characters. Previous research has indicated the possibility of a cryptic species existing in *P*. *procera* in Colombia, despite there being no significant differences in wing color and pattern between the populations of the two putative species. Here we analyze the complexity and diversity of wing color patterns of individuals from five described *Polythore* species in the Central Amazon Basin of Peru using a novel suite of morphological analyses to quantify wing color and pattern: geometric morphometrics, chromaticity analysis, and Gabor wavelet transformation. We then test whether these color patterns are good predictors of species by recovering the phylogenetic relationships among the 5 species using the barcode gene (COI). Our results suggest that, while highly distinct and discrete wing patterns exist in *Polythore*, these “wingforms” do not represent monophyletic clades in the recovered topology. The wingforms identified as *P*. *victoria* and *P*. *ornata* are both involved in a polymorphism with *P*. *neopicta*; also, cryptic speciation may have taking place among individuals with the *P*. *victoria* wingform. Only *P*. *aurora* and *P*. *spateri* represent monophyletic species with a single wingform in our molecular phylogeny. We discuss the implications of this polymorphism, and the potential evolutionary mechanisms that could maintain it.

## Introduction

A polymorphism occurs when genetic diversity produces discrete variation in a phenotypic trait among individuals within a species. Ford [1] defined polymorphism as “the presence of two or more discontinuous forms of a species in such proportions that the rarest of them cannot be maintained merely by recurrent mutation”. Studies of the evolution of color polymorphism (CP, hereafter) in a number of model systems (*Biston betularia* moths [2], *Cepaea* snails [3,4], ‘Happy Face’ spiders [5], and side-blotched lizards [6,7]) have been highly productive, increasing our understanding of the selective forces that maintain these polymorphisms. Recent research has shown that there are several mechanisms that contribute to the maintenance of CPs in natural populations, and that also contribute to the speciation process [8]; in many systems polymorphisms are maintained by natural selection in the form of predation, but sexual selection in the form of mate choice is also common. Despite this research, our knowledge of how genetic diversity is maintained in nature and its relation with CP is still poorly understood. The exploration of novel polymorphic systems can add new insights and fill some of these gaps. We here present our initial findings on wing color diversity in the Neotropical damselfly genus *Polythore* (Zygoptera: Calopterygoidea: Polythoridae), which appears to maintain an elaborate polymorphism in wing color.

Dragonflies and damselflies (Insecta: Odonata) are amongst the most conspicuous of insects, often due to their wing coloration [9]. These colors are generated by a variety of mechanisms, such as pigments, Tyndall scattering, and optical interference [9–11]. Some of the most intense wing color employed by damselflies (Zygoptera) is produced through wing pigmentation. A number of species, mainly in the superfamily Calopterygoidea, possess wing coloration generated by sequestration of either carotenoid (red and orange) pigments or melanin (black); waxy pruinosity on the wings may also generate white and/or ultraviolet colors [12,13]. The intensity, uniformity and spatial extent of wing coloration in males typically indicates mate quality and impacts male fitness (i.e. mating success) [14–19]. These damselflies thus present an extremely productive model system for the study of the evolution of sexually selected traits [20].

In most odonate species, wing color patterns are fairly simple; interspecific differences are usually based on the single color employed and/or the relative size and shape of the color patch. Among species in the genus *Calopteryx*, for example, small differences in simple wing patterns appear to allow for effective conspecific recognition during mating; while mating structures are not significantly differentiated between species, hybridization events appear to be relatively rare, even in places where different species live syntopically [21–27].

**Fig 1:**
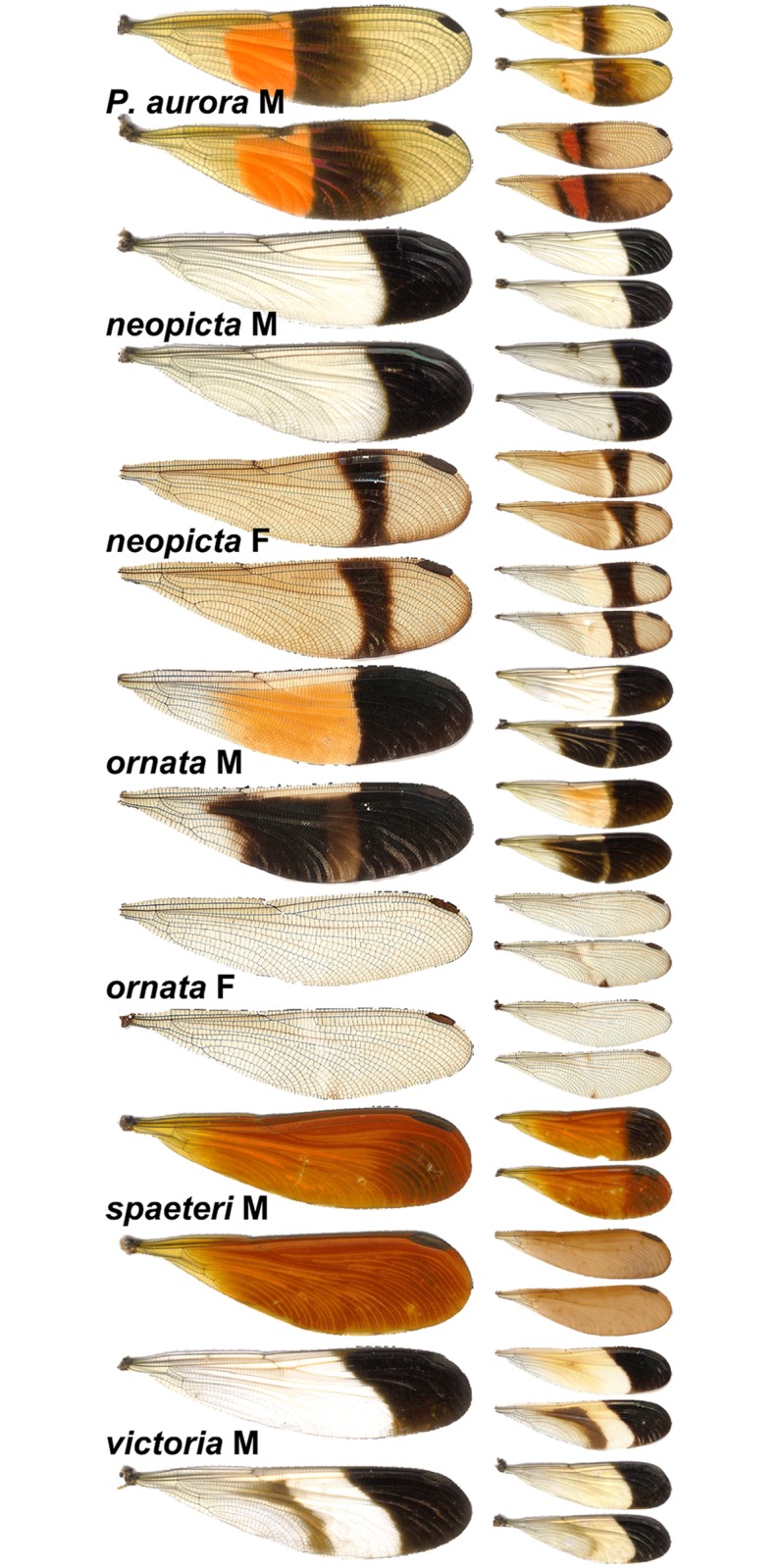
Examples of color polymorphism in *Polythore* taxa used in this study. For each wingform, the typical form is shown on the left, and extrema are shown on the right.

The genus *Polythore* currently contains 21 described species, distributed primarily along the eastern slopes of the Andes in South America [28,29]. *Polythore* typically dwell in small stream environments within healthy rainforests; larvae live in the streams and adults generally remain close to their stream habitat [28,30]. In most calopyterygid species, either red/orange pigments or black pigments are used, but not both [12,13,31]. *Polythore*, however, is the rare exception: many “wingforms” use at least two colors to generate combinations of black, white, yellow, and orange, displayed in bands and other geometric patterns that differ dramatically between described species (Fig 1). Their vibrant wing color pattern is the primary trait used to describe the species in this genus [28,32]. There is, in fact, a surprising lack of variability in other morphological traits: structures such as the male cerci, which function as a “lock and key” mechanism during copula in many other species [33] show little or no variation in *Polythore*, even over wide geographic distances. Why selection has brought about such elaborate wing patterning in *Polythore* is thus of interest, and a key first step to addressing this question would be the quantitative assessment of these patterns. Recent work by Sánchez Herrera et al. compared the variability in wing patterns of *P*. *procera* from Colombia, and analyzed genetic diversity in several populations; morphological characters (including wing color patterning and the structure of male accessory genitalia) were not significantly different among populations, but genetic diversity among certain populations was quite high, suggesting the presence of at least one cryptic species[34].

Here we quantify the wing color patterns of individuals from five *Polythore* species (*P*. *aurora*, *P*. *neopicta*, *P*. *ornata*, *P*. *spaeteri*, and *P*. *victoria*). The first of these species has a relatively wide distribution in the Amazon Basin in Peru, Ecuador and Brazil; the remaining species have limited distributions in the Amazon Basin of Central Peru on the eastern slopes of the Andes. As the wing patterns of these species are very elaborate, quantification is not a simple task. We here utilized three methodologies: geometric morphometrics, chromaticity analysis, and Gabor Wavelet Transformation (GWT). These methods allow us to measure differences in wing color pattern, and to determine which components of the wing pattern contribute to those differences. To begin exploring phylogenetic relationships, we also sequence the mitochondrial barcode gene Cytochrome oxidase I (COI) to assess species definitions and population genetic diversity. Finally, we use the results of our morphometric analysis in a novel approach, to generate a morphological dataset for phylogenetic analysis, and directly compare the resulting tree with our molecular phylogeny. The use of wing color and pattern to describe species is based on one hypothesis about the congruence between morphological and genetic diversity in these damselflies. First, coloration may have been selected through sexual selection to indicate mate quality or mate identity. If this were the case, we would predict that there would be high congruence between wing color diversity and phylogenetic patterns. If, however, speciation is currently taking place or there is introgression between individuals with different wing forms, we would not expect congruence in morphological and molecular data.

We found that, while wing pattern diversity is high, distinct and discrete wingforms exist. However, these distinct wingforms do not match clearly with individual species as determined through preliminary phylogenetic analysis: significant differences exist in the genetic diversity of individuals with the same wingforms, while in other cases, there are no differences in wingforms between individuals that are different genetically. We consider the implications of these results regarding the true diversity within this genus, and the potential explanations for why this diversity may have evolved.

## Methods

### Taxon Sampling

**Fig 2:**
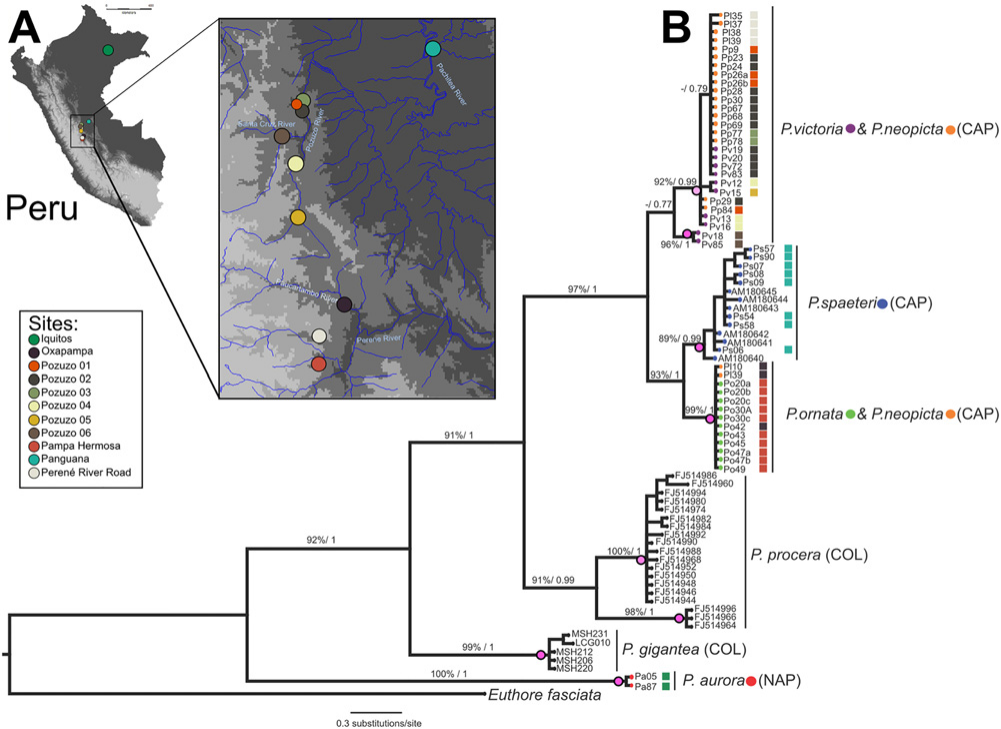
Collection localities of *Polythore* used in this study and phylogenetic reconstruction using Cytochrome oxidase I (COI). (A) map of collection localities of and (B) phylogenetic reconstruction (best ML phylogram) using COI data, values above the branches represent bootstraps support (ML) and posterior probabilities (BI). The magenta dots on the nodes represent the OTUs or species boundaries estimated by the PTP species delimitation model; lighter color represents less-supported probability for that OTU.

A total of 94 specimens were collected from localities in regions of the lower and central Amazon basin of Peru in July and September of 2008 (Fig 2A, Table A in S1 File). Specimens were collected with permission from the Instituto Nacional de Recursos Naturales (INRENA) of Peru (Authorization #62-2008-INRENA-IFFS-DCB and #016 C/C-2008-INRENA-IANP). All *Polythore* specimens used for these analyses were collected on private lands with permission from the landowners. No protected species were sampled. After collection, specimens were either placed in individual glassine envelopes that were stored in airtight dry containers, or in vials with absolute ethanol. Both dried and ethanol-preserved specimens of males and females were used for wing coloration and DNA analysis.

### Color Polymorphism Quantification

In order to measure morphological differences in the wings among *Polythore* species and among males and females within those species, and to facilitate quantitative comparison of morphological and molecular evidence, we analyzed images of wings using three different methods. First, shape analysis (geometric morphometrics) was used to compare the shape and relative position of bands in the wings (see “Landmarking analysis” below). Second, differences in the color of different parts of the wings were captured using a novel method for color analysis (see “Chromaticity analysis” below). Finally, wing patterning (the arrangement of light and dark patches in the wings) was compared using a technique that is common in the field of computer vision (see “Gabor wavelet transformation (GWT) analysis” below). These approaches allowed us to numerically describe three different, but interconnected aspects of wing morphology (shape, color, and patterning) in the highly polymorphic *Polythore*.

#### Imaging

Four males of *P*. *aurora*, 21 males and 5 females of *P*. *neopicta*, 17 males and 6 females of *P*. *ornata*, 11 males of *P*. *spaeteri*, and 16 males of *P*. *victoria* were digitized for morphological analysis. The left forewing and hindwing of each specimen was excised and scanned using a HP Deskjet F2180 scanner/printer (Hewlett-Packard Co., Palo Alto, CA, USA) in color (RGB) at a resolution of 600 dpi. The wings were placed so that the ventral side of the wing was scanned (the side of the wing most often in view at rest and most commonly in view from the ground during flight). Images were saved as JPEG files.

#### Computation

Images were processed and analyzed (except where indicated) using custom scripts written in *Mathematica* (v10; [35]). These scripts are available for download as a *Mathematica* notebook (S2 File).

#### Landmarking analysis

**Fig 3:**
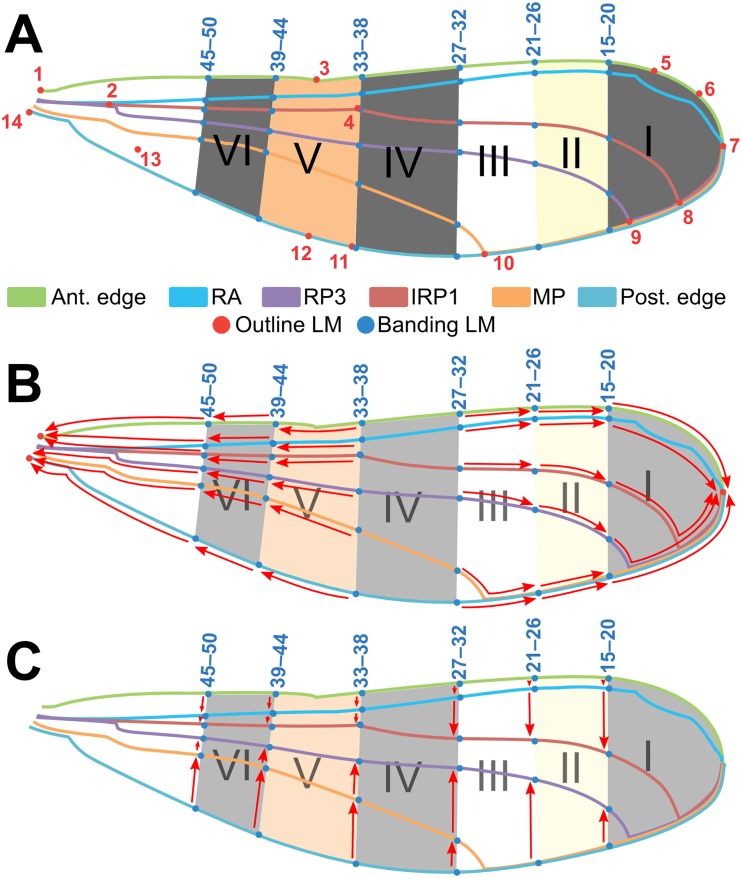
“Default wing” used as a template for landmarking analysis of fore- and hindwings of *Polythore* wingform. The positions of six major bands (I–VI) are marked as the cross six longitudinal veins: anterior edge or costa (C), radius anterior (RA), second branch of radius posterior (RP2), third branch of radius posterior (RP3), media posterior (MP), and posterior edge (venation terminology per Riek & Kukalová-Peck (1984)). (A) LMs 1–14 are major morphological points representing the basic venation pattern (~outline) of the wing, and LMs 15–50 represent the proximal edges of the six bands. (C–D) red arrows depict protocol for “collapsing” LMs in (C) proximodistal and (D) anteroposterior directions in wings where bands are missing or do not extend the full width of the wing (see Methods text for full description).

To compare relative position and shape of wing banding patterns, allowing for comparison among individuals, landmarks (LMs, hereafter; i.e. Cartesian coordinates) were placed on the wing scans. A standard set of 50 LMs (Fig 3A) was placed on each digitized fore- and hindwing using tpsDig (v2.05; [36]); all landmarking was performed by a single technician to ensure consistent LM placement.

Two subsets of landmarks were applied to each wing. LMs 1–14 designated the position of junctions of six major longitudinal veins in the wing, reflecting underlying wing structure (Fig 3A, red dots). LMs 15–50 were positioned to designate the outlines of color bands on the surface of the wing (Fig 3A, blue dots). A number of different bands exist in the wings of *Polythore* species; to account for all possible banding patterns we designed a “default wing” (Fig 3) with six total bands (numbered I–VI) as a template for placement of LMs, such that there were sufficient LMs to capture the full range of wing patterning for all species under study. See Fig. A in S1 File for examples of wing scans and their respective landmarks.

LMs were placed exactly where the band intercepted with six reference veins (the anterior edge, RA, RP3, IRP1, MP, and the posterior edge, using venation terminology from Riek & Kukalová-Peck [37]), so that there were six LMs per band. In some cases, a specimen lacked one or more bands and/or had bands that did not extend all the way to the anteroposterior or proximodistal edges of the wing. In order to account for this, LMs were “collapsed” to other LMs using a standardized protocol as described below.

The default wing was divided into two parts between bands III and IV (Fig 3B). If the specimen lacked any of the bands I–III, the points for that band were collapsed to the next existing band to the right (distally), or to the outermost point (LM 7) if the specimen lacked band I. Likewise, if the specimen lacked any of the bands IV–VI, the LMs for those bands were collapsed to the next existing band to the left (apically). If band VI was absent, its LMs were collapsed equally between LMs 1 and 14, with the upper three LMs being collapsed to LM 1 and the lower three being collapsed to LM 14. When a band did not extend all the way to the anterior or posterior edge of the wing, it was collapsed medially to the point where the next closest reference vein intersected the band (Fig 3C).

To remove the effects of scale and rotation, LMs for the full set of fore- and hindwings were Procrustes superimposed [38], separately, and recombined into one dataset for analysis. Procrustes superimposition was done using the Geometric Morphometrics package for *Mathematica* (v11.1; [39]). The landmarking procedure described here resulted in 200 coefficients per specimen (50 two-dimensional Cartesian coordinates per fore- and hindwing).

#### Image squaring

As preprocessing for the remaining morphological analyses, wing scans were standardized for comparison, using an automated script. Images were converted into a 512-px square with a black background, in which the wings were masked, rotated so that their upper margins (costa vein) were horizontal, and then rescaled to a length of 512 px, and where the fore- and hindwings are placed at the top of the upper- and lower halves of the square, respectively (Fig. B in S1 File). The square shape was chosen because it is required for the Gabor wavelet transformation (GWT), while the 512-px dimensions of the square were chosen as a compromise between image detail and computation required for image analysis. Manual masking was required for one *P*. *victoria* male (pv18), for which scan the automatic preprocessing script failed. S2 File provides a more detailed explanation of the image processing procedure, along with the script.

#### Chromaticity analysis

To compare wing coloration across specimens, square RGB images were separated into their component image channels (red, green, and blue), where each channel is an array of pixel values ranging from 0 (black) to 1 (white). Pixel values were transformed into chromaticity coordinates (CCs), which removes the possible effect of non-standardized lighting between images (luminance) and the correlation between red, green, and blue channels [40–42]. CCs (*r*, *g*, and *b*) are calculated with a simple transformation *r = R/*(*R + G + B*), *g = G/*(*R + G + B*), and *b = B/*(*R + G + B*), where *R*, *G*, and *B* are the pixel values for the red, green, and blue channel, respectively [42].

Instead of comparing every transformed pixel value among the images of every specimen, which would be computationally taxing and potentially uninformative, the square images were broken into successively smaller sub-images: the first comprising the entire image, the next two comprising each wing, separately, then the proximal, middle, and distal thirds of each wing, and finally the upper and lower halves of each of those wing thirds (see Fig. C in S1 File for visual explanation). This image-sampling procedure is somewhat similar to that of the GWT (see below; [43]). The mean pixel values for *r*, *g*, and *b* were then calculated for each sub-image. This process yielded 63 chromaticity coefficients per image (21 sub-images × 3 CCs per sub-image).

#### Gabor wavelet transformation (GWT) analysis

A Gabor wavelet is a type of scalable, rotatable, two-dimensional wave form, which can be used to distill the complex features of an image into relatively few coefficients that describe the gross patterning of light and dark regions in the image (e.g. wing veins, edges, and color patches). Gabor wavelet transforms (GWTs), where a set of Gabor wavelets are convolved over images to encode them, have many applications for image processing, including optical character recognition, fingerprint and iris recognition, and image compression [44,45] since Gabor wavelets behave similarly to cells of the visual cortex in mammals [46].

In this study, GWTs were used to transform each of the square images into a list of coefficients, describing the patterning of its wings. Each RGB image was first converted to grayscale, then the two components of the wavelet (real and imaginary) were applied using four scales (512, 256, 128, and 64 px in diameter; see Fig. D in S1 File for wavelet arrangement) and three rotations (0°, 120°, 240°), giving 510 coefficients per image. The script for this analysis was adapted from code provided by G.J. Russell.

#### Comparative analysis of wing morphology

Wingform morphology was compared using the landmarking, chromaticity and GWT datasets individually, as well as a combined set of all three. Each dataset was transformed using Discriminant Analysis of Principal Components (DAPC)[47]. This two-step analysis comprised (1) finding the first 50 principal component axes (PCs, in *Mathematica*), and (2) finding the Linear Discriminants (LDs) of those PCs in PAST [48]. To determine which coefficients from the untransformed dataset contributed most to each DAPC axis (i.e. the separation between wingforms), the contributions of each coefficient were calculated (similarly to [47]) as DAPC contribution vectors = |(loading vectors for 1^st^ 50 PCs) (LD loading vectors)|. Values in each contribution vector were converted to proportions of the sum of values in that vector, so that each value in a contribution vector gave the relative contribution of the corresponding dataset coefficient to that DAPC axis (i.e. for a given contribution vector, coefficients with the highest values contributed the most to the separation of wingforms along the corresponding DAPC axis). For the seven wingforms analyzed in this paper, the DAPC process produced 6 (7 groups—1) DAPC axes, and therefore, 6 DAPC contribution vectors. Additionally, Mahalanobis distances [49] were calculated between wingform centroids, using all 6 DAPC axes, to compare how well each analysis separated wingforms.

### Phylogenetic Reconstruction

#### DNA amplification and sequencing

Genomic DNA was extracted from thoracic muscle using either a CTAB protocol (modified from Doyle & Doyle [50]) or the NucleoSpin Tissue kit (Macherey-Nagel, Düren, Germany), following the manufacturer’s instructions, except that we incubated the sample at 50°C for 24 h and used 50 μL to elute the DNA.

Nucleotide variation was assessed in one mitochondrial gene (Cytochrome oxidase I, COI), using the universal primers CJ-2195 and TL2-N-3014 [51]. PCR was carried out in 20 μL volume reactions containing 1 to 2 μL of DNA, 1X Buffer, 2 mM MgCl_2_, 0.8 mM dNTPs, 0.5 mM of each primer, and 0.03 U/μL of KapaTaq DNA polymerase. PCR conditions were 94°C for 60 s (two cycles), followed by 94°C for 45 s, 48°C for 45 s and 72°C for 60 s, and 29 cycles at 94°C for 45 s, 52°C for 45 s and 72°C for 1 min and 30 s. PCR products were purified using the NucleoSpin Gel and PCR purification kit (Macherey-Nagel, Düren, Germany). Sanger Sequencing reactions were performed bidirectionally at MACROGEN Inc. laboratories (Seoul, Korea). Forward and reverse sequence strands were assembled and edited using SeqManII v5.03 (DNAstar, Inc., Madison, WI, USA) and consensus sequences were aligned using Clustal W [52], as implemented in MEGA v. 5.1 [53].

#### Molecular phylogenetics

In order to increase our overall sample size and improve the resolution of our phylogenetic analyses, we included the individuals used for wing morphometric analysis as well as sequences from an additional 31 individuals from the following species: *P*. *procera* (19 individuals), *P*. *spaeteri* (6 individuals), *P*. *gigantea* (5 individuals) and the confamilial *Euthore fasciata* which was used as outgroup (one individual). The sequences associated with these additional individuals were downloaded from GenBank (see Table A in S1 File for accession numbers for all sequence data). Phylogenetic reconstructions were performed using maximum likelihood (ML) and Bayesian inference (BI) criteria. ML analysis was implemented in Garli (v2.0; [54]). The best nucleotide substitution model for our data was HKY+G, as selected by Mega (v5.2; [53]). Bootstrap supports for each branch were obtained after running 5000 pseudo-replicates of the best estimated topology; the consensus tree was summarized using SumTrees (v3.3; [55]). The BI analysis was performed in MrBayes (v3.2; [56]), where two independent runs were conducted. Four different heated MCMC chains were used; we ran 10 million generations, sampling a topology every 100 cycles, using default priors for all parameters, and using the GTR+I+G substitution model previously selected by jModeltest 2 [57,58]. Convergence in the posterior probabilities for the two runs was assessed by examining the average standard deviation of split frequencies, and using Tracer v1.6 [59]. Burn-in samples (1 million generations) were discarded and the remaining samples were combined to produce a 50% majority-rule consensus tree, with bipartition frequencies equal to posterior probability values. Both topologies (i.e. ML and BI) were visualized using FigTree (v1.4.1; [60]). We estimated the evolutionary net divergence between the obtained clades and the standard error using Mega [53].

#### Morphological phylogenetics

For comparison with the molecular topologies, we also performed a morphological phylogenetic reconstruction using parsimony. Coefficients from the landmarking, chromaticity, and GWT analyses were combined into one character matrix of continuous characters. Characters that contained only zeros were removed from the matrix, then each character was standardized so that its standard deviation and mean were 1 and 0, respectively, rescaled so that its values were between 0 and 1, and rounded to three decimal places for analysis. Only male Peruvian specimens were used for this analysis to allow for identification of relationships between individuals from this single region, and to allow for a single wingform (that of the male) to be potentially associated with the molecular species identification. Phylogenetic reconstructions were performed on these morphological data using TNT (v1.1; [61]). Trees were reconstructed using the ‘traditional search algorithm’ in TNT with 1000 replicates of Wagner trees set as the starting trees and subtree-pruning-regrafting (SPR) as the swapping algorithm. Consistency and retention indices were calculated for the best topology. Bootstrapping was also performed in TNT using the same conditions as above with 500 replicates. Resultant topologies were exported as Nexus files, processed in Mesquite v2.75 [62], and visualized in FigTree [60].

#### Population genetics analysis

In order to assess the intraspecific genetic variation that the traditional phylogenetic tree analyses lack the power to solve [63], we applied a network representation of the haplotype relationships, including unsampled haplotype variants. Thus we calculated a Minimum Spanning Network (MSN) in the population genetic suite Arlequin (v3.5, [64]), and visualized it using HapStar [65]. To establish the genetic diversity of the geographical populations sampled, polymorphism statistics (i.e. number of haplotypes (h), haplotype diversity (Hd), genetic diversity (π), and genetic diversity per segregating sites (*θ*(*S*)) were estimated for each geographical population where the total number of individuals was > 10 (e.g. Pozuzo, Pampa Hermosa, and Panguana) with DnaSP (v5.10; [66]). Neutral evolution was tested with the Tajima D test [67] in Arlequin, assuming the well-accepted premise that mtDNA does not recombine. To determine the degree of population structure between all the geographical populations, pairwise F_ST_ values were calculated in Arlequin and 95% statistical significance for each test was obtained by 10,000 randomizations.

#### Species delimitation

To test if the morphospecies described using color patterns are consistent with our phylogenetic hypothesis; we ran a single-marker model for species delimitation called a Poisson Tree Processes (PTP). This model relies on the number of substitutions of a tree topology, assuming that the number of substitutions among species will be higher than the number of substitutions within the species[68]. The ML phylogram was used to calculate the probabilities that support species boundaries detected by the model. PTP was run via the web interface sponsored by the Exelixis Lab (Alexis Stamatakis: http://sco.h-its.org/exelixis/web/software/PTP/index.html).

## Results

### Color Polymorphism Quantification

In total, the wings of 79 *Polythore* specimens from the five species collected from Peru were analyzed with the three color polymorphism (CP) quantification methods (landmarking, chromaticity, and GWT). These included both males and females of *P*. *neopicta* and *P*. *ornata*, and males only for *P*. *aurora*, *P*. *spaeteri*, and *P*. *victoria*—seven wingforms in total. Upon visual comparison, patterning varied markedly both within and among wingforms (Fig 1). *P*. *aurora* males appeared to be the most variable, while *P*. *neopicta* males and *P*. *ornata* females appeared to be the least variable.

#### Landmarking analysis

**Fig 4:**
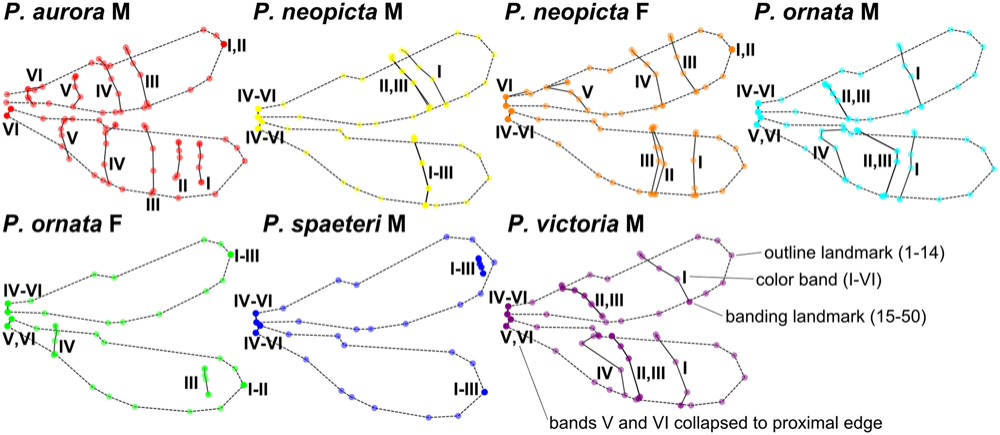
Mean wing shapes for *Polythore* wing morphs from landmark analysis. Fifty landmarks were taken from individuals of the 7 wing morphs, Procrustes superimposed, and averaged together for each wing morph. Outline landmarks (LMs 1–14) are dashed; color bands (LMs 15–50) are solid lines and numbered from I to VI. See Fig 2 and text for detailed description of landmarking protocol. M = male, and F = female.

Wings in all scans were landmarked; examples of specimens with landmarks are shown in Fig. A in S1 File. Mean shapes for the wingforms are shown in Fig 4. In all wingforms, at least one band was collapsed to the proximal or distal end of the wing, while all six bands were collapsed in the forewing of *P*. *ornata* females and hindwing of *P*. *spaeteri* males (see Fig 1).

#### Comparison of morphological analyses

**Fig 5:**
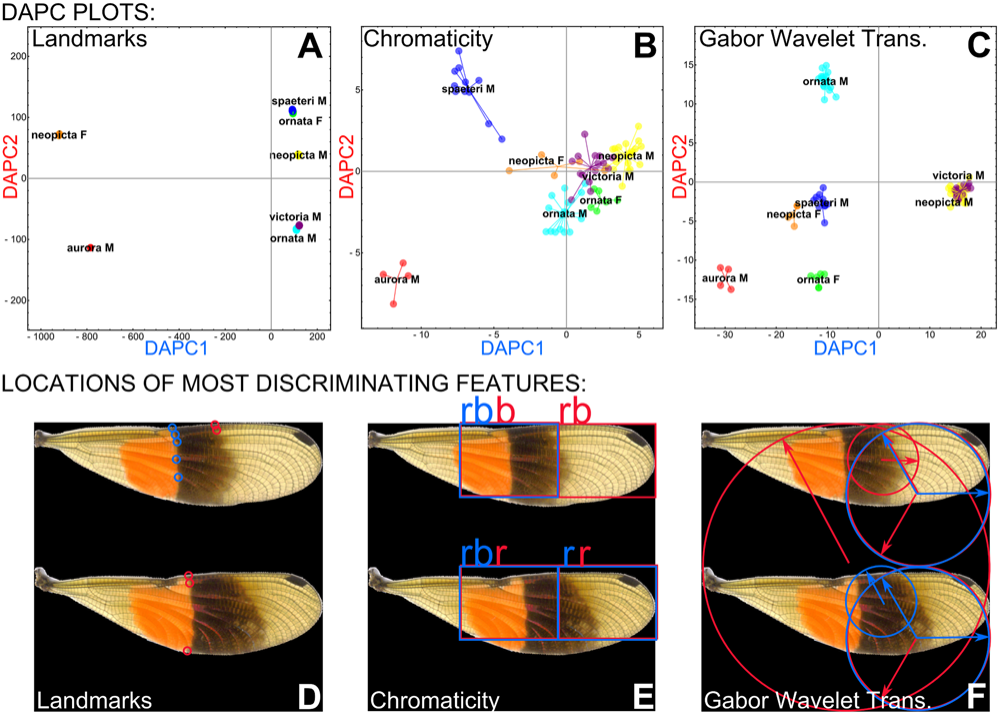
Discriminant analysis of morphological results. Discriminant analysis of principal components (DAPC) plots for (A) landmarking, (B) chromaticity, and (C) GWT analyses. (D–F) areas of the wings corresponding to the 5 most discriminating coefficients for DAPC axes 1 and 2 for each of the analyses (see Results text for further explanation of D–F). Note: here, these locations are superimposed onto an image of a *P*. *aurora* male, for presentation, but represent variation among all individuals/wingsforms.

DAPC and Mahalanobis distances (MDs) were used to determine which morphological analysis produced the best separation between wingforms. Plots comparing the first two DAPC axes (i.e. the first two dimensions of the 6-dimensional discriminant space) are shown in Fig 5A–5C and Fig. F.A in S1 File for the landmarking, chromaticity, GWT, and combined analyses, respectively. From these DAPC plots, the combined analyses and landmarking analysis appeared to give the most separation, while the chromaticity analysis appears to give the least separation. Interestingly, different wingforms group together depending upon the method used. For instance, *P*. *victoria* males grouped with *P*. *neopicta* males in the chromaticity, GWT, and combined analyses, but grouped with *P*. *ornata* males in the landmarking analysis. Similarly, *P*. *ornata* female and *P*. *spaeteri* males were very similar according to the landmarking and combined analyses, but very different according to chromaticity and GWT. *P*. *aurora* males were consistently separated from all other groups. Despite these apparent differences, however, the MD for pairs of wingforms (measured in standard deviations), calculated from all six DAPC axes, showed similar degrees of separation between most wingforms (Table 1). Mean MDs were as follows: landmarking, 4.32, combined, 4.31, GWT, 4.27, and chromaticity, 3.67. MD values consistently exceeded an arbitrary threshold of 3.0 for all wingform pairs except for *P*. *neopicta* male—*P*. *ornata* male and *P*. *neopicta* male—*P*. *victoria* male, which consistently showed lower MDs, and *P*. *ornata* male—*P*. *victoria* male and *P*. *spaeteri* male—*P*. *victoria* male, which had lower MDs from the chromaticity analysis. *P*. *aurora* males showed the highest degree is separation from all other wingforms along with *P*. *neopicta* female—*P*. *ornata* female.

**Table 1:** Mahalanobis distances (MDs; calculated from first 6 DAPCs) between wingforms using for different combinations of morphological coefficients.

Wingform Pair	LM	CHRM	GWT	ALL	MEAN
auroraM—neopictaF	5.92	4.92	5.8	5.88	5.63
auroraM—neopictaM	4.82	4.58	4.74	4.8	4.74
auroraM—ornataF	5.66	5.17	5.58	5.65	5.52
auroraM—ornataM	4.9	4.57	4.83	4.89	4.80
auroraM—spaeteriM	5.14	4.82	5.03	5.12	5.03
auroraM—victoriaM	4.96	4.58	4.88	4.93	4.84
neopictaF—neopictaM	4.39	3.18	4.32	4.36	4.06
neopictaF—ornataF	5.3	4.12	5.27	5.32	5.00
neopictaF—ornataM	4.49	3.35	4.43	4.47	4.19
neopictaF—spaeteriM	4.75	3.64	4.68	4.74	4.45
neopictaF—victoriaM	4.56	3.19	4.5	4.54	4.20
neopictaM—ornataF	4.02	3.49	4.02	4.05	3.90
neopictaM—ornataM	2.88	2.7	2.85	2.87	2.83
neopictaM—spaeteriM	3.27	3.13	3.23	3.25	3.22
neopictaM—victoriaM	2.98	2.09	2.87	2.93	2.72
ornataF—ornataM	4.14	3.64	4.15	4.15	4.02
ornataF—spaeteriM	4.36	4.08	4.4	4.41	4.31
ornataF—victoriaM	4.21	3.46	4.2	4.22	4.02
ornataM—spaeteriM	3.4	3.22	3.35	3.39	3.34
ornataM—victoriaM	3.11	2.25	3.08	3.1	2.89
spaeteriM—victoriaM	3.49	2.91	3.41	3.48	3.32
**MEAN**	**4.32**	**3.67**	**4.27**	**4.31**	**4.14**

M = male, F = female, LM = landmark coefficients, CHRM = chromaticity coefficients, GWT = Gabor wavelet transformation coefficients, and ALL = combined LM, CHRM, & GWT. MD is measured in standard deviations.

The axes of the DAPC plots shown in Fig 5A–5C and Fig. F.A in S1 File each comprise a linear combination of the original coefficients that were analyzed. We can determine the relative contribution of each of the original coefficients to each DAPC axis (i.e. which coefficients contributed the most to an axis, and thus best discriminated between wingforms). Relative contributions for the first and second DAPC axes for each analysis are shown in Figs. E and F.B in S1 File; the five highest-contributing coefficients are highlighted in red. Each of the coefficients corresponds to a particular location on the wings of our specimens, which can be mapped back onto the wings to determine which wing parts are most informative for wingform discrimination. In Fig 5D–5F and Fig. F.C in S1 File, the parts of the wings corresponding to the highest-contributing coefficients for each analysis have been highlighted on an example specimen, where blue and red shapes represent the highest contributors to the first and second DAPC axes, respectively. In the figure, landmarks are represented as small circles; chromaticity sub-images are represented as boxes around the appropriate portion of the square image, and letters “r”, “g”, or “b” tell which chromaticity channel the sub-image corresponds to; and Gabor wavelets are represented as circles over the piece of the image to which they were applied, each with an arrow denoting the direction of wavelet rotation. The top coefficients for the combined analysis all correspond to Gabor wavelets. Notice that there is some overlap between coefficients that contribute the most to the first and second DAPC axis (represented as overlapping red and blue shapes). Overall, the medial section, and to a lesser extent the distal section, of the fore- and hindwings contributed the most to discrimination between the different wingforms. This particularly corresponds with wing bands IV and V (Fig 2A). The top contributing coefficients in the landmark analysis were *y*-components—the *y*-axis here corresponds to the proximodistal axis of the wings—of landmarks 33–38 in the forewing and 27–28 in the hindwing (Fig 5D); while in the chromaticity analysis, the most important features were all in the red and blue chromatic channels of the image, not in the green channel (Fig 5E).

### Phylogenetic Reconstructions

#### Molecular phylogenetics

The phylogenetic topologies recovered for both criteria, ML and BI, were consistent (Fig 2B). *P*.*aurora* forms a well-supported monophyletic lineage, and seems to be the sister clade to all of the other taxa, although neither of the analyses estimates a support value for this branch. The highly supported *P*. *gigantea* clade is sister to all the other taxa, followed by two highly supported reciprocal monophyletic clades: *P*. *procera* from Colombia and a clade that contains all of the individuals collected in the Central Amazon Basin of Peru (i.e. *P*. *ornata*, *P*. *spaeteri*, *P*. *neopicta* and *P*. *victoria*, Fig 2B). Within this Amazon Basin clade, a distinctive clade of the species, *P*. *ornata/P*. *spaeteri* encompasses two reciprocal monophyletic clades (Net divergence = 1.27%, SE = 0.004), while another equal clade composed of *P*. *neopicta* and *P*. *victoria* shows no distinction between these species, with their distinct wingforms (Net divergence = 0.052%, SE = 0.0028). Interestingly, *P*. *neopicta* shows a paraphyletic position across the Amazon Basin clade; some individuals sampled in Oxapampa are closely related to *P*. *ornata*, while other individuals sampled in Pozuzo localities and the Perené River Road region are close to, and in some cases even indistinguishable from, *P*. *victoria* (Fig 2A).

#### Morphological phylogenetics

For our phylogenetic analyses that include morphological traits (767 characters), we limited our dataset to males (68), such that each species would be represented by a single wingform. We obtained only one most parsimonious tree with a tree length of 4500.780 steps. The overall topology has a consistency index of 0.170, suggesting these traits are highly homoplasic. The retention index was 0.519, showing that, despite the homoplasic nature, the synapomorphic characters are informative. We recovered *P*. *aurora* as sister to all other species, however only two individuals of this species clustered as a monophyletic clade (Fig. G in S1 File). The remaining species were recovered as monophyletic, although *P*. *ornata* and *P*. *victoria* had low bootstrap branch supports (9 and 5%, respectively), and two individuals of *P*. *ornata* were recovered within *P*. *victoria*.

### Population Genetic Analyses

**Fig 6:**
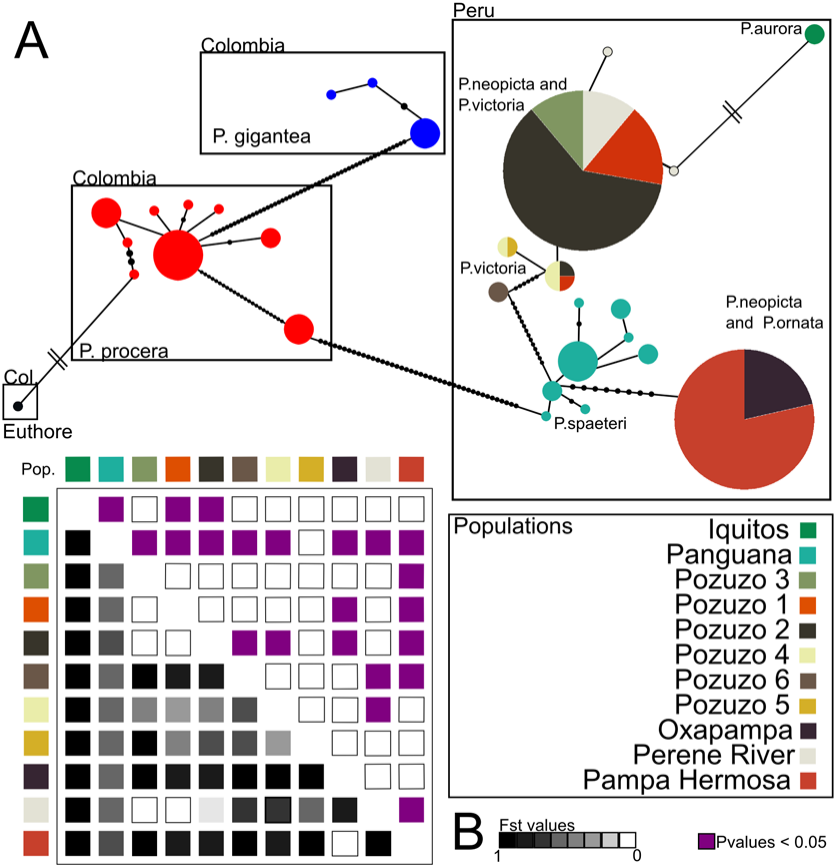
Comparison of mtDNA haplotypes among *Polythore*. (A) mtDNA haplotype network and (B) F_ST_ values across the geographical population. The COI haplotype network shows the relationships among 29 haplotypes. Each circle represent an haplotype, the size of the circles represents the number of individuals sharing the haplotype, colors represent the geographical population, black small circles show missing or unsampled haplotypes, branch lengths are fixed and represent the genetic distance between the haplotypes, parallels lines represent more than 100 missing haplotypes and high genetic distance between the haplotypes. The population matrix is organized from Northern to Southern geographic location, colors underneath the diagonal represent the F_ST_ values (i.e. gradient) and above the diagonal shows the significant p-values.

Overall, among the species and populations of *Polythore* included in this analysis we observed a total of 28 COI haplotypes. Of the two species sampled from Colombia, *P*. *procera* shows a higher number of haplotypes (h = 8) in comparison with *P*. *gigantea* (h = 3). Forty-one missing or unsampled haplotypes are estimated between *P*. *procera* and *P*. *spaeteri* from Panguana in Peru. The haplotype network suggests *P*. *spaeteri* (h = 8) as the source haplotypes from which all the remaining Peruvian *Polythore* haplotypes are derived (Fig 6). Eight unsampled haplotypes associate *P*. *ornata* from Pampa Hermosa, and *P*. *neopicta* from Oxapampa to *P*. *spaeteri*; while 12 unsampled haplotypes bridge *P*. *spaeteri* to *P*. *neopicta* and *P*. *victoria* from localities near Pozuzo and the Perené River Road. Finally, *P*. *neopicta* from the Perené River Road and *P*. *aurora* from Iquitos are joined by more than 100 missing haplotypes between them (Fig 6).

Genetic polymorphism statistics for the Peruvian populations with more than 10 individuals are shown in Table 2. The Panguana population (*P*. *spaeteri*) shows high haplotype and genetic diversity in comparison with Pampa Hermosa (*P*. *ornata*) and Pozuzo populations (*P*. *victoria—P*. *neopicta*). Pampa Hermosa (*P*. *ornata*) on the other hand shows a lack of genetic diversity, with only one haplotype representing the entire population. Tajima’s neutrality tests were all negative and non-significant (Table 2), which suggest that the COI sequence is not subject to selection and is evolving neutrally.

**Table 2:** Peruvian geographical population polymorphism statistics.

Population	N	h	Hd	π	θ(S)	Tajima’s D	Significance
Panguana	14	8	0.901	0.0027	0.00383	-1.06599	> 0.10
Pampa Hermosa	11	1	0	0	0	NA	NA
Pozuzo	24	4	0.536	0.0235	0.00326	-0.91249	> 0.10

Number of individuals (N), Number of haplotypes (h), haplotype diversity (Hd), genetic diversity (π), genetic diversity per segregating sites (θ(S)), and Tajima’s D test including significance.

The calculated fixation index values for population structure (F_ST_) show that the Panguana population (*P*. *spaeteri*) exhibited high and significant population structure (e.g. F_ST_ > 0.88) compared to all the other sampled geographic populations (Fig 6 and Table B in S1 File). The Pampa Hermosa population (*P*. *ornata*) showed high and significant population structure except with the Oxapampa population (*P*. *neopicta*) where the F_ST_ value is 0, suggesting possible gene flow or a shared ancestral polymorphism between these two populations. Among the six localities sampled near to Pozuzo (*P*. *victoria—P*. *neopicta*), we observed a degree of significant population structure across some of these populations (Fig 6 and Table B in S1 File). Pozuzo 1, 2 and 3 show high structure as compared to Pozuzo 4 and 6, suggesting restricted gene flow among these populations. The lack of structure among Pozuzo 1, 2 and 3 and Pozuzo 4, 5 and 6 suggests possible recent gene flow between these two sets of geographical populations. Interestingly the F_ST_ value of the Perené River Road population with Pozuzo 1, 2 and 3 is ~ 0, suggesting gene flow or a shared ancestral haplotype (Fig 6 and Table B in S1 File).

#### Species delimitation

The PTP species delimitation model supports 9 species or Operational Taxonomic Units (OTU’s) based on our single marker COI. One of the supported OTU’s was the outgroup *Euthore fasciata* (p = 1). Within the *Polythore*, some of the OTU’s were highly supported, with probabilities > 0.70, while others were supported with probabilities < 0.69 (Fig 2B). The Colombian species, *P*. *gigantea* and *P*. *procera*, were consistent with our expectations. *P*. *procera* was divided into two OTU’s (one supported with 0.658, and the other one with 0.903), which is consistent with the results previously obtained by Sánchez Herrera et al. 2010 (Fig 2B). *P*. *gigantea* was recovered as an OTU with a high probability (0.705). The Peruvian species show incongruence with the morphological expectations. *P*. *aurora*, *P*. *spaeteri* and *P*. *ornata/neopicta* were highly supported OTU’s, with probabilities of 0.988, 0.759 and 0.731, respectively (Fig 2B). The *P*. *victoria*/*P*. *neopicta* clade was supported with a low probability (0.474) as only one OTU (Fig 2B). Despite the latter, two individuals of *P*. *victoria* were considered as another OTU with a high of probability 0.701 (Fig 2B).

## Discussion

### Patterns of Diversity

Our analyses of wing color patterns in *Polythore* demonstrate that both the complexity of wing patterns—with some wingforms incorporating particular bands while others do not—and the interchanging of colors within those bands, come together to create a diverse range of phenotypes. Through our landmark analyses we show the influence of the different combination of bands on these phenotypes; for example, males of *P*. *victoria* and *P*. *ornata* are shown to be relatively similar to one another (Fig 5A and Table 1), despite their obvious color differences (Fig 1). In this case, both wingforms have very similar banding, but the colors of the bands differ. These striking differences in color are shown in our chromaticity results (Fig 5B); while there is less resolution between the different wingforms, overall levels of melanization, in both the medial and distal parts of the wing, drive differences. Our GWT results incorporate both of these components; here *P*. *victoria* and *P*. *ornata* are shown to be quite different (as they are on visual inspection). Males of *P*. *victoria* and *P*. *neopicta* show great similarity, sharing similar overall color patterns as they do, but differing in the presence (*P*. *victoria*) or absence (*P*. *neopicta*) of a single, medial band. There are also differences in the overall variability of the phenotype components—banding patterns are relatively invariant, as reflected through the tight clustering of individuals sharing a common wingform in the LM and GWT analyses, but we see greater differences in the chromaticity values within wingforms. It is of note that these differences among and within wingforms are driven most by the red and blue components of color; the green component did not contribute significantly. The combination of these analyses (Table 1 and Fig. F in S1 File) resolves all of the analyzed wingforms effectively, except for the male wingforms of *P*. *neopicta* versus *P*. *ornata*, and *P*. *neopicta* versus *P*. *victoria*; these wings all have black distal bands and differ only in the banding pattern preceding that shared band.

An interesting observation that can be made from these analyses concerns the way in which wing pattern diversity is generated in this group. While there are a number of distinct wingforms observed in *Polythore*, these do not represent unlimited complexity; as shown by the assembly of our ‘default wing’ for landmark analysis, there are a fixed number of repeated pattern elements within the wings, such that a particular band may be present or absent within a wingform, and if present may be of a different color within a different wingform. This suggests a fixed number of wing elements that can be expressed or suppressed, similar to the pattern elements observed in *Heliconius* butterflies [69,70]. In *Heliconius*, these pattern element shifts are made through a small number of allele differences, and the same may be true of *Polythore*. Exploration of these wing pattern elements within this highly diverse group will allow for an understanding of the mechanisms of wing pattern expression in the genus, and possibly more generally in odonates.

**Fig 7:**
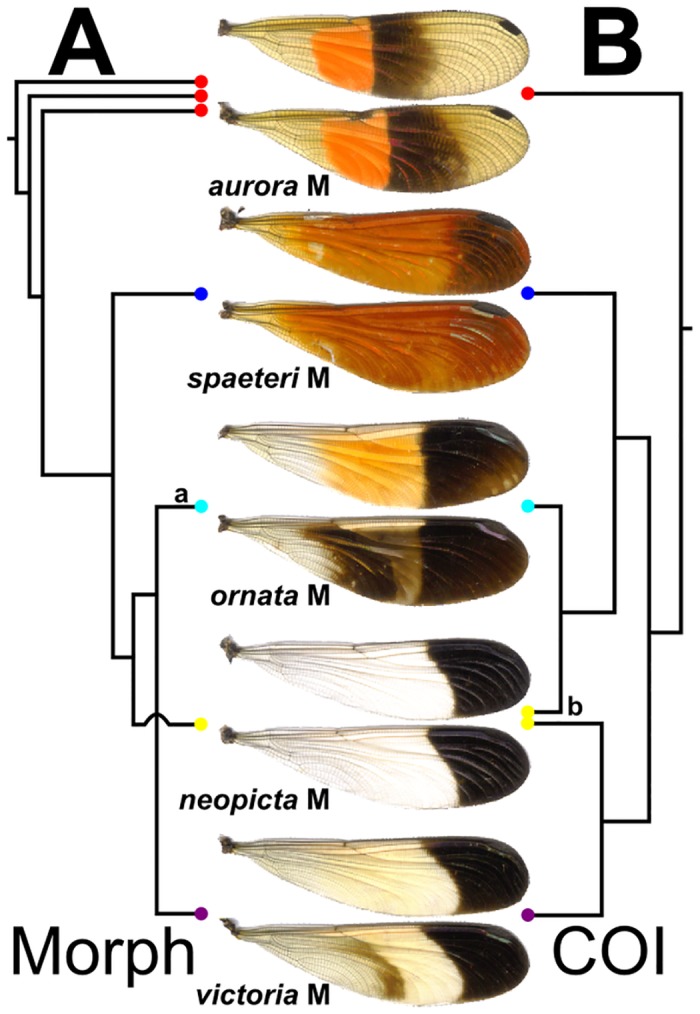
Comparison of the differing topologies among phylogenetic reconstructions. Simplified topologies are shown based on (A) morphological and (B) COI data, showing typical examples of each wing morph (center). Only males were included in these analyses. For full trees, see Fig 5 and S1 Fig. G. Note: (a) *P*. *ornata* was recovered as monophyletic (although with low, 9% bootstrap branch support) except for two specimens, which were recovered within *P*. *victoria* (see S1 Fig. G); (b) in B, some *P*. *neopicta* grouped with *P*. *ornata*, while others grouped with *P*. *victoria*.

While distinct pattern elements can be identified through our morphometric analyses, our phylogenetic results are less clear. When considering the phylogenetic analyses of the morphological dataset alone (Fig. G in S1 File), individuals sharing a common wingform generally form well-supported clades, reflecting the distinctness of the different wingforms. The greatest exception to this are the males of *P*. *aurora*, which are found to be paraphyletic with respect to the remaining species; this is perhaps due to the rather extreme variability in the color intensity of individuals in our analyses (see the 3 examples at the top of Fig 1), Two individual males of *P*. *ornata* clustered with *P*. *victoria* males in the morphological phylogeny; this is likely due to variation in the hindwing banding patterns and forewing color patterns. Our molecular phylogenetic analyses (Figs 2B and 7) and species delimitation analyses reveal that, for the Peruvian species studied here, there are some wingforms that correspond to well-defined species—such as *P*. *spaeteri* and *P*. *aurora*—while the wingforms associated with *P*. *victoria*, *P*. *neopicta* and *P*. *ornata* do not resolve well. *P*. *victoria* and *P*. *ornata* emerge in different locations within the tree, but have *P*. *neopicta* individuals contained within each of these two clades; a number of *P*. *victoria* and *P*. *neopicta* individuals cannot be separated at all, and this clade with its two wingforms is identified as a species in our delimitation analysis, albeit with low support. Other specimens of *P*. *neopicta* are indistinguishable from *P*. *ornata*, and these individuals are identified as a species with high support by our delimitation analysis. Further, our delimitation analysis suggests that, similar to the cryptic speciation within *P*. *procera* highlighted by Sánchez Herrera *et al*. (2010) and recovered in our own results (Fig 2B) we have a small clade of individuals from one site (Pozuzo 6) that, despite having the characteristic *P*. *victoria* wingform, are supported as a separate species from the other *P*. *victoria*. The lack of molecular separations between *P*. *neopicta* and *P*. *victoria*/*P*. *ornata* are at the heart of this lack of congruence between analyses. These preliminary analyses of the COI sequence suggest that a more extensive phylogenetic exploration may be necessary to elucidate species relationships.

Topography is potentially very important in the distribution of *Polythore*. The localities for *P*. *victoria*, *P*. *neopicta* and *P*. *ornata* are all within steep river valleys with high ridges separating them (Fig 2A); *P*. *spaeteri*, while at lower elevation between the Andes and Sira ranges, is also isolated from the other populations sampled in Peru. Thus, while the distances between these populations are not large, there are significant barriers to movement between these regions. In our haplotype network (Fig 6 and Table 2), *P*. *spaeteri* from Panguana, with its single wingform, shows relatively high genetic diversity in comparison to other groups that have multiple wingforms, but little morphological diversity. Other haplotypes within the Peruvian *Polythore* in our study appear to be derived from these *P*. *spaeteri* haplotypes, possibly due to the highest genetic diversity present in that population.

Most of the species in this study were taken from the drainage basin of the Río Ucayali, one of the most water-rich headwaters of the Amazon (Fig 2A); the one exception is *P*. *aurora* taken along the Río Marañón near Iquitos (see below). The *P*. *spaeteri* specimens in this study were physically closest to the Río Ucayali confluence, taken at the Panguana field station, on the Río Llullapichis, a tributary of the Rxo Pachitea, which discharges into the Río Ucayali. The other *Polythore* wingforms/species found in Peru, are all taken from river valleys (Pozuzo, Santa Cruz, Perené) that like the Pachitea, drain to the Río Ucayali. It is possible that there is some connectivity on the landscape through these river corridors, connecting *P*. *spaeteri* to the *P*. *neopicta*, *P*. *ornata* and *P*. *victoria* populations in higher-elevation streams, though some of the patterns seen from our high F_ST_ suggests that there is significant genetic isolation between the Pachitea river and Pozuzo River valleys (Figs 5 inset and 6). Individuals in the putative cryptic species with *P*. *victoria* wingforms identified by our delimitation analysis were taken from site Pozuzo 6, which is actually in the valley of the Río Santa Cruz, a tributary of the Río Pozuzo; this latter river valley is where all of the other sample populations with *P*. *victoria* wingforms are found. Within the higher-elevation *P*.*ornata* from Pampa Hermosa near the Perené River Valley shows a high genetic isolation with all the other populations from the Pozuzo Valley, except the Oxapampa. Finally, the Perené River Road population shows no differentiation with the Pozuzo Valley populations; this may represent an ancestral polymorphism, rather than more recent gene flow due to the lack of connectivity between these regions (Fig 2A). Populations containing these latter three wingforms (Fig 1, *P*. *neopicta*, *P*. *victoria* and *P*. *ornata*) are genetically less diverse, despite the greater morphological diversity (Fig 6 and Table 2).

The Ucayali flows north from central Peru to the Amazon and the region where *P*. *aurora* is found. It is also of note that the haplotype of *P*. *aurora*, is derived from the *P*. *neopicta*/*P*. *victoria* haplotype group, rather than from those of the Colombian *Polythore*; sampling of other *Polythore* populations in Peru would help to better elucidate this relationship, but it is again possible that river corridors may be involved in establishing flow between these populations.

Why do we see such high wing pattern diversity that does not correlate to our molecular phylogenetics? A few explanations are available for these patterns; one is that these groups are currently speciating, and as yet are difficult to distinguish through molecular genetic techniques [71–75]. If this is the explanation for our pattern, then wing color phenotypes have already diversified, but fully assortative mating has not yet established [76,77]. This is a potential explanation for the *P*. *neopicta* wingform, which does not emerge as a separate species in our analyses, but as polymorphic forms of two other species, *P*. *victoria* and *P*. *ornata*. If this is the case, then we have potentially two new species forming which share a common wing phenotype. There is also the possibility that wingform is associated with species identity, but that introgression between individuals with different wingforms occurs regularly. This scenario begs the question of why hybrid wingforms are not observed in these zones: regular mixing has apparently not diminished wing phenotypic diversity [78,79].

The final picture, drawn from our analyses, is of a group of damselflies with highly distinct, complex and divergent wing phenotypes, but for which molecular phylogenetic species determinations are not clear, suggesting significant levels of polymorphism within this group, as well as the possible existence of cryptic species, as also found by Sánchez Herrera and colleagues in their studies of *P*. *procera* in Colombia [34]. While this genus has a broad geographic distribution, local site wingform diversity is quite low, with usually only one predominant wingform found at any one locality. In some regions within the sampling area, a particular wingform is common in one zone, another common in an adjoining zone, and a narrow border zone exists between the two where the two wingforms are encountered; these wingforms appear to remain distinct, even in these overlapping zones. These distribution differences appear to persist across years and seasons, suggesting that these wing colors are not influenced by the age of the individual or climatic conditions. Our haplotype analysis suggests that there may be gene flow between some of these localities with different wingforms (for example, between the sites in the Pozuzo Valley where the *P*. *victoria* and *P*. *neopicta* clade is found) and as such we would consider these as a single population that is polymorphic.

In their recent review paper on the study of lineage divergence and speciation in insects, Mullen and Shaw suggest that a comprehensive understanding of the speciation process requires demonstrating the axes of differentiation in the system, the speciation phenotypes (i.e., traits whose divergence somehow limits gene flow, either directly or indirectly) and which evolutionary forces cause the divergence of a speciation phenotype, followed by an investigation of the genetic architecture of the speciation phenotypes and how they trigger further genome evolution in establishing species boundaries [80]. Here we have identified wing color patterning as a primary axis of differentiation, and the establishment of different wingforms as the phenotypes that may be associated with speciation. Now we must ask, what are the evolutionary forces driving this phenotypic divergence?

### Hypotheses to Explain Color Polymorphism in *Polythore*

Why did this extensive wing diversity arise in the first place? As has been noted previously, the majority of damselfly species that possess wing coloration display a single color in bands or spots on an otherwise clear wing, with much research supporting that this coloring functions to attracting mates or fend off conspecific male competitors [81,82]. If, similar to these other calopterygoid damselflies, sexual selection is also the main driver of wing coloration in *Polythore*, it remains to be explained why in this group it has resulted in such elaborate wing patterns. Further exploration of this potential selective force would require mating behavior experiments within and among individuals of *Polythore* with different wingforms. Some diversity in mating behavior, such as differences in tendency for males to mate guard while the female oviposits, have been observed among different *Polythore* forms (MSH and CDB, unpublished data) and quantitative natural observations combined with experimental pairing between males and females of different wingforms will determine how much wing coloration is a factor in mate choices in these damselflies. If it is found that mating preference is a function of wing color and pattern, this suggests that sexual selection is at least a factor maintaining wing diversity.

Another possibility that has been suggested (at least anecdotally) for *Polythore*, is that some of the wingforms may be under selection to resemble co-occurring toxic Ithomiinae and Heliconiinae (clearwing) butterflies [9,83–85] (K. Tennessen, pers. comm.). Even if wing colors were initially under sexual selection, selection by predators for similarity to local defended butterflies might have influenced wing color and pattern, in at least some species. It is of note that much recent work on speciation, introgression and wing ornamentation cited above comes from work on the *Heliconius* model system; comparative studies of the distribution of *Polythore* wingforms and butterfly wingforms may help to determine the feasibility of this hypothesis. Further investigation of this hypothesis will require analysis of the wing color and patterning of co-occuring butterflies in the regions where different *Polythore* wingforms are found. If selection for mimetic resemblance is a factor in this system, we predict that morphometric analysis of butterfly and damselfly wings would find significant correlations between these two groups in each locality where they are found, a requirement for the model/mimic relationship.

Work on the diversification and genomics of *Heliconius* butterflies has identified interesting patterns that are worth considering in the case of *Polythore*, regardless of whether mimetic resemblance may be involved in the evolution of the wing colors of these damselflies. Research suggests that *Heliconius* color patterning loci are tightly linked to alleles underlying variation in male preference[86]. In *Heliconius* tight physical linkage reduces recombination between loci associated with mating preference and color evolution and may facilitate the maintenance of positive assortative mating in this system. Introgression between different *Heliconius* species, while under strong selection from predators, may also provide the wing color diversity and adaptive novelty through wing color diversification[87], thus supporting the hypothesis that hybridization is an important source of adaptive novelty in this system. Considering what we now know about wing color diversity and species definitions in the *Polythore* damselflies, a further exploration of behavior, color diversity and the genomics of wing coloration in this group may elucidate more generally the factors that can influence the development of CP, in damselflies as well as other organisms.

## Supporting Information

S1 File Supplementary Figures and Tables. (DOCX)

S2 File *Polythore* polymorphism.zip. Compressed folder containing everything needed to run the analyses presented in this paper, including images, data, and a Mathematica notebook.
(ZIP)
