# Supplementary material for: Mixed Signals? Morphological and Molecular Evidence Suggest a Color Polymorphism in Some Neotropical Polythore Damselflies
Source: PLoS One. 2015 Apr 29;10(4):e0125074. doi: 10.1371/journal.pone.0125074 (PMC4414280; doi:10.1371/journal.pone.0125074)

**Supplementary Material:**

**Mixed Signals? Morphological and Molecular Evidence Suggest a Color Polymorphism in some the Neotropical *Polythore* Damselflies**

Sánchez Herrera, M., Kuhn, W.R., Lorenzo-Carballa, M.O., Harding, K.M., Ankrom, N., Sherratt, T.N., Hoffmann, J. , Van Gossum, H., Ware, J.L., Cordero-Rivera, A. & Beatty, C.D.

**List of Supplementary Table and Figures:**

**Table A. Localities and GenBank accession numbers for specimens used in this study.**

Listed are: voucher/specimen ID; species to which each specimen was initially assigned; sex; collection locality (abbreviation), coordinates, and elevation; GenBank accession number; and reference. Symbols next to specimen ID denote analyses in which that specimen was used: landmark and comparative morphological analyses (†), phylogenetic analysis from morphology (‡), and phylogenetic analysis from COI (*).

**Table B. F_ST_ and p-values among *Polythore* populations.**

Values correspond to those represented graphically in Fig. 7. See Fig. 7 legend and methods text for further explanation.

**Figure A.** **Example image of each wingform with landmarks present.**

50 landmarks were placed on each of the fore- and hindwings. See Methods and Figs. 2 and 4 for more explanation of landmark placement.

**Figure B.** **Example standardized square image.**

Specimen scans were standardized using a custom script in order to make them comparable to one another for the chromaticity and GWT analyses. In this process, wings were first extracted from the original scan and masked, then rotated so that the proximal margin was horizontal, rescaled to 512 px-wide, and arranged with fore- and hindwings on the upper and lower halves of the image, respectively.

**Figure C.** **Depiction of image sub-sampling in the chromaticity analysis**.

Sub-images are shown here on a standardized square image of a *P. ornata* male, which has been transformed to chromaticity coordinates and only the ‘r’ channel taken. Images were samples at 4 scales: (A) full square, (B) each wing separately, (C) thirds of each wing, and (D) sixths of each wing. See Methods for a more detailed explanation.

**Figure D.** **Depiction of Gabor wavelets and GWT image sub-sampling scheme.**

(A) Density plots of real and imaginary Gabor wavelets at the 3 angles used in this study; white pixels represent high values and black pixels, low values. (B) Placement of the circular Gabor wavelets (circles) over a square image (dashed); 4 scales of wavelets were used in the GWT analysis (full square, and 1/4, 1/16, and 1/64 of square). See Methods for a more detailed explanation.

**Figure E. Relative contributions (loadings) of coefficients in the first two DAPC axes for morphological analyses.**

(A) Landmarking, (B) Chromaticity, and (C) GWT analyses. The top 5 contributing coefficients are highlighted in red and correspond to the wing locations highlighted in Fig. 5D–F and S1 F.C.

**Figure F.** **Discriminant analysis of combined morphological analyses.**

(A) DAPC plot, (B) relative contributions, and (C) areas of the wings corresponding to the 5 most discriminating coefficients for DAPC axes 1 and 2 for the combined morphological dataset (landmarking, chromaticity, and GWT analyses). See Fig. 5 and Results text for explanation of C. Note: the top contributing coefficients (C) were all from the GWT analysis. Note: in B, coefficients 1–200 are from the landmarking analysis, 201–263 are from the chromaticity analysis and the remaining coefficients are from the GWT analysis.

**Figure G.** **Phylogenetic reconstruction based on morphological dataset.**

Reconstruction was performed using parsimony and bootstrapping (with 500 pseudoreplicates) in TNT and is shown here as a proportional phylogram. Values above branches are bootstrap support values; only values >50% are shown.

**File S2. *Polythore* polymorphism.zip** **(as a separate file).**

Compressed folder containing everything needed to run the analyses presented in this paper, including images, data, and a Mathematica notebook.

**Table A**

| **Specimen ID** | **Species** | **Sex** | **Locality** | **Coordinates** | **Elev. (m)** | **GenBank Acc. No.** | **Ref.** |
| --- | --- | --- | --- | --- | --- | --- | --- |
| ANDESE/MSH231 * | *P. gigantea* | ? | Colombia, Antioquia, Caldas, Quebrada |  |  | KP738303 | Sánchez Herrera et al., 2010 |
| ANDESE/LCG010 * | *P. gigantea* | ? | Colombia, Antioquia, Copacabana |  |  | KP738303 | Sánchez Herrera et al., 2010 |
| ANDESE/MSH206 * | *P. gigantea* | ? | Colombia, Antioquia, Quebrada La Doctora |  |  | KP738302 | Sánchez Herrera et al., 2010 |
| ANDESE/MSH212 * | *P. gigantea* | ? | Colombia, Antioquia, Quebrada La Doctora |  |  | KP738301 | Sánchez Herrera et al., 2010 |
| ANDESE/MSH220 * | *P. gigantea* | ? | Colombia, Antioquia, Quebrada La Miel |  |  | KP738304 | Sánchez Herrera et al., 2010 |
| ANDESE/MSH183 * | *P. procera* | ? | Colombia, Cundinamarca, Cementerio |  |  | FJ514980 | Sánchez Herrera et al., 2010 |
| ANDESE/MSH185 * | *P. procera* | ? | Colombia, Cundinamarca, Cementerio |  |  | FJ514982 | Sánchez Herrera et al., 2010 |
| ANDESE/MSH187 * | *P. procera* | ? | Colombia, Cundinamarca, Cementerio |  |  | FJ514984 | Sánchez Herrera et al., 2010 |
| ANDESE/MSH189 * | *P. procera* | ? | Colombia, Cundinamarca, Cementerio |  |  | FJ514986 | Sánchez Herrera et al., 2010 |
| ANDESE/MSH130 * | *P. procera* | ? | Colombia, Cundinamarca, Quebrada La Catira |  |  | FJ514944 | Sánchez Herrera et al., 2010 |
| ANDESE/MSH132 * | *P. procera* | ? | Colombia, Cundinamarca, Quebrada La Catira |  |  | FJ514946 | Sánchez Herrera et al., 2010 |
| ANDESE/MSH134 * | *P. procera* | ? | Colombia, Cundinamarca, Quebrada La Catira |  |  | FJ514948 | Sánchez Herrera et al., 2010 |
| ANDESE/MSH138 * | *P. procera* | ? | Colombia, Cundinamarca, Quebrada La Catira |  |  | FJ514950 | Sánchez Herrera et al., 2010 |
| ANDESE/MSH140 * | *P. procera* | ? | Colombia, Cundinamarca, Quebrada La Catira |  |  | FJ514952 | Sánchez Herrera et al., 2010 |
| ANDESE/MSH170 * | *P. procera* | ? | Colombia, Cundinamarca, Quebrada La Catira |  |  | FJ514968 | Sánchez Herrera et al., 2010 |
| ANDESE/MSH162 * | *Euthore fasciata* | ? | Colombia, Cundinamarca, Vereda Virgen de Chirajara |  |  | n.a. | Sánchez Herrera et al., 2010 |
| ANDESE/MSH149 * | *Polythore* sp. | ? | Colombia, Cundinamarca, Vereda Virgen de Chirajara |  |  | FJ514960 | Sánchez Herrera et al., 2010 |
| ANDESE/MSH154 * | *Polythore* sp. | ? | Colombia, Cundinamarca, Vereda Virgen de Chirajara |  |  | FJ514964 | Sánchez Herrera et al., 2010 |
| ANDESE/MSH158 * | *Polythore* sp. | ? | Colombia, Cundinamarca, Vereda Virgen de Chirajara |  |  | FJ514966 | Sánchez Herrera et al., 2010 |
| ANDESE/MSH200 * | *Polythore* sp. | ? | Colombia, Cundinamarca, Vereda Virgen de Chirajara |  |  | FJ514996 | Sánchez Herrera et al., 2010 |
| ANDESE/MSH176 * | *P. procera* | ? | Colombia, Villavicencio, Bosque Bavaria |  |  | FJ514974 | Sánchez Herrera et al., 2010 |
| ANDESE/MSH191 * | *P. procera* | ? | Colombia, Villavicencio, Bosque Bavaria |  |  | FJ514988 | Sánchez Herrera et al., 2010 |
| ANDESE/MSH193 * | *P. procera* | ? | Colombia, Villavicencio, Bosque Bavaria |  |  | FJ514990 | Sánchez Herrera et al., 2010 |
| ANDESE/MSH195 * | *P. procera* | ? | Colombia, Villavicencio, Bosque Bavaria |  |  | FJ514992 | Sánchez Herrera et al., 2010 |
| ANDESE/MSH197 * | *P. procera* | ? | Colombia, Villavicencio, Bosque Bavaria |  |  | FJ514994 | Sánchez Herrera et al., 2010 |
| P-88 †‡ | *P. aurora* | M | Peru (northern), Iquitos (IQT) | S 3^o^ 47.4' W 73^o^ 20.4' | 90 | n.a. | This study |
| P-89 †‡ | *P. aurora* | M | Peru (northern), Iquitos (IQT) | S 3^o^ 47.4' W 73^o^ 20.4' | 90 | n.a. | This study |
| Pa05 †‡* | *P. aurora* | M | Peru (northern), Iquitos (IQT) | S 3^o^ 47.4' W 73^o^ 20.4' | 90 | KP738289 | This study |
| Pa87 †‡* | *P. aurora* | M | Peru (northern), Iquitos (IQT) | S 3^o^ 47.4' W 73^o^ 20.4' | 90 | KP738290 | This study |
| Pl10 †‡* | *P. neopicta* | M | Peru, Oxapampa (OX) | S 10^o^ 48.6' W 75^o^ 19.8' | 879 | KP738291 | This study |
| Pl39 †‡* | *P. neopicta* | M | Peru, Oxapampa (OX) | S 10^o^ 48.6' W 75^o^ 19.8' | 879 | KP738295 | This study |
| Po42 †‡* | *P. ornata* | M | Peru, Oxapampa (OX) | S 10^o^ 48.6' W 75^o^ 19.8' | 879 | KP738307 | This study |
| P-24a †‡ | *P. ornata* | M | Peru, Pampa Hermosa (PMH) | S 11^o^ 4.8' W 75^o^ 27.6' | 1342 | n.a. | This study |
| P-24b † | *P. ornata* | F | Peru, Pampa Hermosa (PMH) | S 11^o^ 4.8' W 75^o^ 27.6' | 1342 | n.a. | This study |
| P-25a †‡ | *P. ornata* | M | Peru, Pampa Hermosa (PMH) | S 11^o^ 4.8' W 75^o^ 27.6' | 1342 | n.a. | This study |
| P-25b †‡ | *P. ornata* | M | Peru, Pampa Hermosa (PMH) | S 11^o^ 4.8' W 75^o^ 27.6' | 1342 | n.a. | This study |
| P-25c †‡ | *P. ornata* | M | Peru, Pampa Hermosa (PMH) | S 11^o^ 4.8' W 75^o^ 27.6' | 1342 | n.a. | This study |
| P-26a † | *P. ornata* | F | Peru, Pampa Hermosa (PMH) | S 11^o^ 4.8' W 75^o^ 27.6' | 1342 | n.a. | This study |
| P-26b † | *P. ornata* | F | Peru, Pampa Hermosa (PMH) | S 11^o^ 4.8' W 75^o^ 27.6' | 1342 | n.a. | This study |
| P-26c † | *P. ornata* | F | Peru, Pampa Hermosa (PMH) | S 11^o^ 4.8' W 75^o^ 27.6' | 1342 | n.a. | This study |
| P-32a †‡ | *P. ornata* | M | Peru, Pampa Hermosa (PMH) | S 11^o^ 4.8' W 75^o^ 27.6' | 1342 | n.a. | This study |
| P-32b †‡ | *P. ornata* | M | Peru, Pampa Hermosa (PMH) | S 11^o^ 4.8' W 75^o^ 27.6' | 1342 | n.a. | This study |
| Po20a †‡* | *P. ornata* | M | Peru, Pampa Hermosa (PMH) | S 11^o^ 4.8' W 75^o^ 27.6' | 1357 | KP738297 | This study |
| Po20b †‡* | *P. ornata* | M | Peru, Pampa Hermosa (PMH) | S 11^o^ 4.8' W 75^o^ 27.6' | 1357 | KP738298 | This study |
| Po20c †‡* | *P. ornata* | M | Peru, Pampa Hermosa (PMH) | S 11^o^ 4.8' W 75^o^ 27.6' | 1357 | KP738299 | This study |
| Po30a †‡ | *P. ornata* | M | Peru, Pampa Hermosa (PMH) | S 11^o^ 4.8' W 75^o^ 27.6' | 1342 | n.a. | This study |
| Po30a †‡* | *P. ornata* | M | Peru, Pampa Hermosa (PMH) | S 11^o^ 4.8' W 75^o^ 27.6' | 1342 | KP738300 | This study |
| Po30c †‡* | *P. ornata* | M | Peru, Pampa Hermosa (PMH) | S 11^o^ 4.8' W 75^o^ 27.6' | 1342 | KP738306 | This study |
| Po43 †‡* | *P. ornata* | M | Peru, Pampa Hermosa (PMH) | S 11^o^ 4.8' W 75^o^ 27.6' | 1348 | KP738308 | This study |
| Po45 †‡* | *P. ornata* | M | Peru, Pampa Hermosa (PMH) | S 11^o^ 4.8' W 75^o^ 27.6' | 1342 | KP738309 | This study |
| Po47a †‡* | *P. ornata* | M | Peru, Pampa Hermosa (PMH) | S 11^o^ 4.8' W 75^o^ 27.6' | 1357 | KP738310 | This study |
| Po47b †‡* | *P. ornata* | M | Peru, Pampa Hermosa (PMH) | S 11^o^ 4.8' W 75^o^ 27.6' | 1357 | KP738311 | This study |
| Po49 * | *P. ornata* | M | Peru, Pampa Hermosa (PMH) | S 11^o^ 4.8' W 75^o^ 27.6' | 1357 | KP738312 | This study |
| P-43 †‡ | *P. spaeteri* | M | Peru, Panguana (PAN) | S 9^o^ 37.2' W 74^o^ 55.8' | 261 | n.a. | This study |
| P-44 † | *P. spaeteri* | F | Peru, Panguana (PAN) | S 9^o^ 37.2' W 74^o^ 55.8' | 261 | n.a. | This study |
| P-49 †‡ | *P. spaeteri* | M | Peru, Panguana (PAN) | S 9^o^ 37.2' W 74^o^ 55.8' | 261 | n.a. | This study |
| P-50 † | *P. spaeteri* | F | Peru, Panguana (PAN) | S 9^o^ 37.2' W 74^o^ 55.8' | 261 | n.a. | This study |
| P-94 †‡ | *P. spaeteri* | M | Peru, Panguana (PAN) | S 9^o^ 37.2' W 74^o^ 55.8' | 261 | n.a. | This study |
| Ps06 †‡* | *P. spaeteri* | M | Peru, Panguana (PAN) | S 9^o^ 37.2' W 74^o^ 55.8' | 261 | KP738327 | This study |
| Ps07 †‡* | *P. spaeteri* | M | Peru, Panguana (PAN) | S 9^o^ 37.2' W 74^o^ 55.8' | 261 | KP738328 | This study |
| Ps08 †‡* | *P. spaeteri* | M | Peru, Panguana (PAN) | S 9^o^ 37.2' W 74^o^ 55.8' | 261 | KP738329 | This study |
| Ps09 †‡* | *P. spaeteri* | M | Peru, Panguana (PAN) | S 9^o^ 37.2' W 74^o^ 55.8' | 270 | KP738330 | This study |
| Ps54 †‡* | *P. spaeteri* | M | Peru, Panguana (PAN) | S 9^o^ 37.2' W 74^o^ 55.8' | 269 | KP738331 | This study |
| Ps57 †‡* | *P. spaeteri* | M | Peru, Panguana (PAN) | S 9^o^ 37.2' W 74^o^ 55.8' | 270 | KP738332 | This study |
| Ps58 * | *P. spaeteri* | F | Peru, Panguana (PAN) | S 9^o^ 37.2' W 74^o^ 55.8' | 263 | KP738333 | This study |
| Ps90 †‡* | *P. spaeteri* | M | Peru, Panguana (PAN) | S 9^o^ 37.2' W 74^o^ 55.8' | 227 | KP738334 | This study |
| PsAM180640 * | *P. spaeteri* | ? | Peru, Panguana (PAN) | S 9^o^ 37.2' W 74^o^ 55.8' | 261 | AM180640 | Etscher et al., 2006 |
| PsAM180641 * | *P. spaeteri* | ? | Peru, Panguana (PAN) | S 9^o^ 37.2' W 74^o^ 55.8' | 261 | AM180641 | Etscher et al., 2006 |
| PsAM180642 * | *P. spaeteri* | ? | Peru, Panguana (PAN) | S 9^o^ 37.2' W 74^o^ 55.8' | 261 | AM180642 | Etscher et al., 2006 |
| PsAM180643 * | *P. spaeteri* | ? | Peru, Panguana (PAN) | S 9^o^ 37.2' W 74^o^ 55.8' | 261 | AM180643 | Etscher et al., 2006 |
| PsAM180644 * | *P. spaeteri* | ? | Peru, Panguana (PAN) | S 9^o^ 37.2' W 74^o^ 55.8' | 261 | AM180644 | Etscher et al., 2006 |
| PsAM180645 * | *P. spaeteri* | ? | Peru, Panguana (PAN) | S 9^o^ 37.2' W 74^o^ 55.8' | 261 | AM180645 | Etscher et al., 2006 |
| Pl35 †‡* | *P. neopicta* | M | Peru, Perené River Road (PER) | S 10^o^ 57' W 75^o^ 18.6' | 808 | KP738292 | This study |
| Pl37 †‡* | *P. neopicta* | M | Peru, Perené River Road (PER) | S 10^o^ 57' W 75^o^ 16.2' | 742 | KP738293 | This study |
| Pl38 * | *P. neopicta* | F | Peru, Perené River Road (PER) | S 10^o^ 57' W 75^o^ 16.2' | 814 | KP738294 | This study |
| Pl39d †‡* | *P. neopicta* | M | Peru, Perené River Road (PER) | S 10^o^ 57' W 75^o^ 18.6' | 808 | KP738296 | This study |
| Pp26a †‡* | *P. neopicta* | M | Peru, Pozuzo (POZ01) | S 9^o^ 53.4' W 75^o^ 31.8' | 609 | KP738316 | This study |
| Pp26b * | *P. neopicta* | F | Peru, Pozuzo (POZ01) | S 9^o^ 53.4' W 75^o^ 31.8' | 609 | KP738317 | This study |
| Pp84 * | *P. neopicta* | F | Peru, Pozuzo (POZ01) | S 9^o^ 53.4' W 75^o^ 31.8' | 650 | KP738326 | This study |
| Pp9 †‡* | *P. neopicta* | M | Peru, Pozuzo (POZ01) | S 9^o^ 53.4' W 75^o^ 31.8' | 609 | KP738313 | This study |
| Pp23 †‡* | *P. neopicta* | M | Peru, Pozuzo (POZ02) | S 9^o^ 55.2' W 75^o^ 31.8' | 662 | KP738314 | This study |
| Pp24 †‡* | *P. neopicta* | M | Peru, Pozuzo (POZ02) | S 9^o^ 55.2' W 75^o^ 31.8' | 662 | KP738315 | This study |
| Pp28 †‡* | *P. neopicta* | M | Peru, Pozuzo (POZ02) | S 9^o^ 55.2' W 75^o^ 31.8' | 662 | KP738318 | This study |
| Pp29 †‡* | *P. neopicta* | M | Peru, Pozuzo (POZ02) | S 9^o^ 55.2' W 75^o^ 31.8' | 662 | KP738319 | This study |
| Pp30 †‡* | *P. neopicta* | M | Peru, Pozuzo (POZ02) | S 9^o^ 55.2' W 75^o^ 31.8' | 662 | KP738320 | This study |
| Pp67 †‡* | *P. neopicta* | M | Peru, Pozuzo (POZ02) | S 9^o^ 55.2' W 75^o^ 31.8' | 662 | KP738321 | This study |
| Pp68 †‡* | *P. neopicta* | M | Peru, Pozuzo (POZ02) | S 9^o^ 55.2' W 75^o^ 31.8' | 671 | KP738322 | This study |
| Pp69 †‡* | *P. neopicta* | M | Peru, Pozuzo (POZ02) | S 9^o^ 55.2' W 75^o^ 31.8' | 662 | KP738323 | This study |
| Pv19 †‡* | *P. victoria* | M | Peru, Pozuzo (POZ02) | S 9^o^ 55.2' W 75^o^ 31.8' | 655 | KP738340 | This study |
| Pv20 †‡* | *P. victoria* | M | Peru, Pozuzo (POZ02) | S 9^o^ 55.2' W 75^o^ 31.8' | 655 | KP738341 | This study |
| Pv72 †‡* | *P. victoria* | M | Peru, Pozuzo (POZ02) | S 9^o^ 55.2' W 75^o^ 31.8' | 655 | KP738342 | This study |
| Pv83 †‡* | *P. victoria* | M | Peru, Pozuzo (POZ02) | S 9^o^ 55.2' W 75^o^ 31.8' | 655 | KP738343 | This study |
| Pp77 †‡* | *P. neopicta* | M | Peru, Pozuzo (POZ03) | S 9^o^ 52.2' W 75^o^ 31.8' | 575 | KP738324 | This study |
| Pp78 †‡* | *P. neopicta* | M | Peru, Pozuzo (POZ03) | S 9^o^ 52.2' W 75^o^ 31.8' | 575 | KP738325 | This study |
| Pv12 †‡* | *P. victoria* | M | Peru, Pozuzo (POZ04) | S 10^o^ 9' W 75^o^ 33.6' | 901 | KP738335 | This study |
| Pv13 †‡* | *P. victoria* | M | Peru, Pozuzo (POZ04) | S 10^o^ 9' W 75^o^ 33.6' | 901 | KP738336 | This study |
| Pv16 †‡* | *P. victoria* | M | Peru, Pozuzo (POZ04) | S 10^o^ 9' W 75^o^ 33.6' | 901 | KP738338 | This study |
| Pv15 †‡* | *P. victoria* | M | Peru, Pozuzo (POZ05) | S 10^o^ 24' W 75^o^ 33' | 901 | KP738337 | This study |
| Pv18 †‡* | *P. victoria* | M | Peru, Pozuzo (POZ06) | S 10^o^ 1.8' W 75^o^ 37.8' | 832 | KP738339 | This study |
| Pv85 †‡* | *P. victoria* | M | Peru, Pozuzo (POZ06) | S 10^o^ 1.8' W 75^o^ 37.8' | 832 | KP738344 | This study |
| P-65 †‡ | *P. victoria* | M | Peru, Pozuzo (POZ09) | S 10^o^ 8.4' W 75^o^ 32.7' |  | n.a. | This study |
| P-79a † | *P. victoria* | F | Peru, Pozuzo (POZ09) | S 10^o^ 8.4' W 75^o^ 32.7’ |  | n.a. | This study |
| P-79b † | *P. victoria* | F | Peru, Pozuzo (POZ09) | S 10^o^ 8.4' W 75^o^ 32.7’ |  | n.a. | This study |
| P-21 †‡ | *P. victoria* | M | Peru, Pozuzo (POZ12) | S 10^o^ 9.1' W 75^o^ 33.4’ |  | n.a. | This study |
| P-79 †‡ | *P. victoria* | M | Peru, Pozuzo (POZ12) | S 10^o^ 9.1' W 75^o^ 33.4’ |  | n.a. | This study |
| P-66 †‡ | *P. victoria* | M | Peru, Pozuzo (POZ13) | S 10^o^ 8.5' W 75^o^ 32.9’ |  | n.a. | This study |
| P-5 † | *P. neopicta* | F | Peru, Pozuzo (POZ15) | S 9^o^ 52.6' W 75^o^ 31.9’ |  | n.a. | This study |
| P-2 †‡ | *P. neopicta* | M | Peru, Pozuzo (POZ22) | S 9^o^ 54.9' W 75^o^ 31.6’ |  | n.a. | This study |
| P-3a †‡ | *P. neopicta* | M | Peru, Pozuzo (POZ22) | S 9^o^ 54.9' W 75^o^ 31.6’ |  | n.a. | This study |
| P-3b †‡ | *P. neopicta* | M | Peru, Pozuzo (POZ22) | S 9^o^ 54.9' W 75^o^ 31.6’ |  | n.a. | This study |
| P-81 †‡ | *P. neopicta* | M | Peru, Pozuzo (POZ22) | S 9^o^ 54.9' W 75^o^ 31.6’ |  | n.a. | This study |
| P-82 †‡ | *P. neopicta* | M | Peru, Pozuzo (POZ22) | S 9^o^ 54.9' W 75^o^ 31.6’ |  | n.a. | This study |
| P-7a † | *P. neopicta* | F | Peru, Pozuzo (POZ30) | S 9^o^ 53.5' W 75^o^ 31.9’ |  | n.a. | This study |
| P-7b † | *P. neopicta* | F | Peru, Pozuzo (POZ30) | S 9^o^ 53.5' W 75^o^ 31.9’ |  | n.a. | This study |

**Table B**

|  | p-values (above diagonal) and F_ST_ values (below diagonal) | | | | | | | | | | |
| --- | --- | --- | --- | --- | --- | --- | --- | --- | --- | --- | --- |
| Population | Perene River | Oxapampa | Iquitos | Panguana | PHermosa | POZ3 | POZ4 | POZ5 | POZ6 | POZ1 | POZ2 |
| Perene River |  | **0.01802** | 0.08108 | **0.00000** | **0.00000** | 0.99099 | **0.02703** | 0.99099 | **0.04505** | 0.99099 | 0.13514 |
| Oxapampa | **0.96765*** |  | 0.07207 | **0.00000** | **0.99099** | 0.14414 | 0.08108 | 0.99099 | 0.11712 | **0.01802** | **0.00000** |
| Iquitos | 0.99163 | 1 |  | **0.00901** | **0.00901** | 0.36937 | 0.10811 | 0.99099 | 0.31532 | **0.01802** | **0.01802** |
| Panguana | **0.90279*** | **0.83737*** | **0.9799*** |  | **0.00000** | **0.00000** | **0.00000** | 0.99099 | **0.01802** | **0.00000** | **0.00000** |
| PHermosa | **0.9874*** | **0** | **1*** | **0.8904*** |  | **0.03604** | **0.00901** | 0.99099 | **0.00901** | **0.00000** | **0.00000** |
| POZ3 | -0.26316 | 1 | 1 | **0.89846*** | **1*** |  | 0.05405 | 0.99099 | 0.38739 | 0.99099 | 0.99099 |
| POZ4 | **0.53153*** | 0.98 | 0.99514 | **0.89626*** | **0.99328*** | 0.67568 |  | 0.99099 | 0.09910 | 0.09910 | **0.00000** |
| POZ5 | 0.6 | 1 | 1 | **0.88822*** | **1*** | 1 | 0 |  | 0.39640 | 0.13514 | 0.07207 |
| POZ6 | **0.91273*** | 1 | 1 | 0.8863 | 1 | 1 | 0.9397 | 1 |  | 0.06306 | **0.00000** |
| POZ1 | 0 | **0.98312*** | **0.99585*** | **0.9045*** | **0.99347*** | -0.26316 | 0.47287 | 0.71429 | 0.9519 |  | 0.99099 |
| POZ2 | 0.1773 | **0.99214*** | **0.9983*** | **0.93522*** | **0.99513*** | -0.32663 | **0.78711*** | 0.91304 | **0.98078*** | -0.05263 |  |

**Figure A**

**
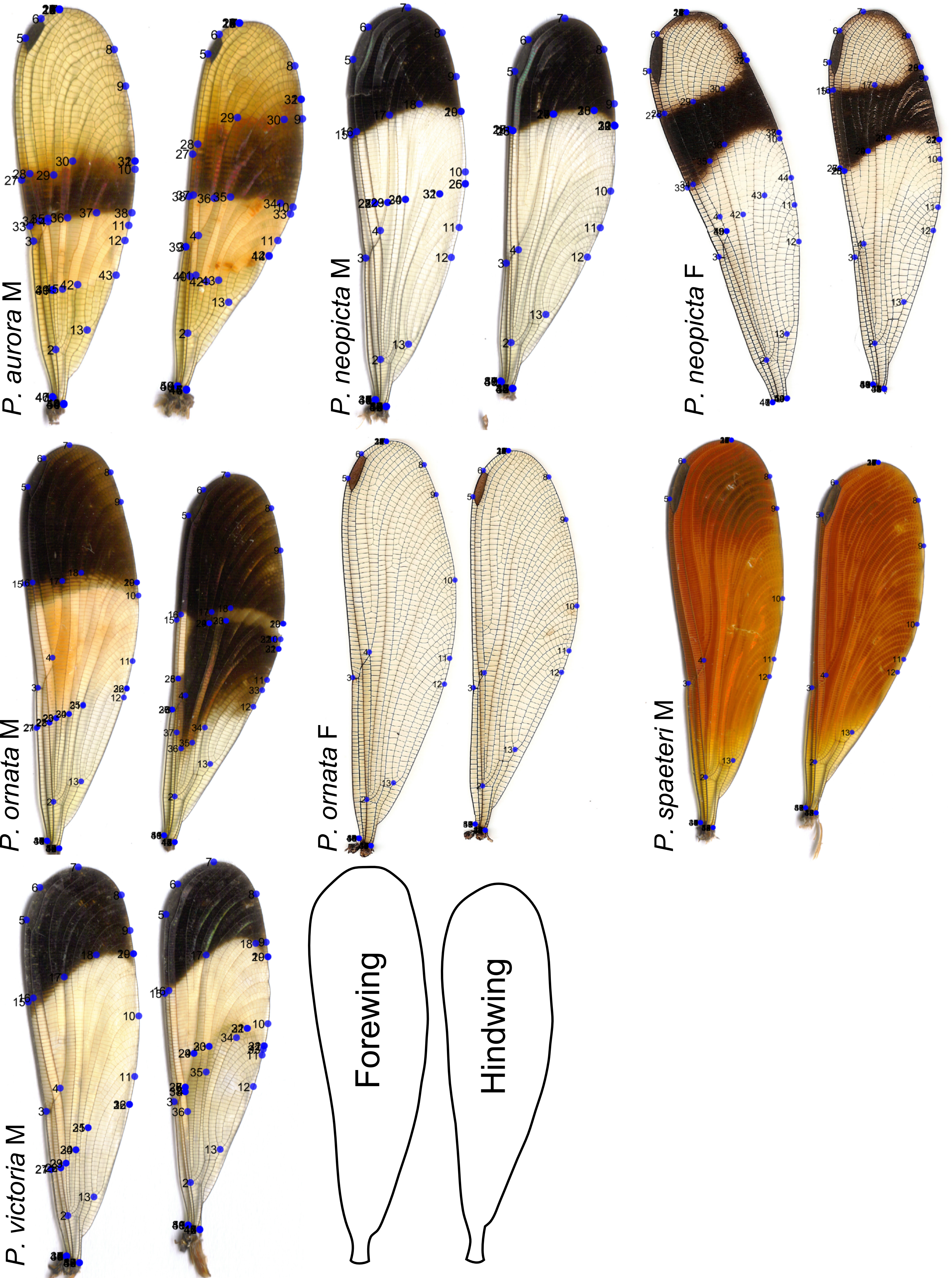
**

**Figure B**


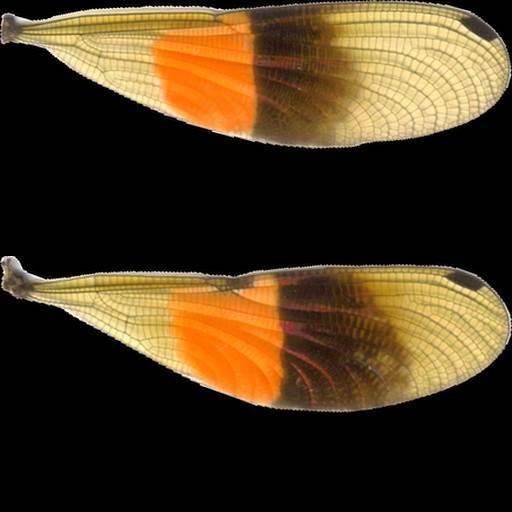


**Figure C**

**
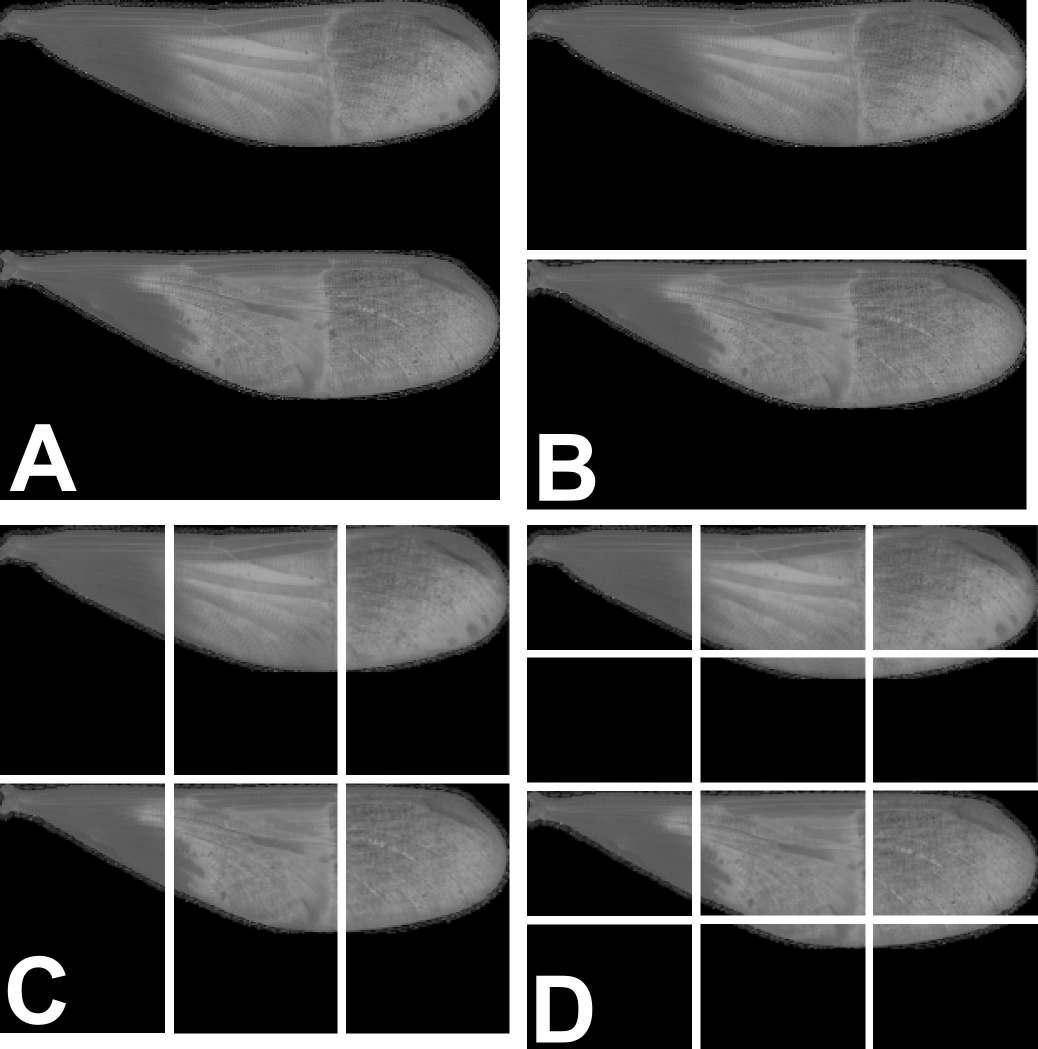
**

**Figure D**

**
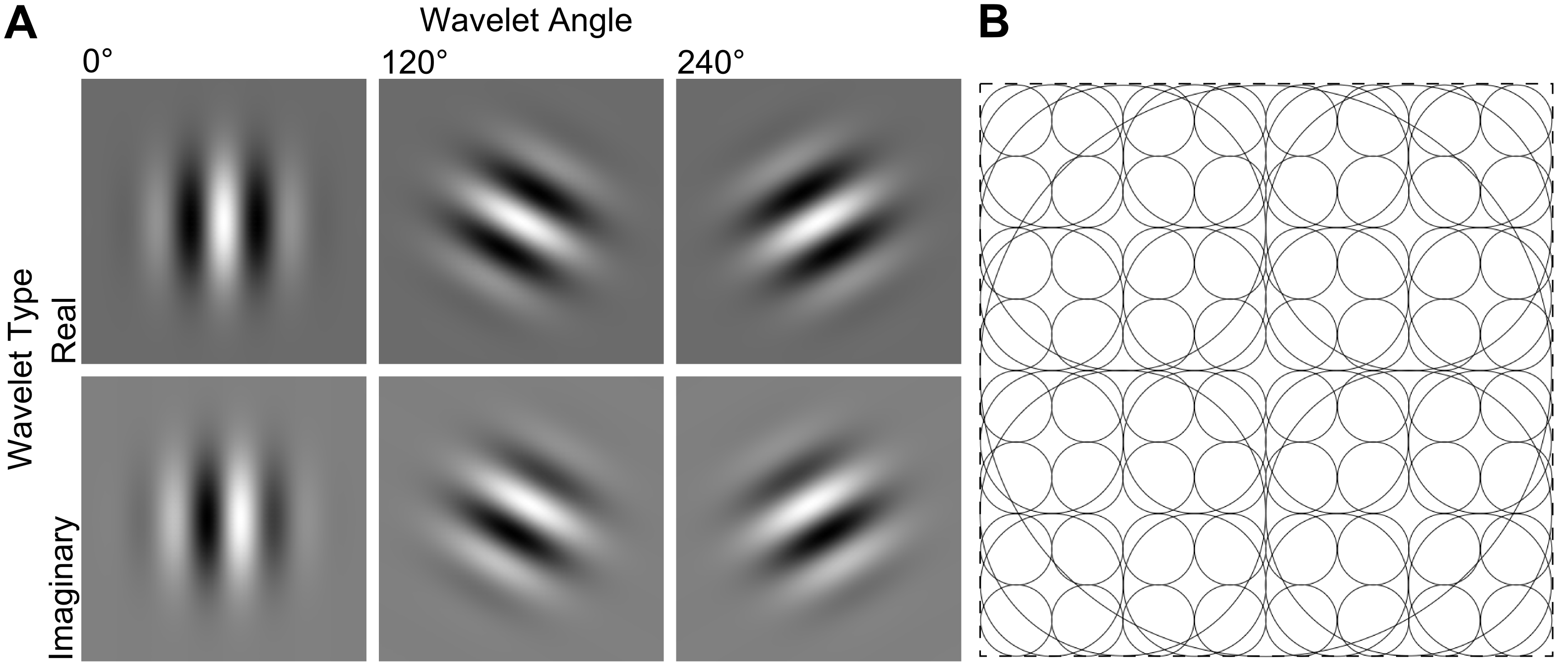
**

**Figure E**

**
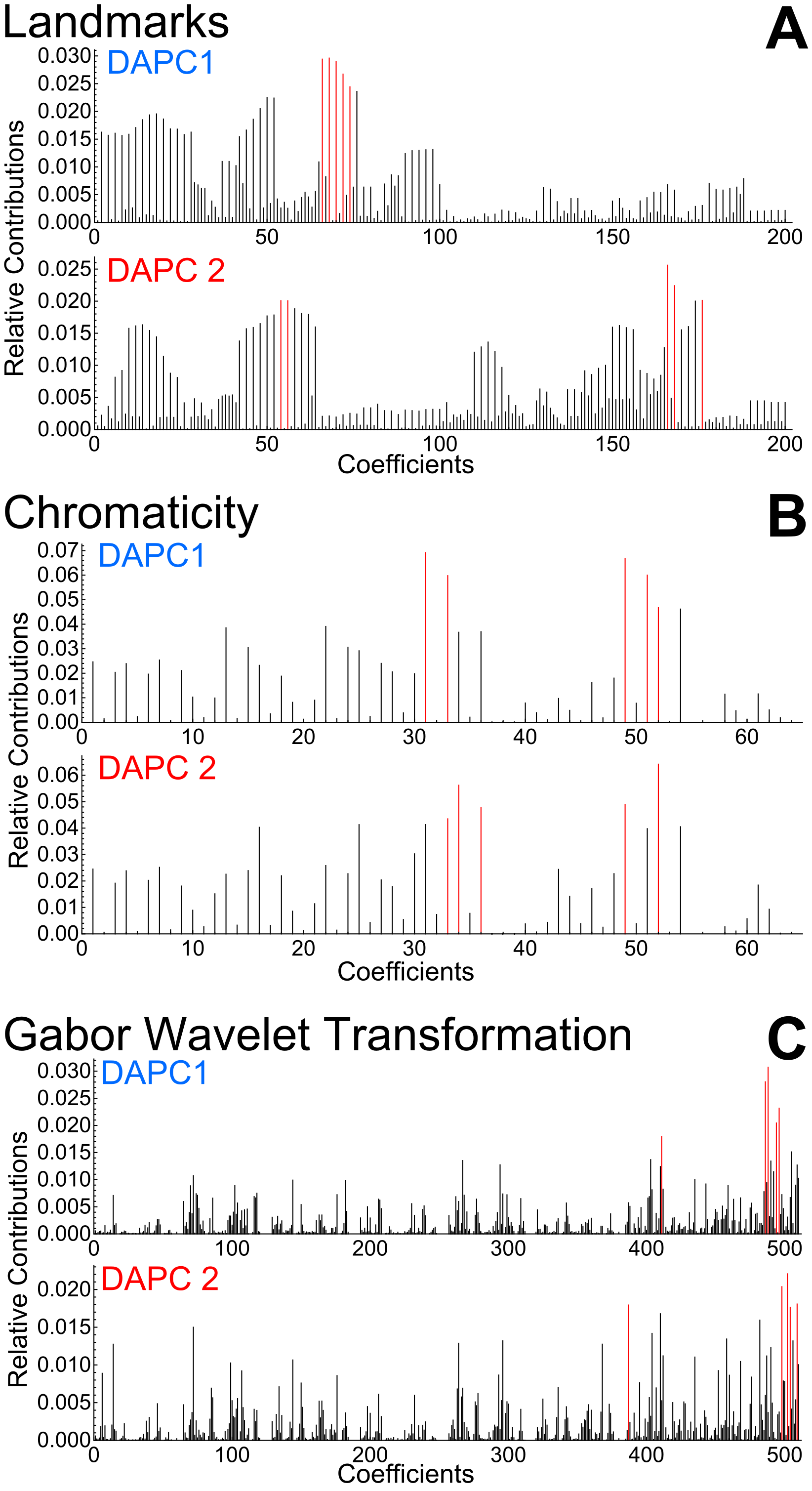
**

**Figure F**


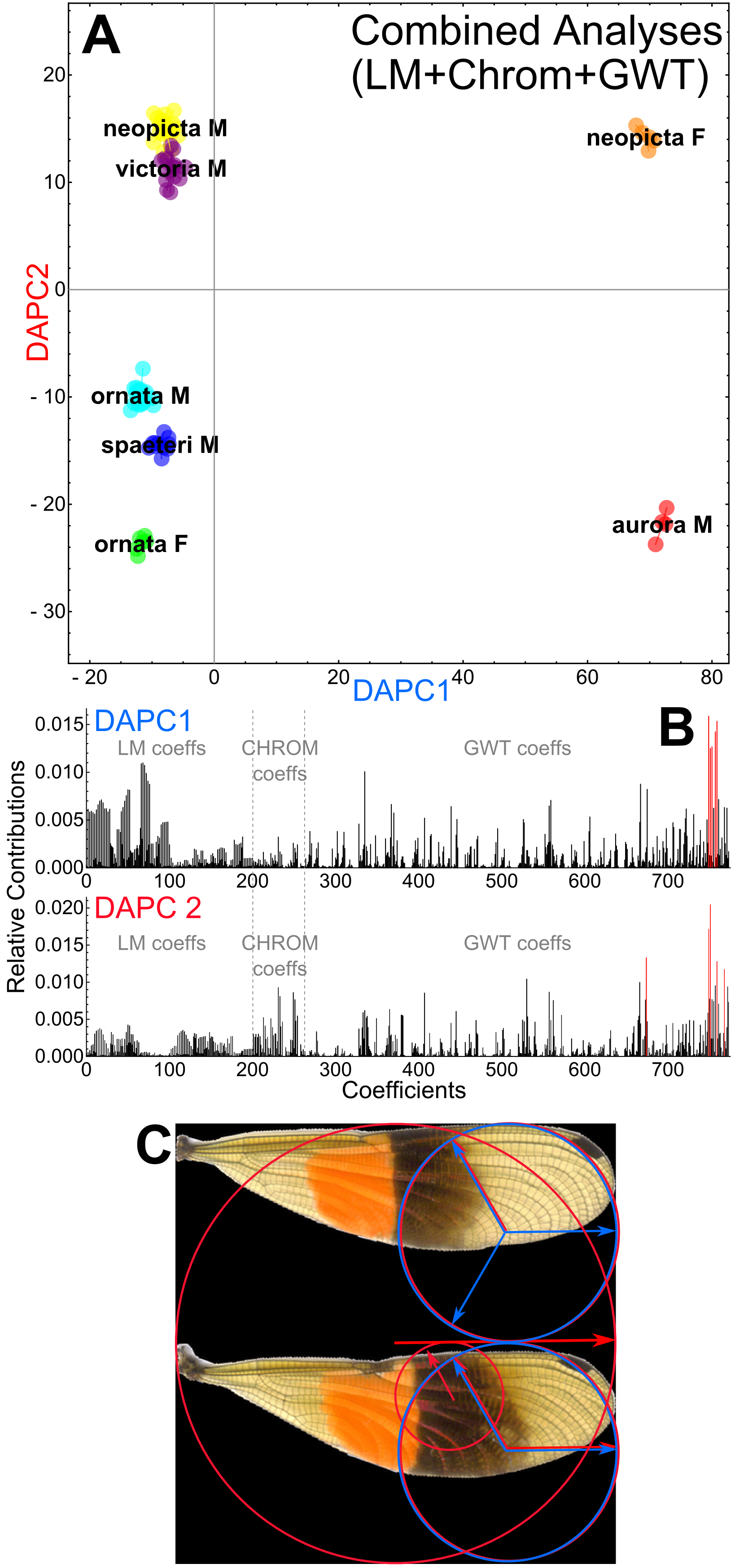


**Figure G**


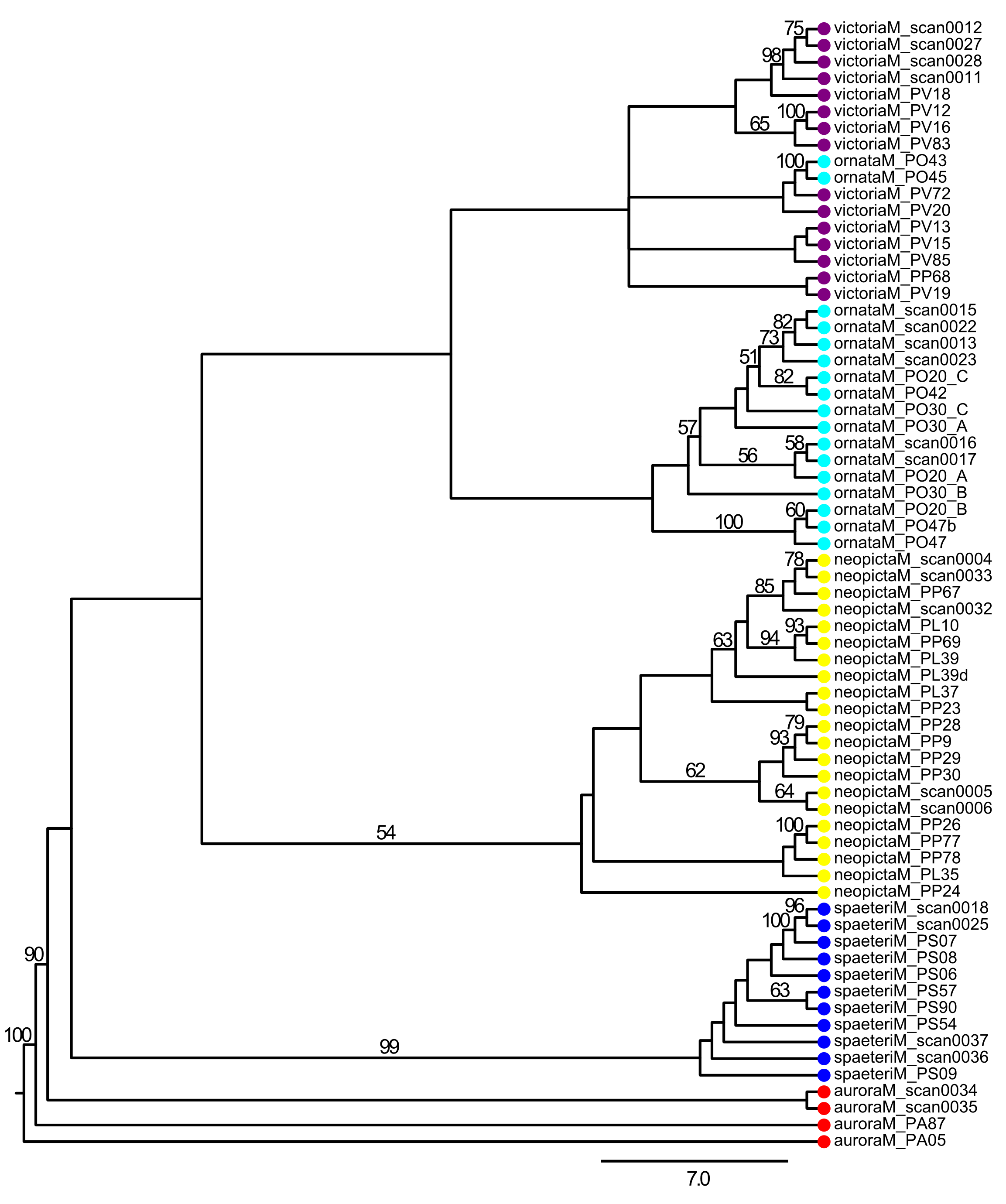

Supplement: S1 File — (DOCX) [file pone.0125074.s001.docx]
