# Supplementary figures and images for: Mixed Signals? Morphological and Molecular Evidence Suggest a Color Polymorphism in Some Neotropical Polythore Damselflies
Source: PLoS One. 2015 Apr 29;10(4):e0125074. doi: 10.1371/journal.pone.0125074 (PMC4414280; doi:10.1371/journal.pone.0125074)

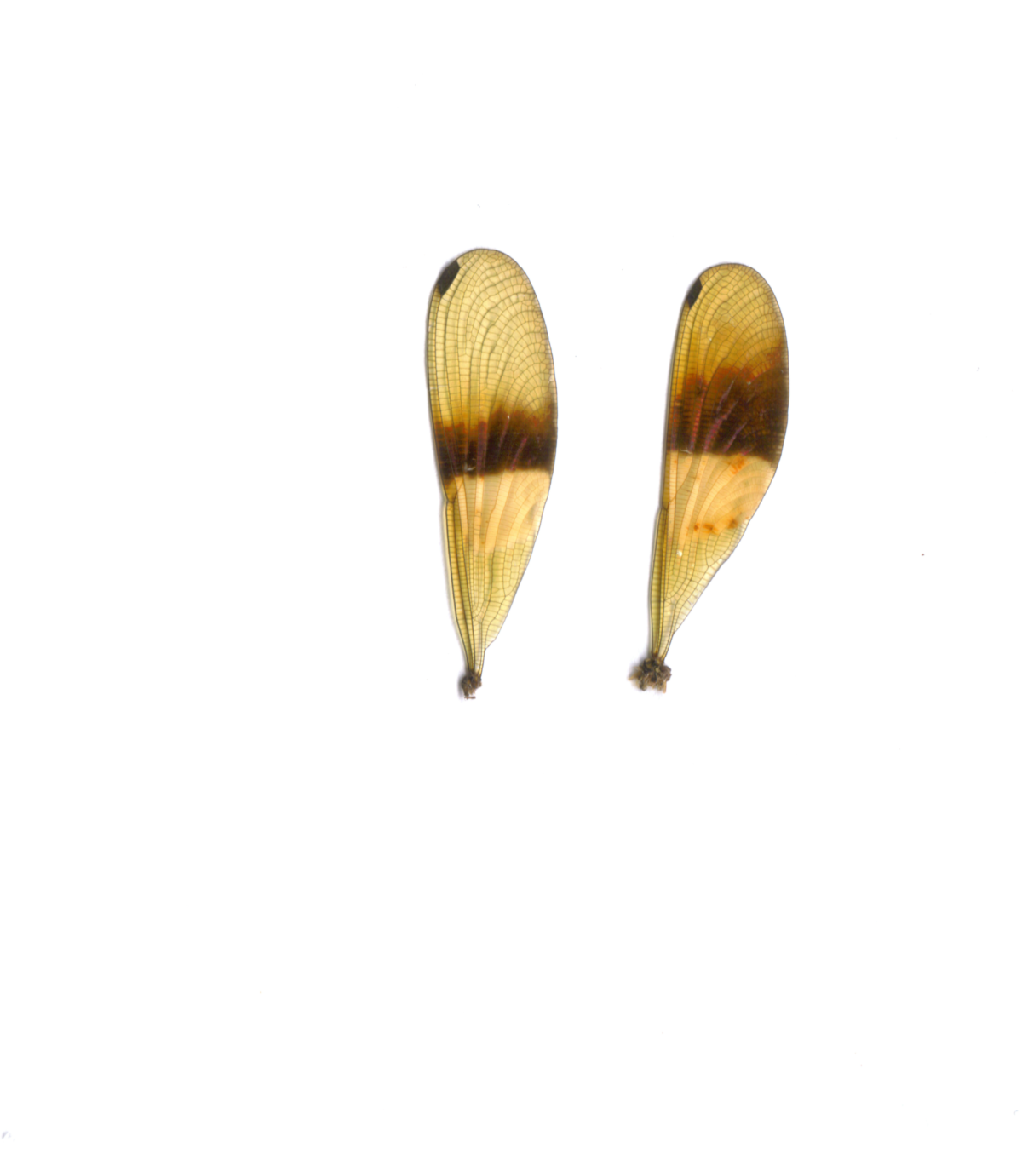

Supplement: S2 File — Compressed folder containing everything needed to run the analyses presented in this paper, including images, data, and a Mathematica notebook. (ZIP) [file pone.0125074.s002.zip › Supplementary file/images/PA05.png]

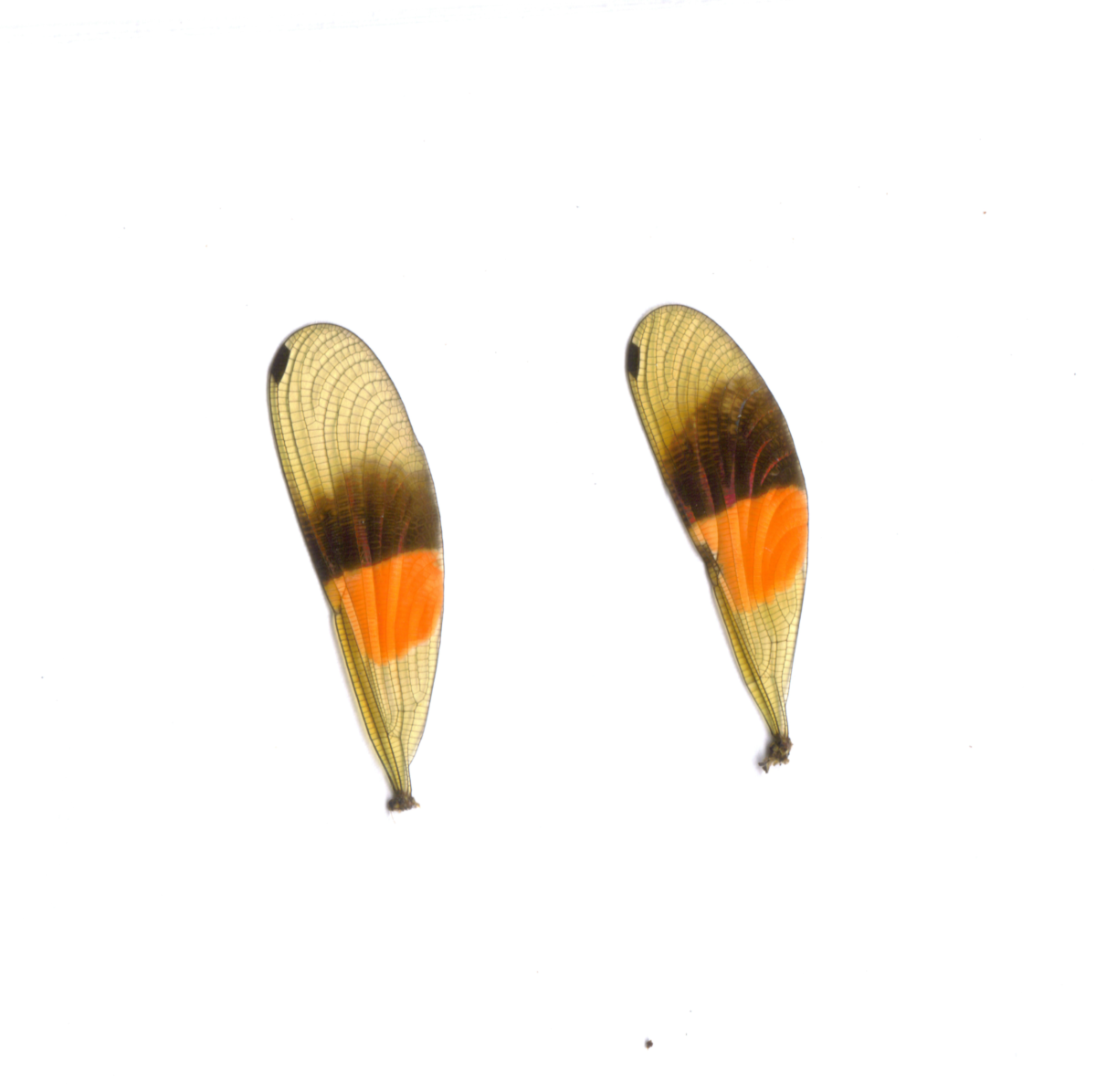

Supplement: S2 File — Compressed folder containing everything needed to run the analyses presented in this paper, including images, data, and a Mathematica notebook. (ZIP) [file pone.0125074.s002.zip › Supplementary file/images/PA87.png]

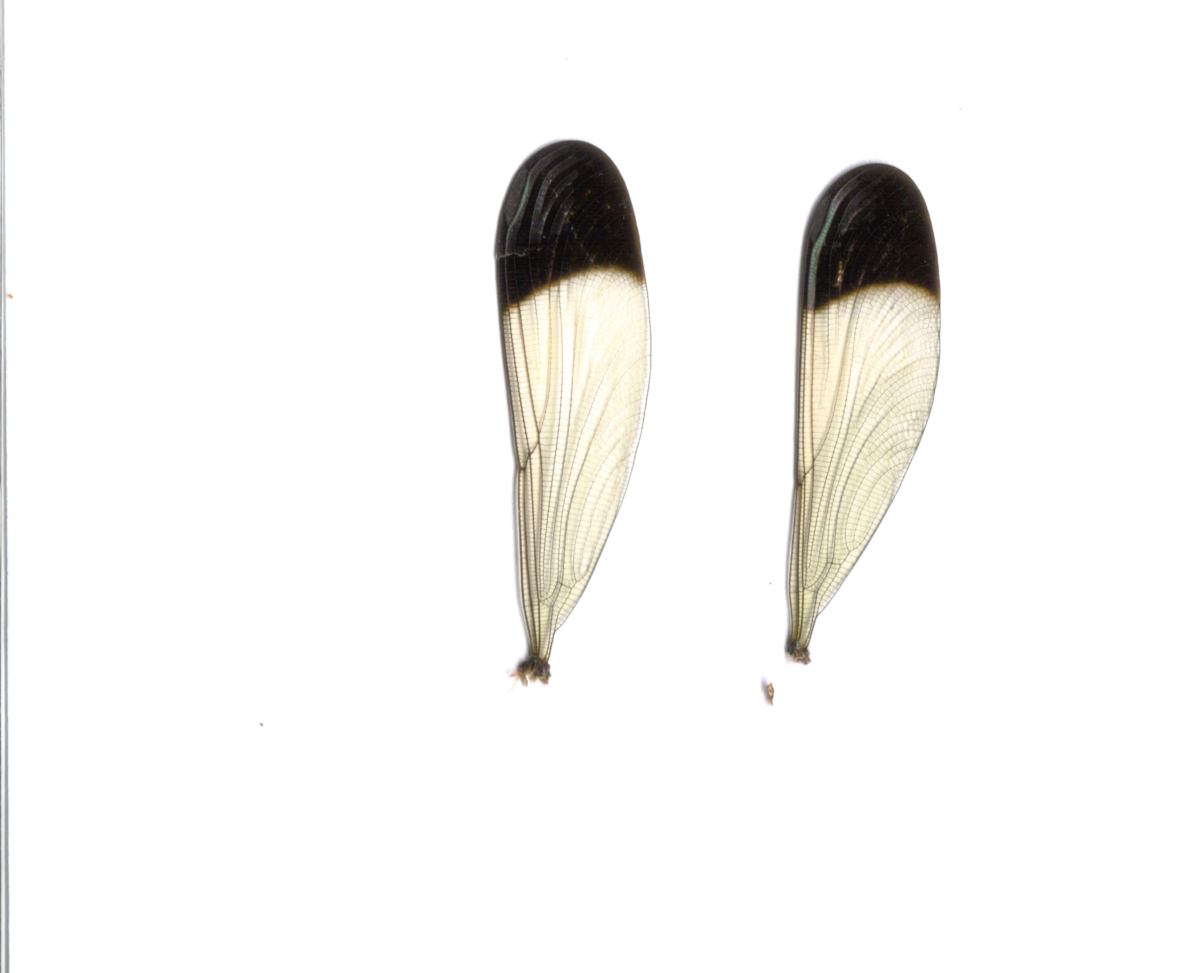

Supplement: S2 File — Compressed folder containing everything needed to run the analyses presented in this paper, including images, data, and a Mathematica notebook. (ZIP) [file pone.0125074.s002.zip › Supplementary file/images/PL10.png]

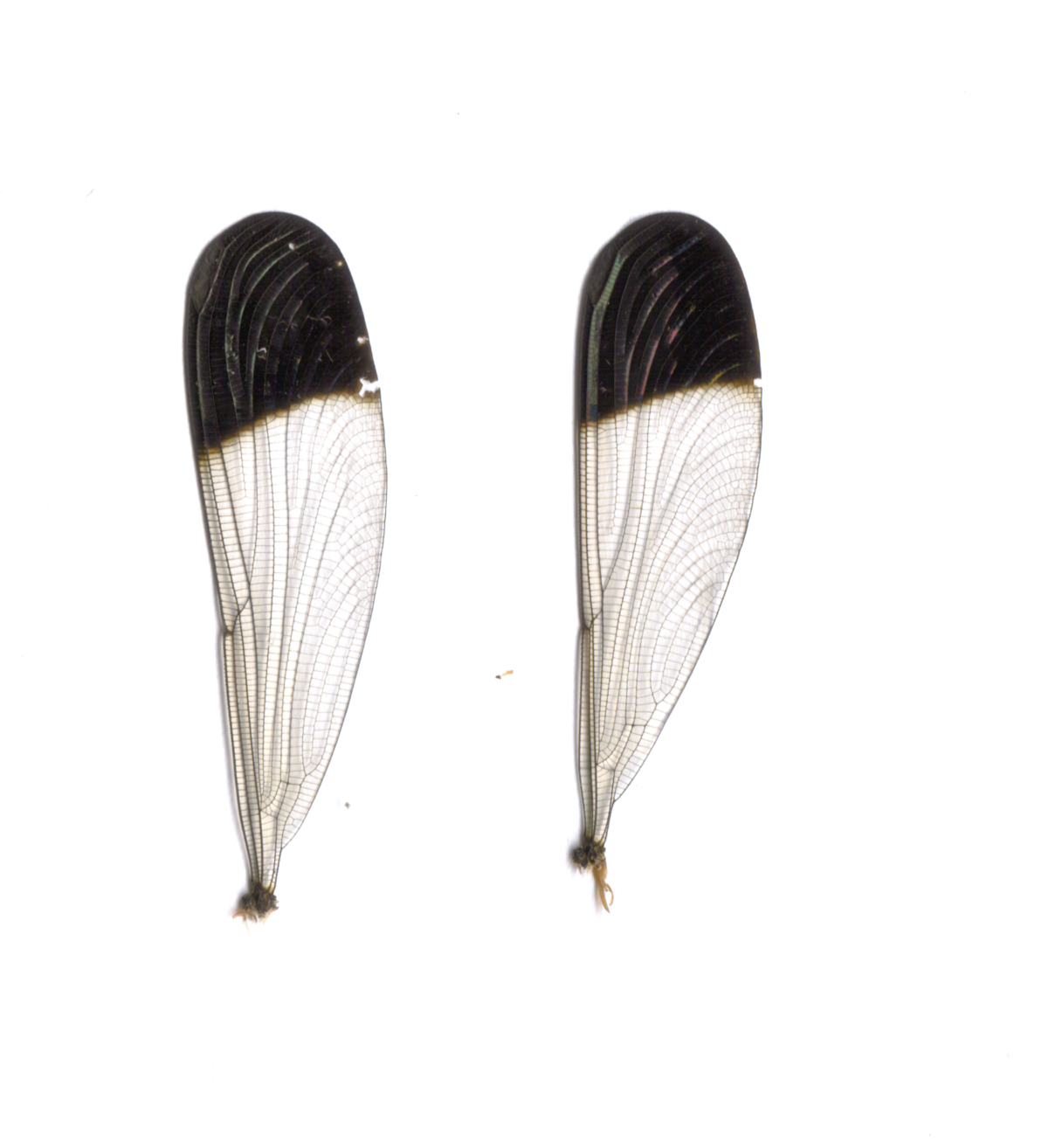

Supplement: S2 File — Compressed folder containing everything needed to run the analyses presented in this paper, including images, data, and a Mathematica notebook. (ZIP) [file pone.0125074.s002.zip › Supplementary file/images/PL35.png]

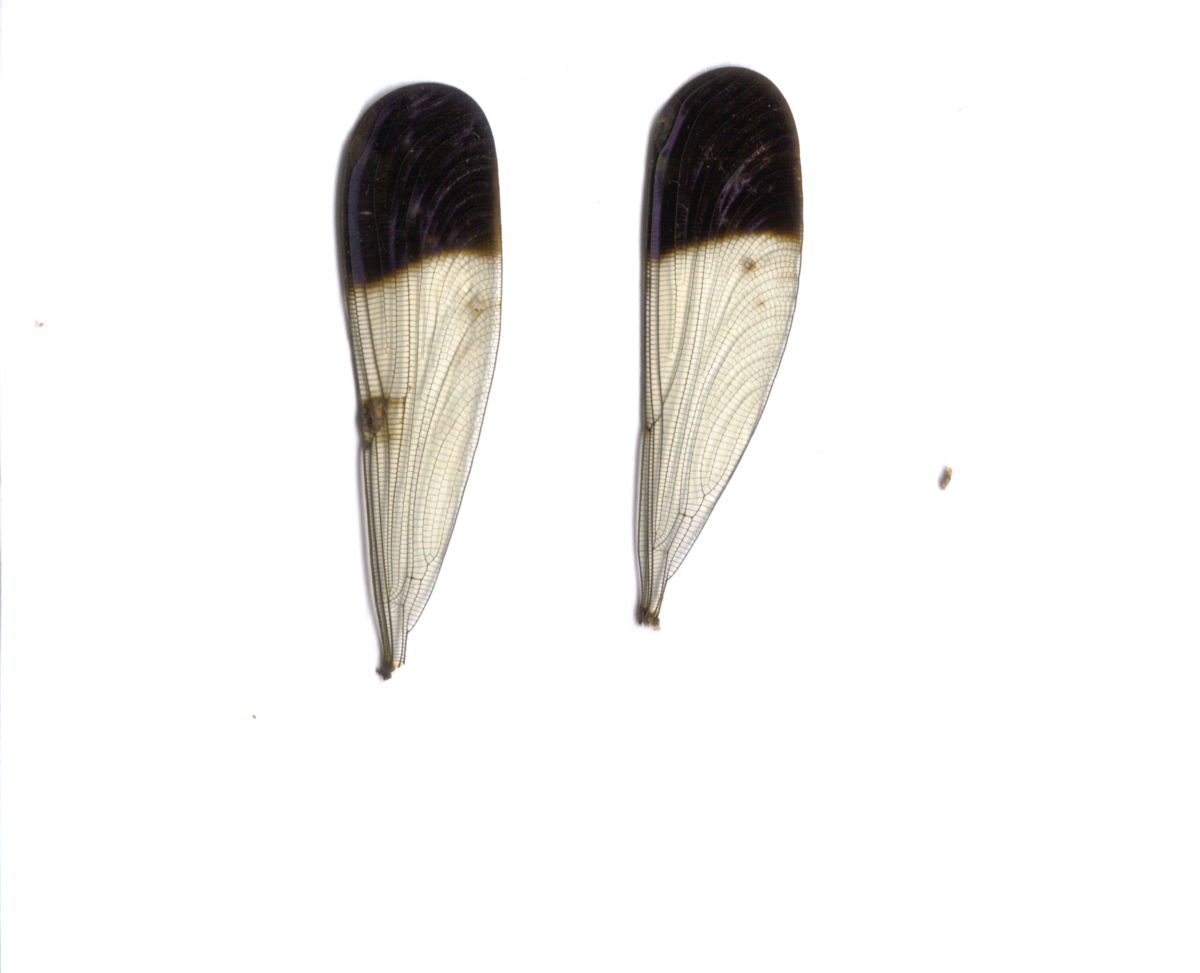

Supplement: S2 File — Compressed folder containing everything needed to run the analyses presented in this paper, including images, data, and a Mathematica notebook. (ZIP) [file pone.0125074.s002.zip › Supplementary file/images/PL37.png]

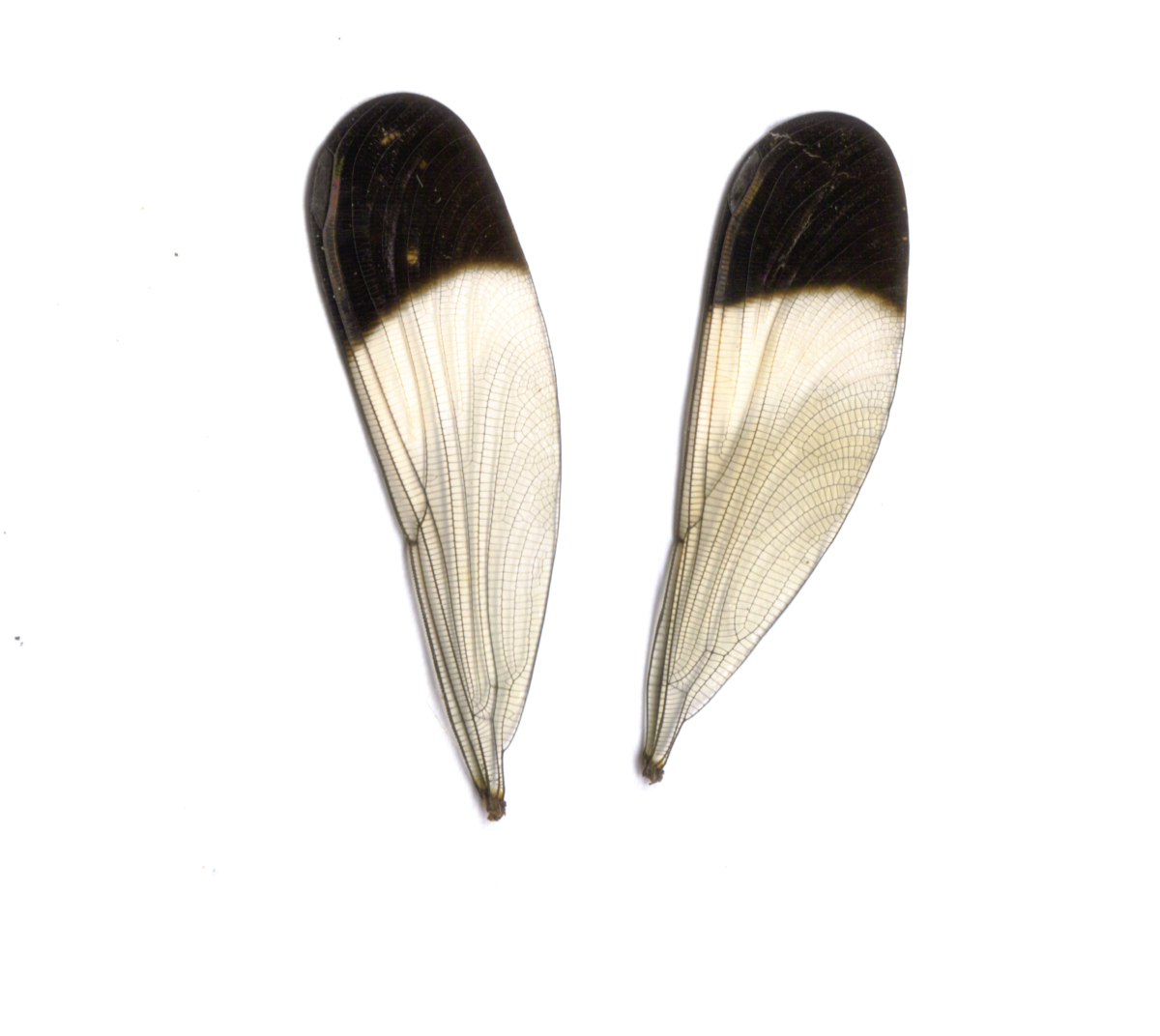

Supplement: S2 File — Compressed folder containing everything needed to run the analyses presented in this paper, including images, data, and a Mathematica notebook. (ZIP) [file pone.0125074.s002.zip › Supplementary file/images/PL39.png]

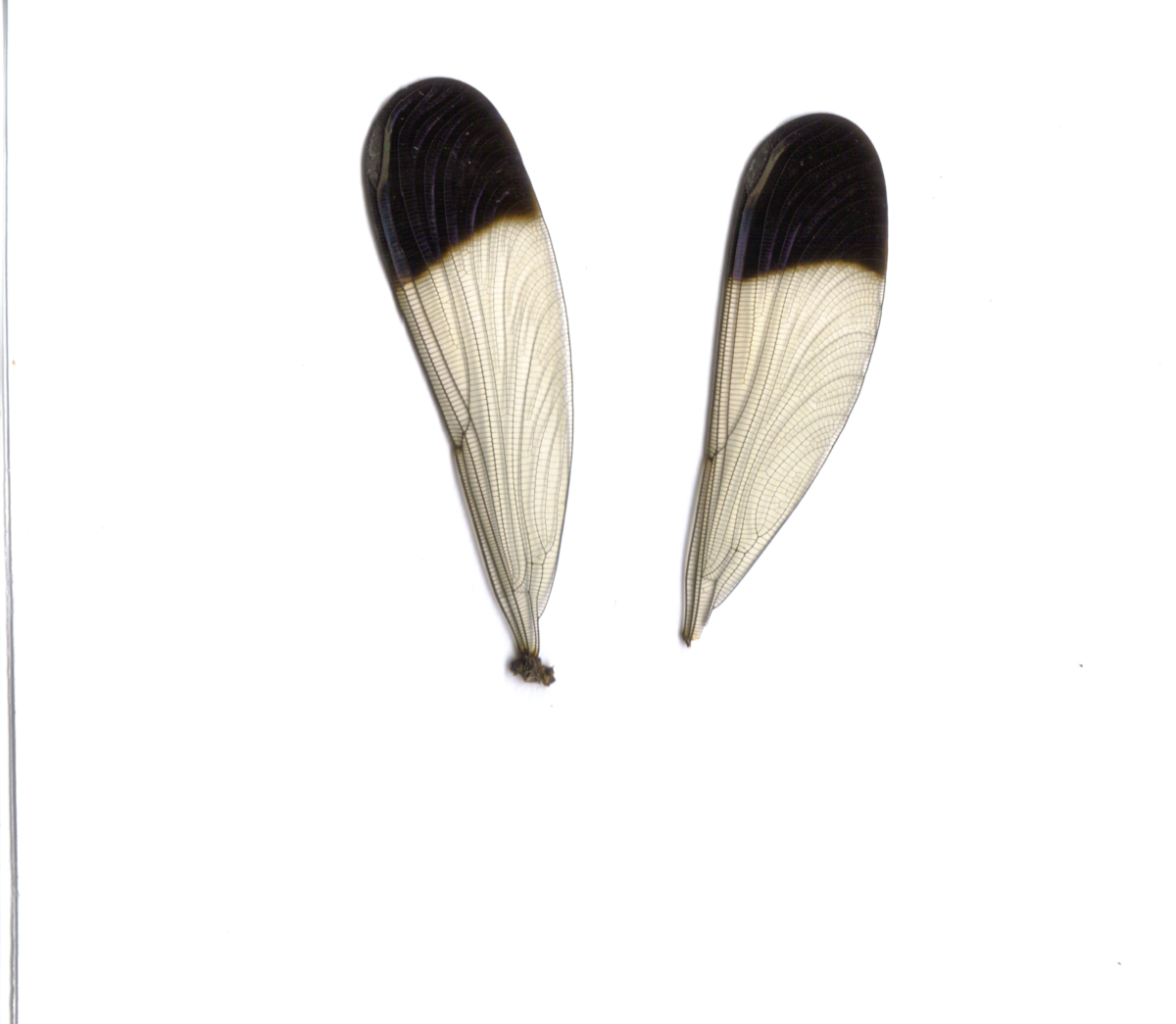

Supplement: S2 File — Compressed folder containing everything needed to run the analyses presented in this paper, including images, data, and a Mathematica notebook. (ZIP) [file pone.0125074.s002.zip › Supplementary file/images/PL39d.png]

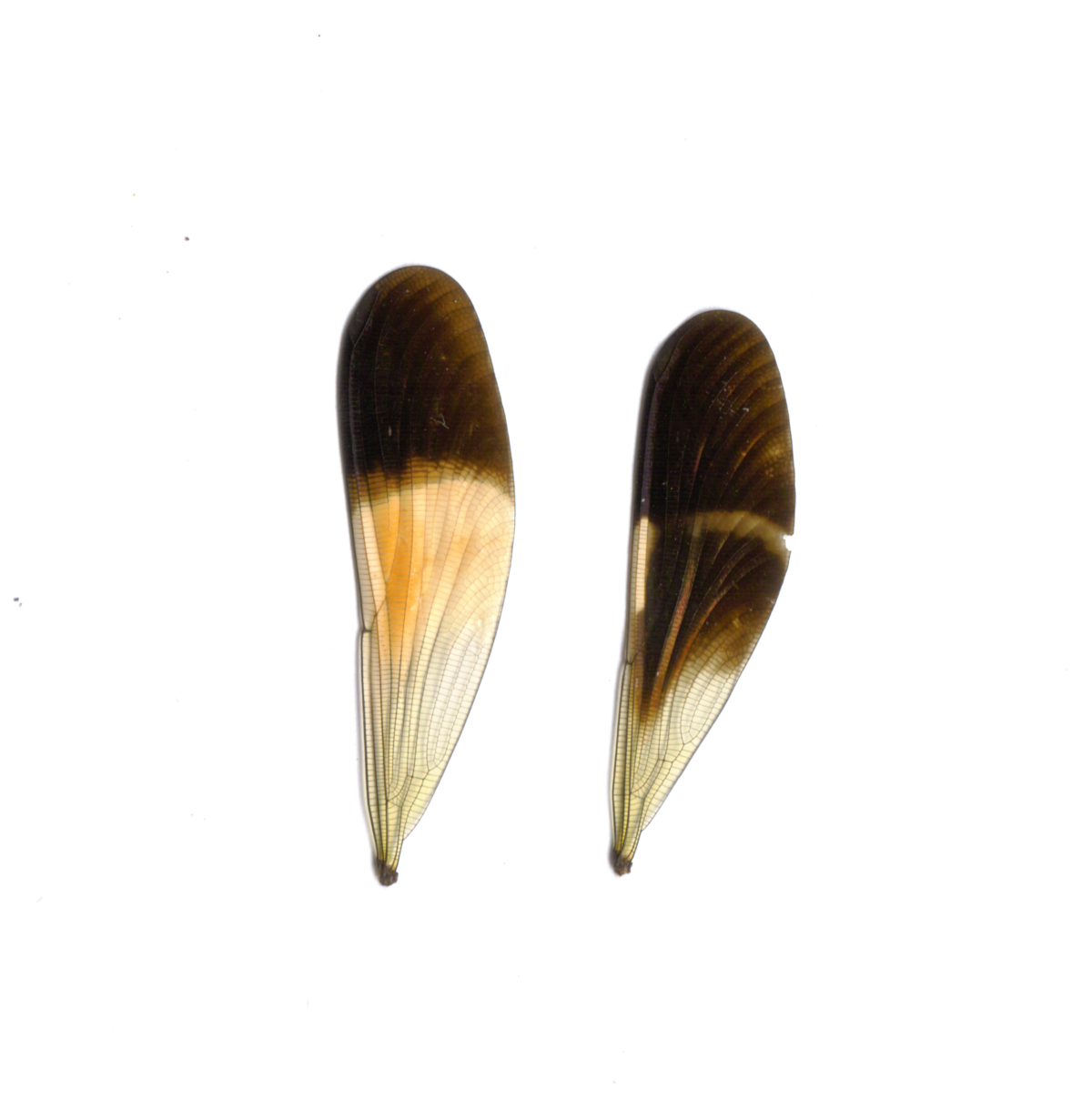

Supplement: S2 File — Compressed folder containing everything needed to run the analyses presented in this paper, including images, data, and a Mathematica notebook. (ZIP) [file pone.0125074.s002.zip › Supplementary file/images/PO20_A.png]

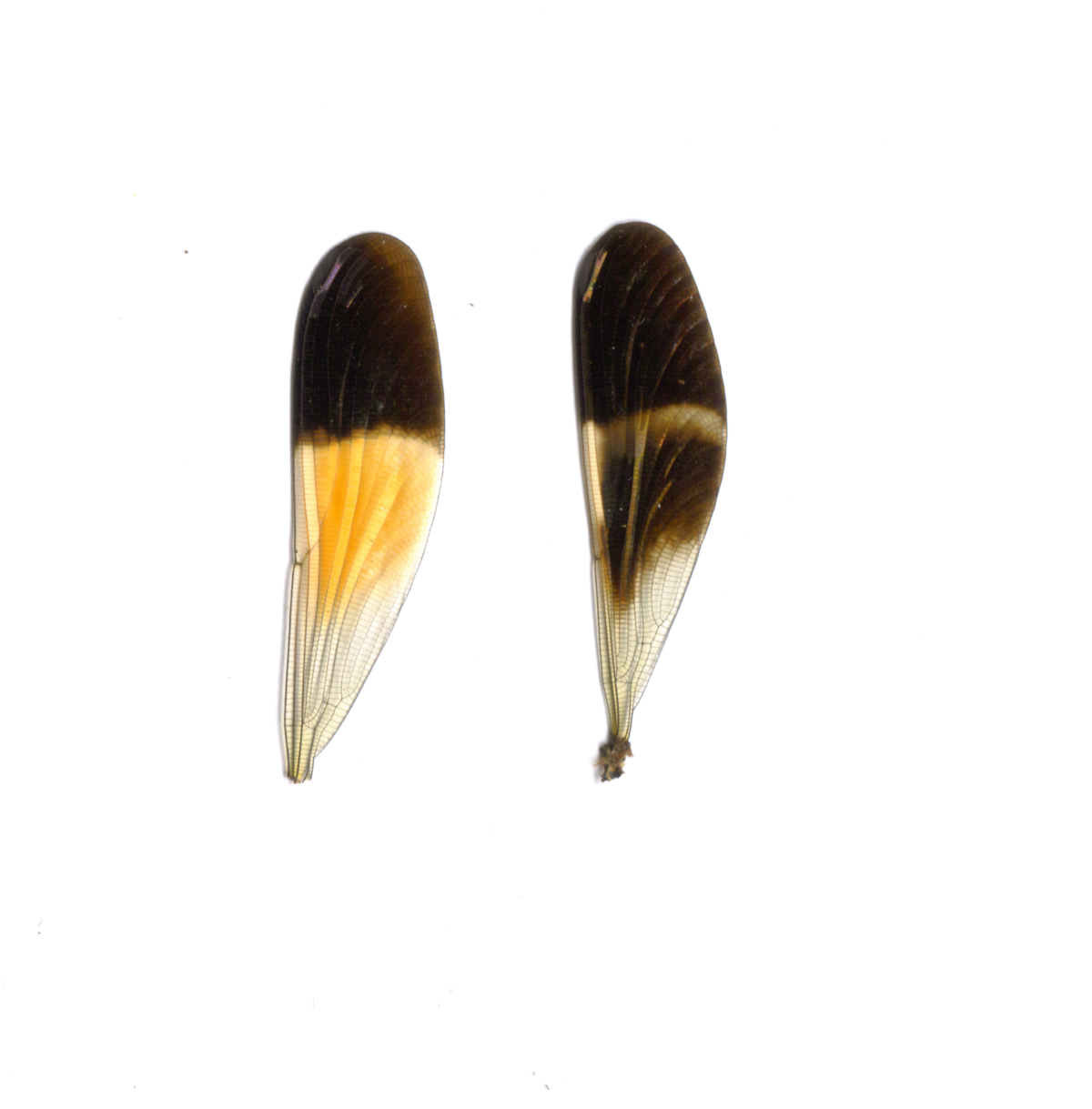

Supplement: S2 File — Compressed folder containing everything needed to run the analyses presented in this paper, including images, data, and a Mathematica notebook. (ZIP) [file pone.0125074.s002.zip › Supplementary file/images/PO20_B.png]

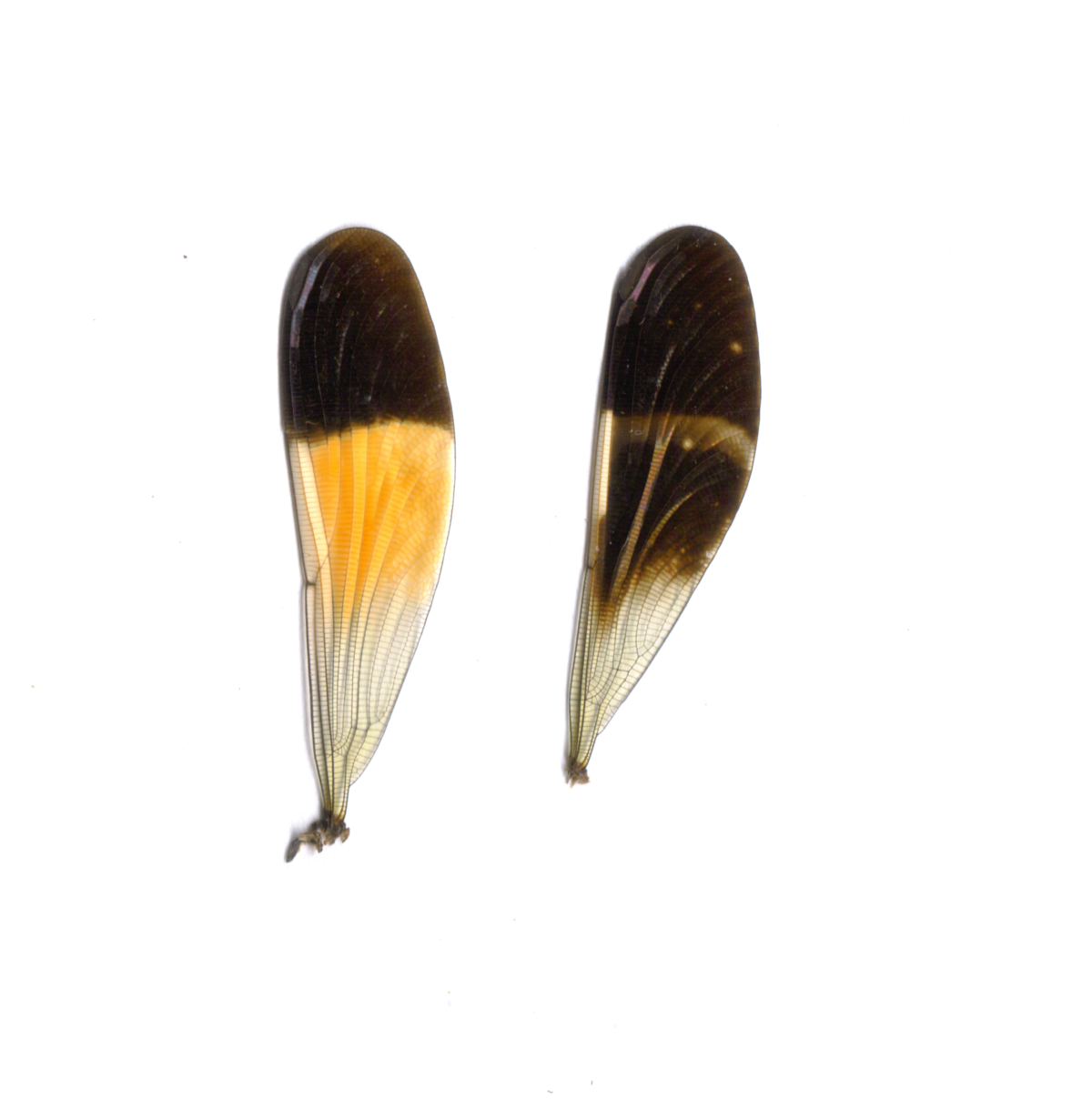

Supplement: S2 File — Compressed folder containing everything needed to run the analyses presented in this paper, including images, data, and a Mathematica notebook. (ZIP) [file pone.0125074.s002.zip › Supplementary file/images/PO20_C.png]

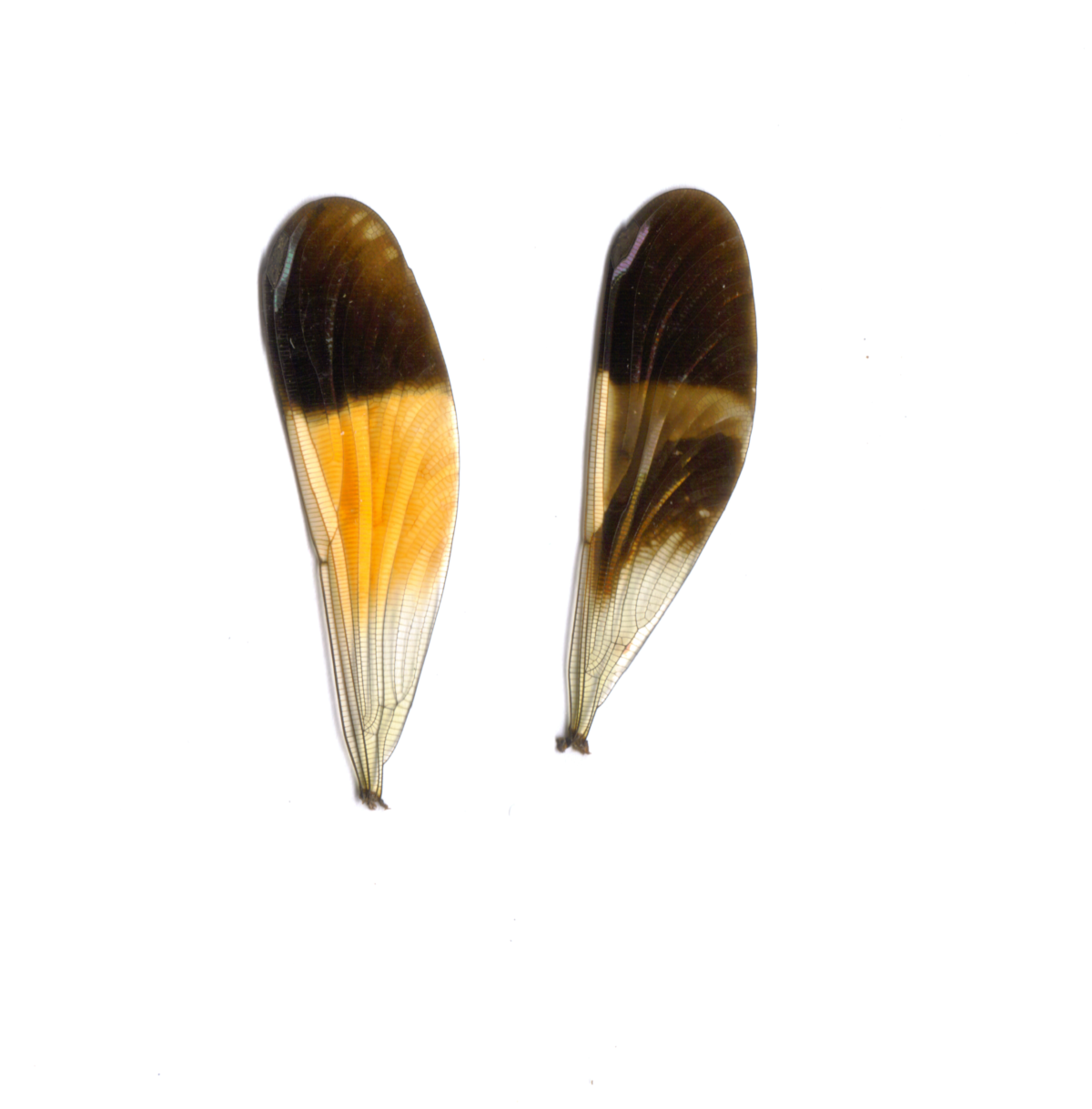

Supplement: S2 File — Compressed folder containing everything needed to run the analyses presented in this paper, including images, data, and a Mathematica notebook. (ZIP) [file pone.0125074.s002.zip › Supplementary file/images/PO30_A.png]

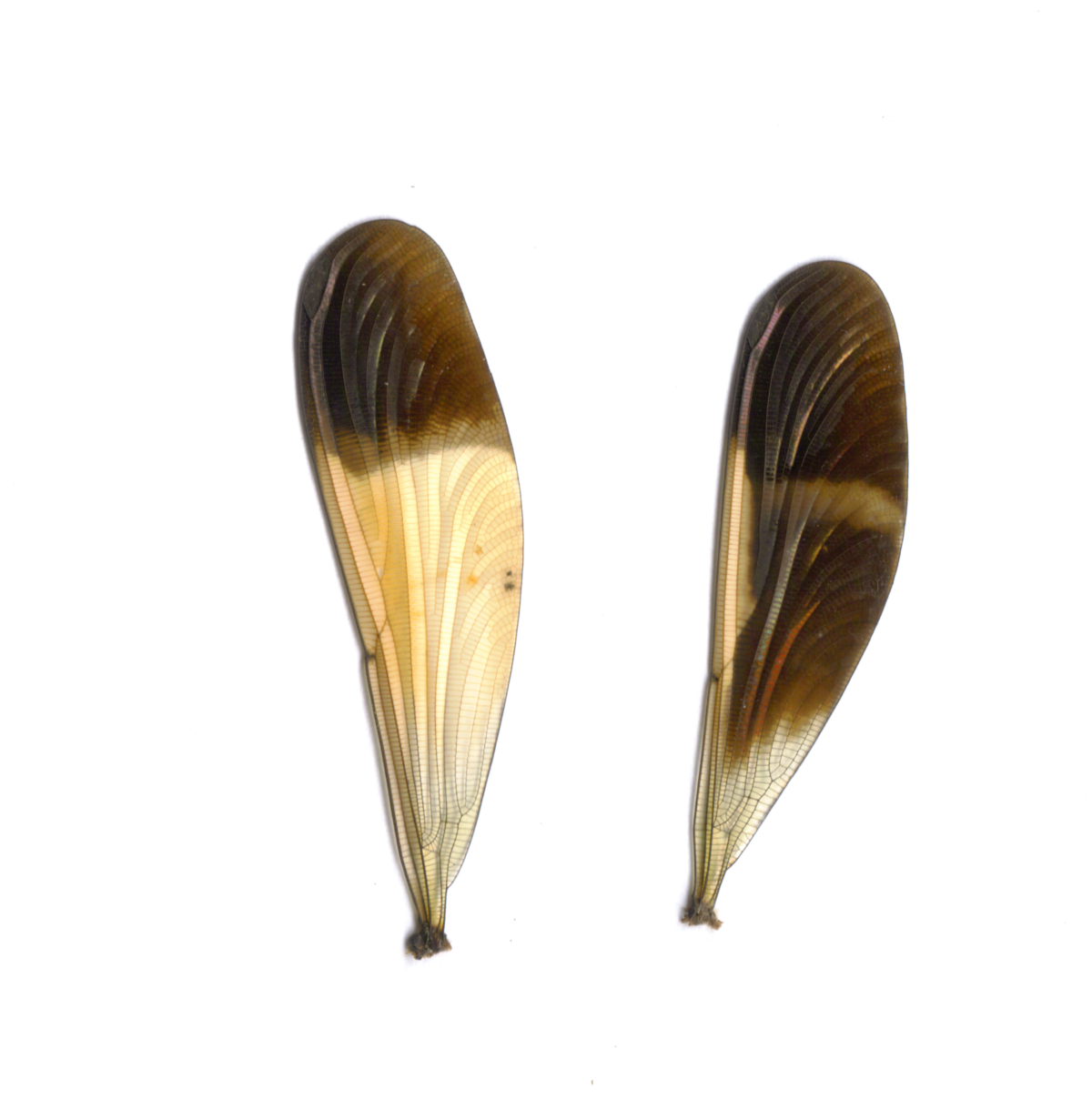

Supplement: S2 File — Compressed folder containing everything needed to run the analyses presented in this paper, including images, data, and a Mathematica notebook. (ZIP) [file pone.0125074.s002.zip › Supplementary file/images/PO30_B.png]

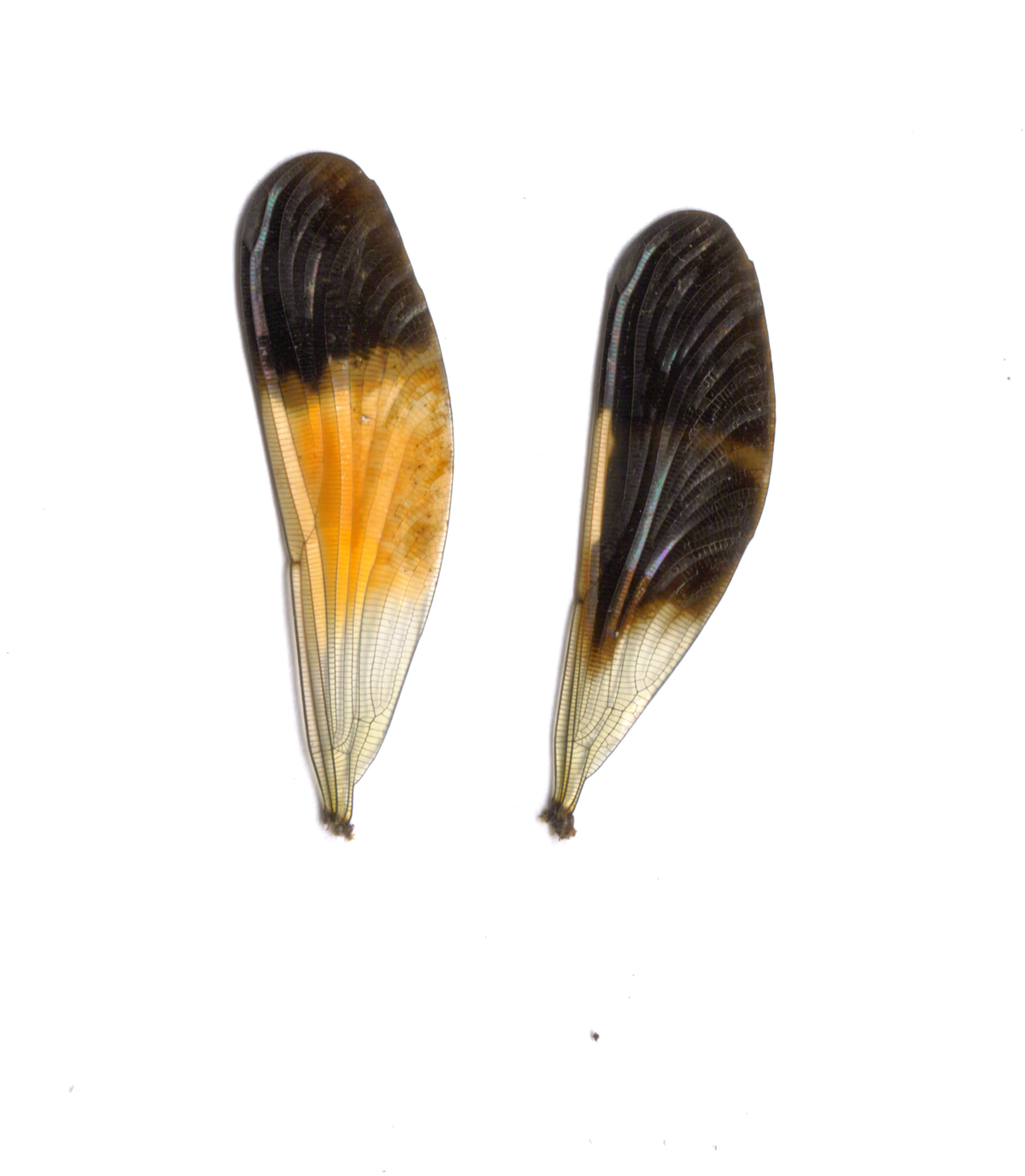

Supplement: S2 File — Compressed folder containing everything needed to run the analyses presented in this paper, including images, data, and a Mathematica notebook. (ZIP) [file pone.0125074.s002.zip › Supplementary file/images/PO30_C.png]

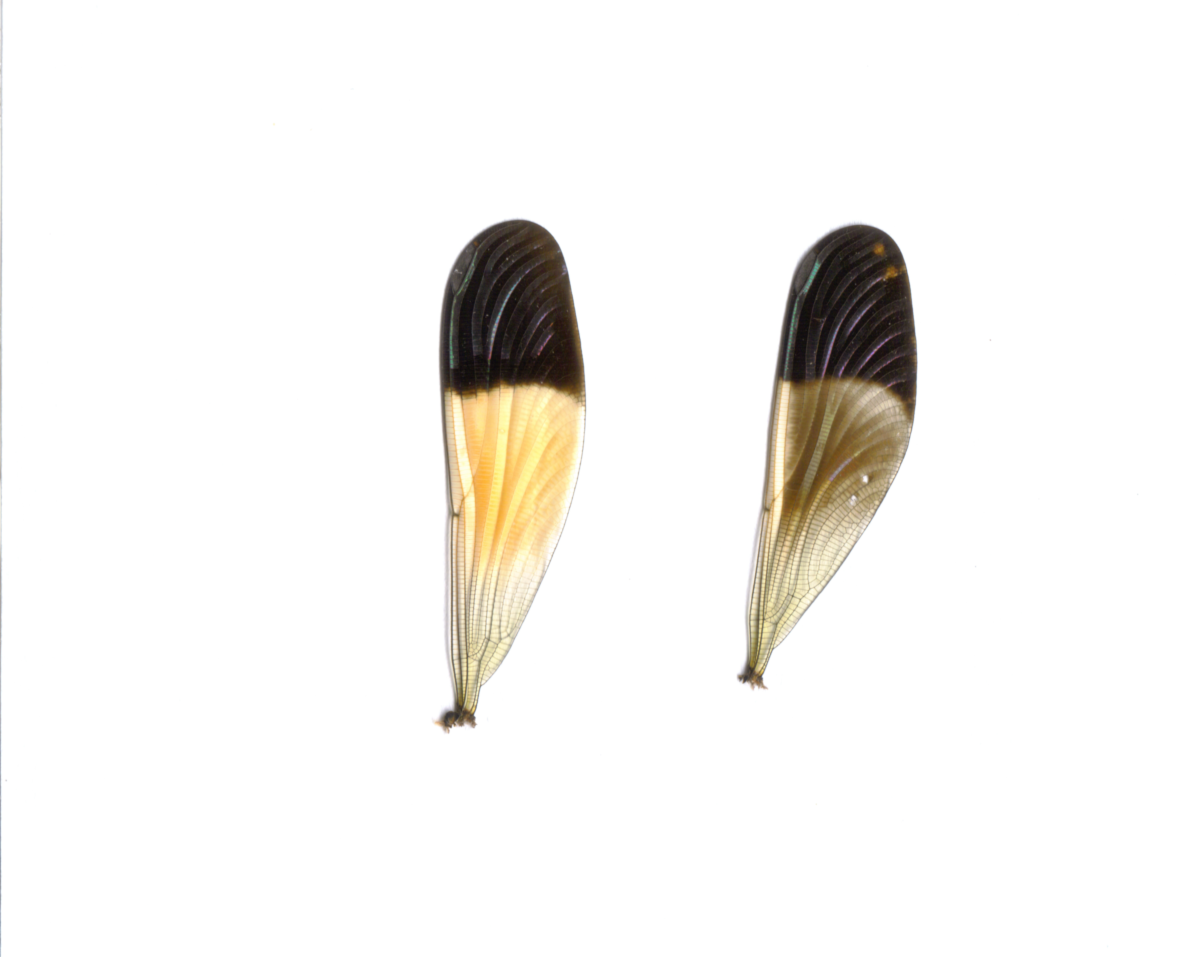

Supplement: S2 File — Compressed folder containing everything needed to run the analyses presented in this paper, including images, data, and a Mathematica notebook. (ZIP) [file pone.0125074.s002.zip › Supplementary file/images/PO42.png]

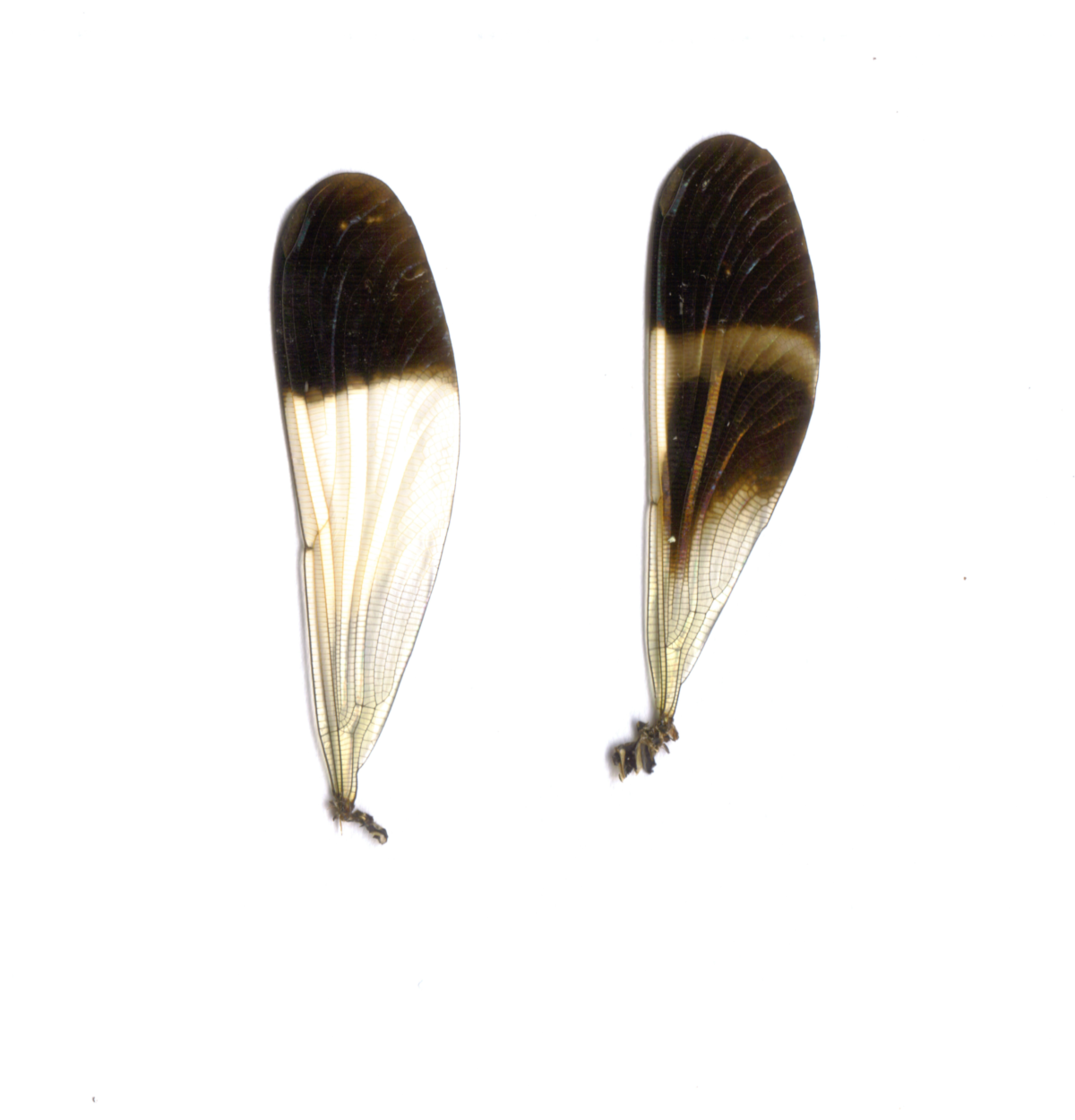

Supplement: S2 File — Compressed folder containing everything needed to run the analyses presented in this paper, including images, data, and a Mathematica notebook. (ZIP) [file pone.0125074.s002.zip › Supplementary file/images/PO43.png]

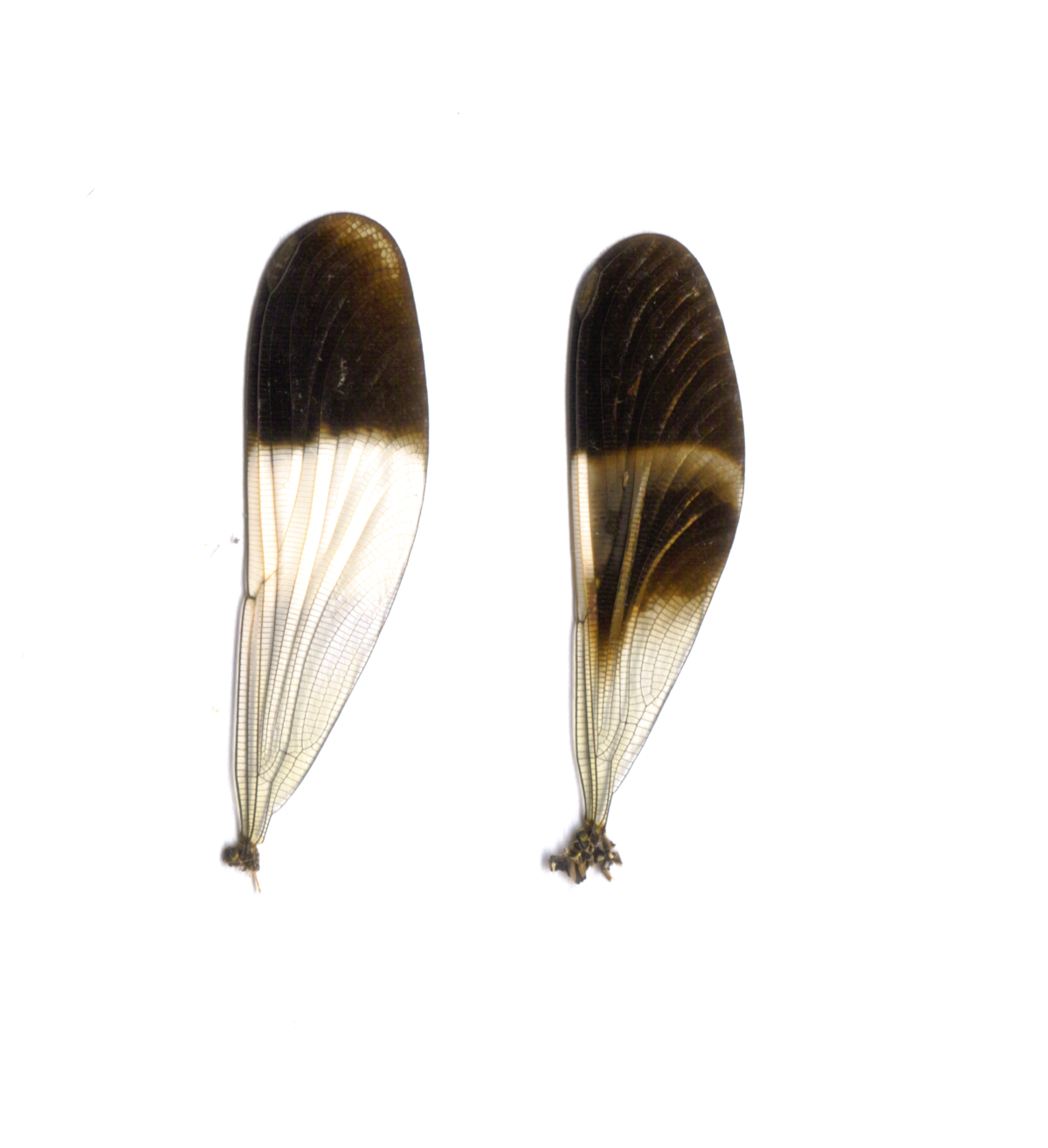

Supplement: S2 File — Compressed folder containing everything needed to run the analyses presented in this paper, including images, data, and a Mathematica notebook. (ZIP) [file pone.0125074.s002.zip › Supplementary file/images/PO45.png]

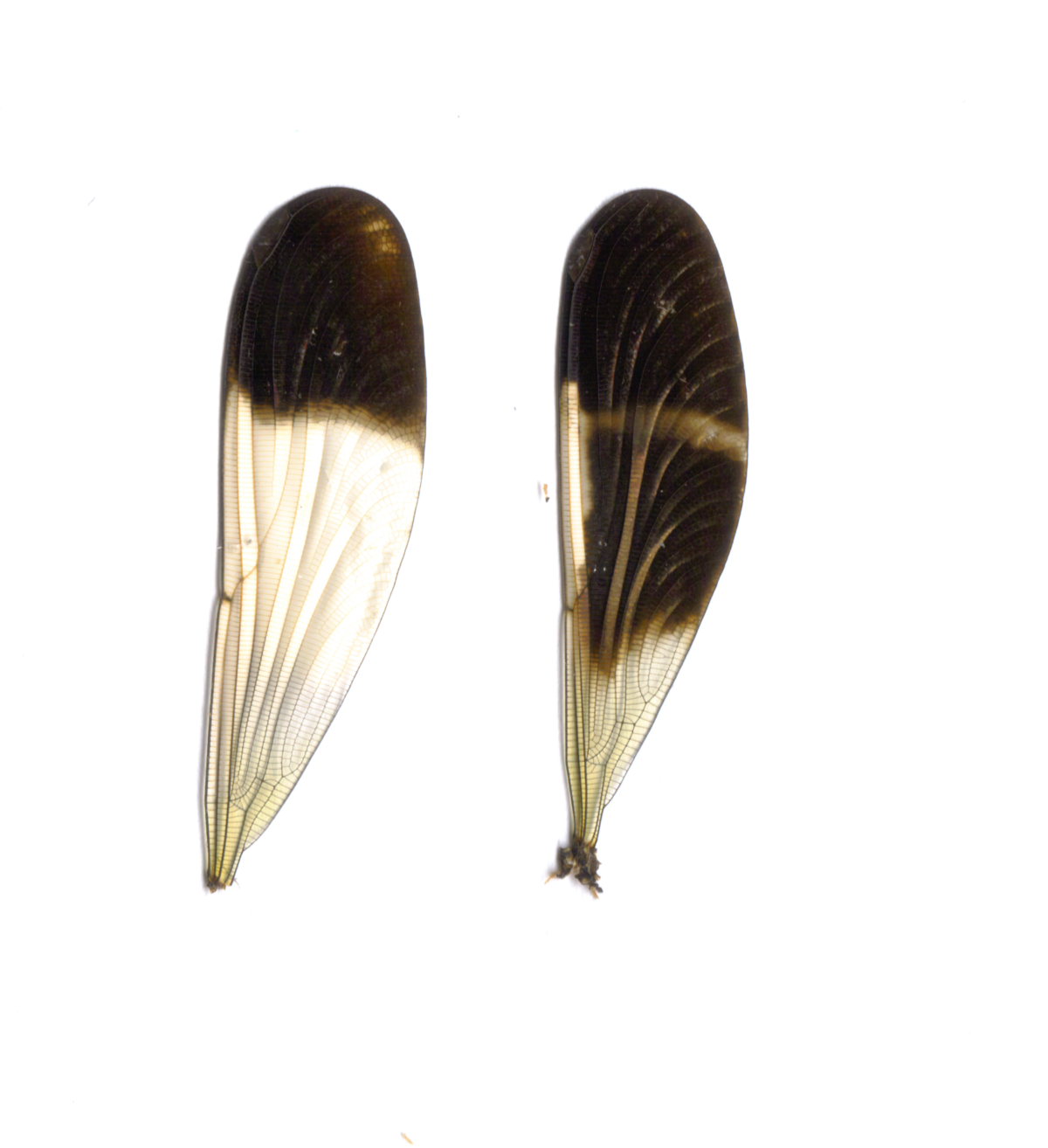

Supplement: S2 File — Compressed folder containing everything needed to run the analyses presented in this paper, including images, data, and a Mathematica notebook. (ZIP) [file pone.0125074.s002.zip › Supplementary file/images/PO47.png]

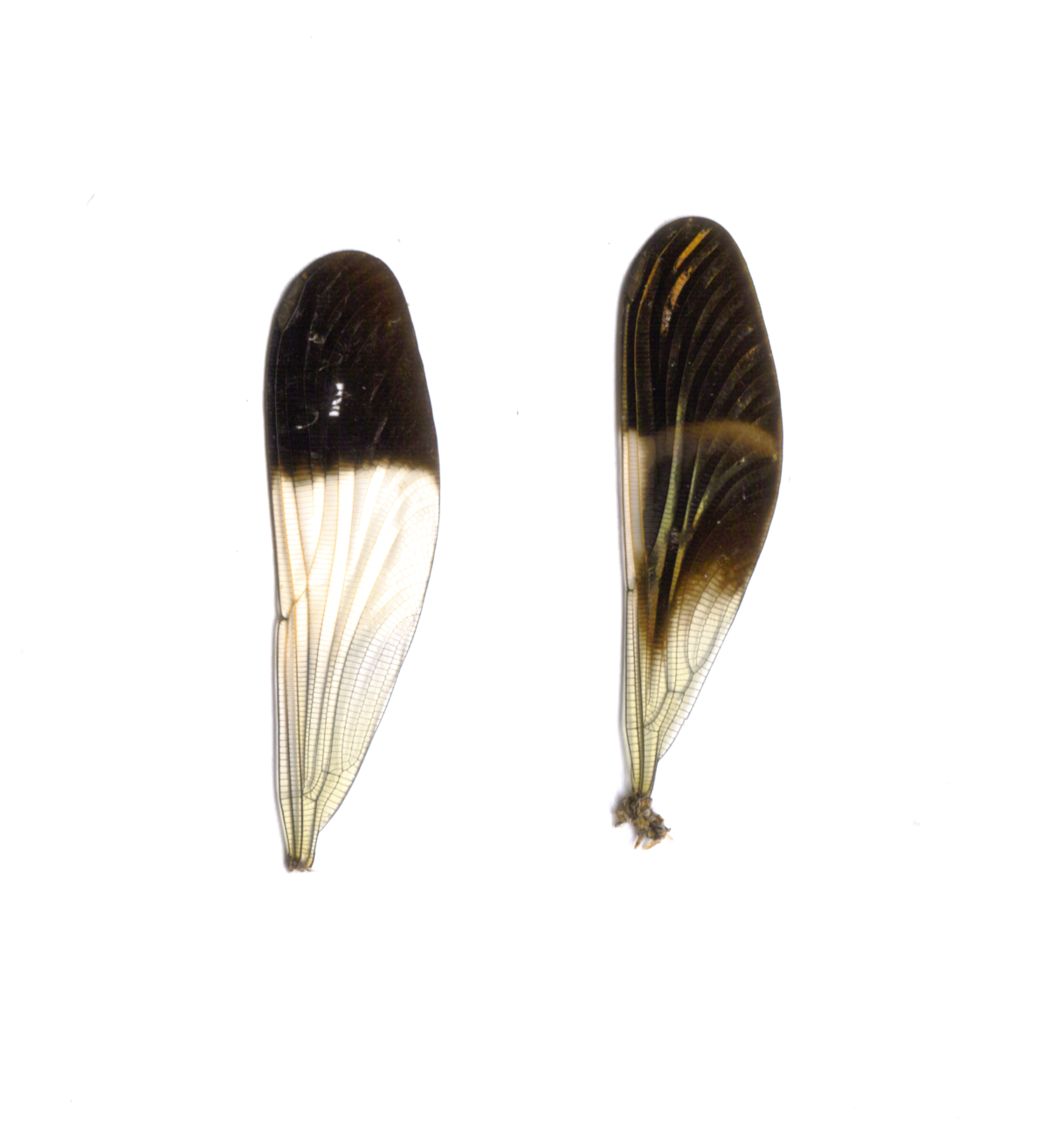

Supplement: S2 File — Compressed folder containing everything needed to run the analyses presented in this paper, including images, data, and a Mathematica notebook. (ZIP) [file pone.0125074.s002.zip › Supplementary file/images/PO47b.png]

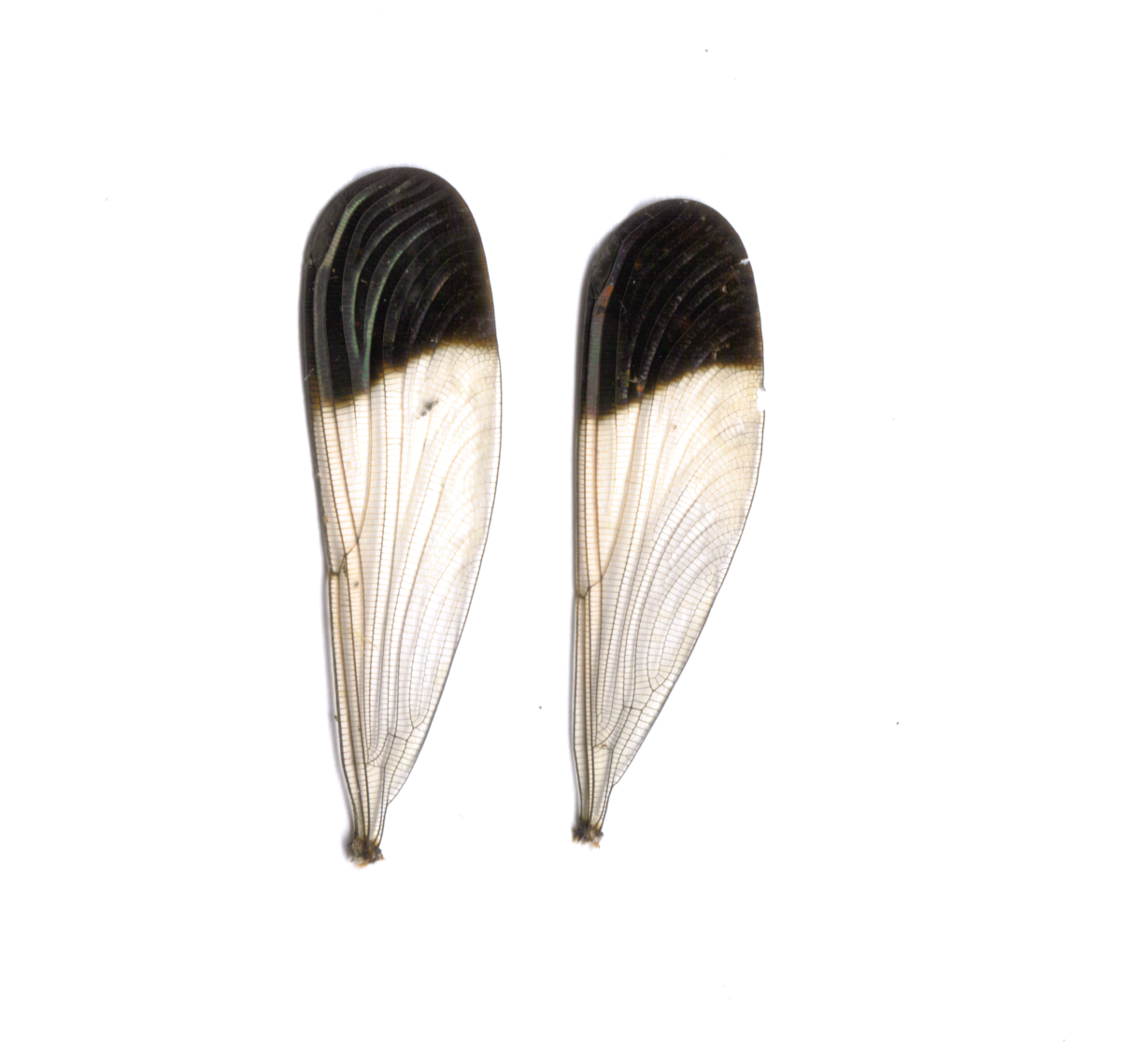

Supplement: S2 File — Compressed folder containing everything needed to run the analyses presented in this paper, including images, data, and a Mathematica notebook. (ZIP) [file pone.0125074.s002.zip › Supplementary file/images/PP23.png]

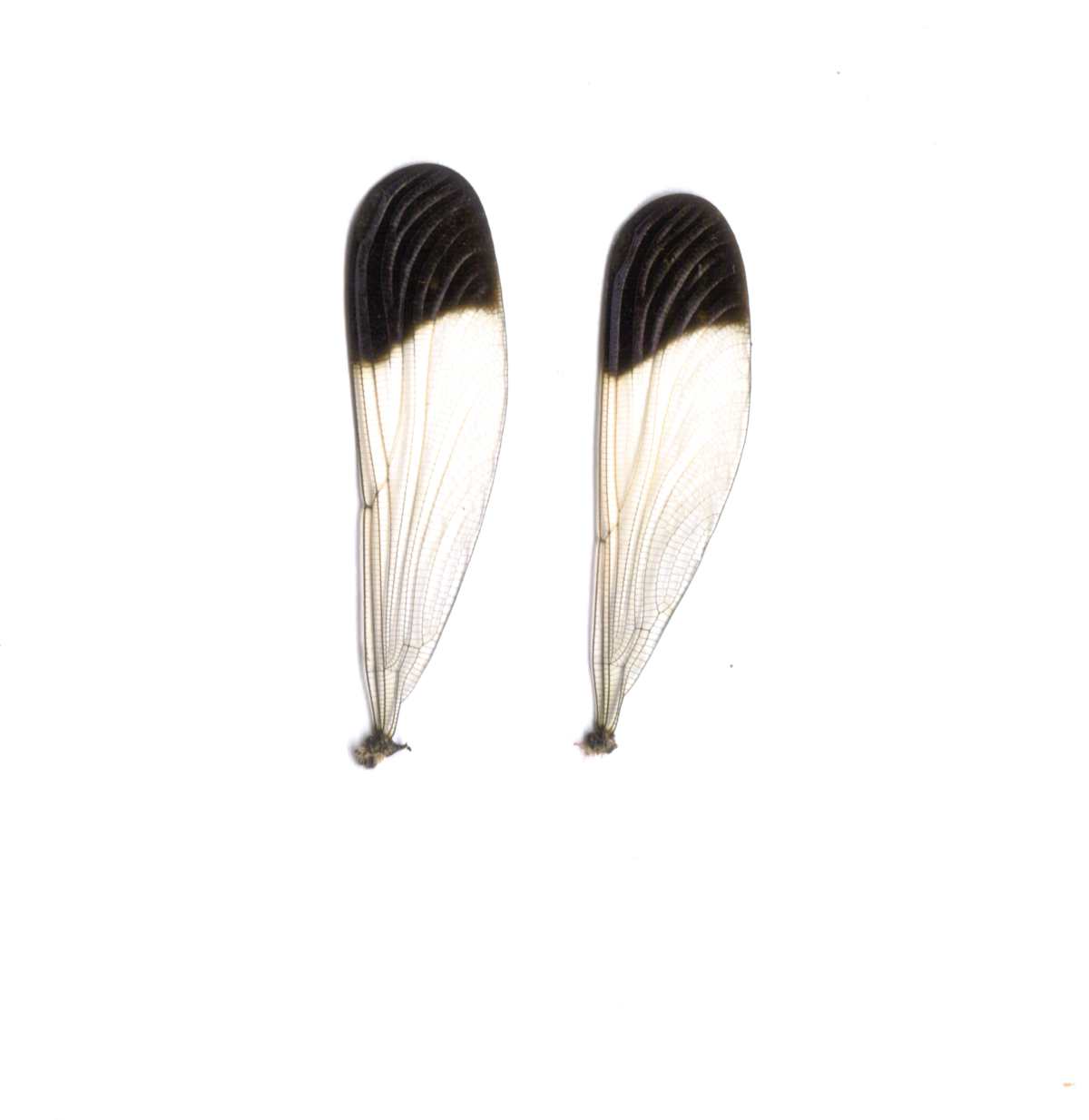

Supplement: S2 File — Compressed folder containing everything needed to run the analyses presented in this paper, including images, data, and a Mathematica notebook. (ZIP) [file pone.0125074.s002.zip › Supplementary file/images/PP24.png]

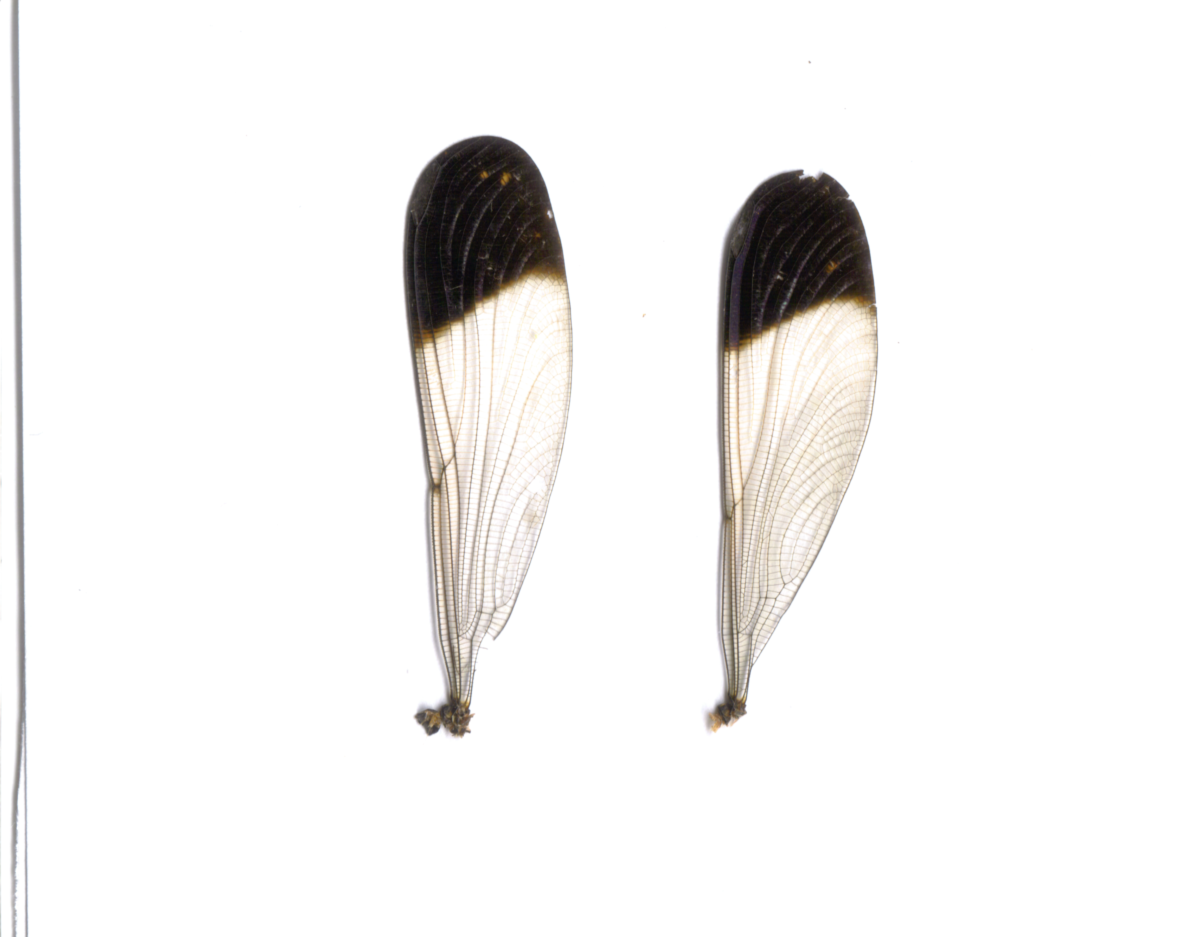

Supplement: S2 File — Compressed folder containing everything needed to run the analyses presented in this paper, including images, data, and a Mathematica notebook. (ZIP) [file pone.0125074.s002.zip › Supplementary file/images/PP26.png]

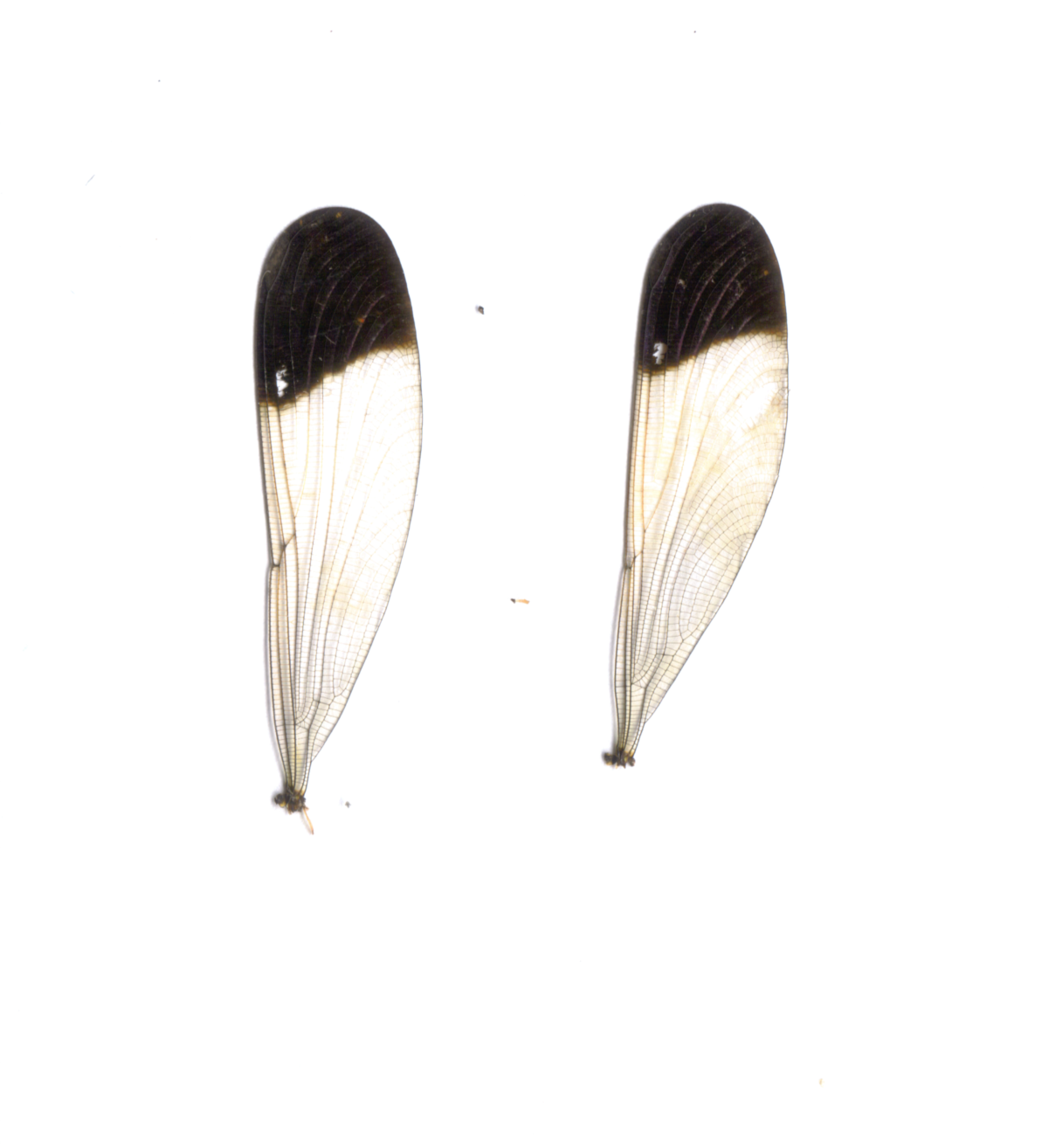

Supplement: S2 File — Compressed folder containing everything needed to run the analyses presented in this paper, including images, data, and a Mathematica notebook. (ZIP) [file pone.0125074.s002.zip › Supplementary file/images/PP28.png]

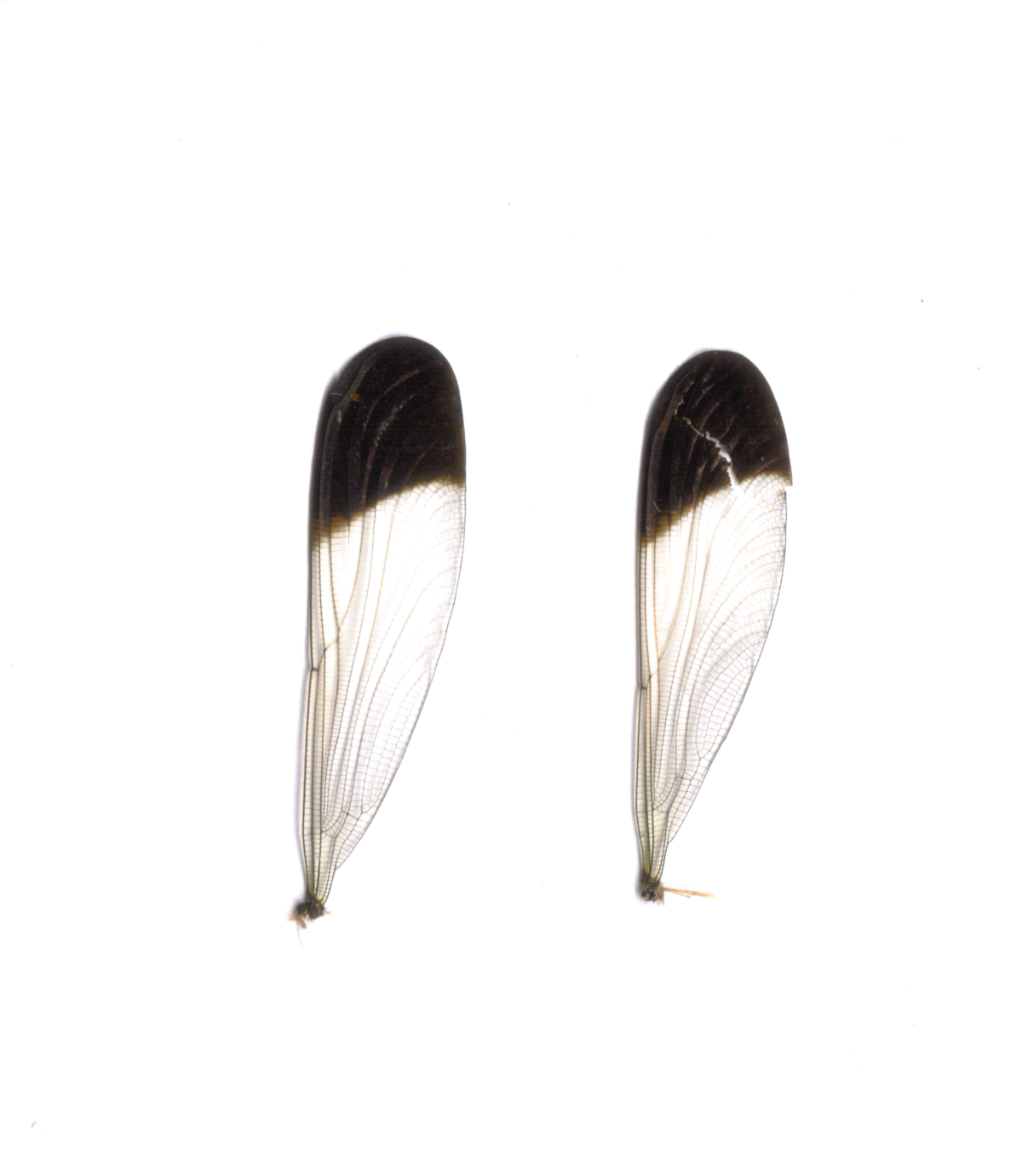

Supplement: S2 File — Compressed folder containing everything needed to run the analyses presented in this paper, including images, data, and a Mathematica notebook. (ZIP) [file pone.0125074.s002.zip › Supplementary file/images/PP29.png]

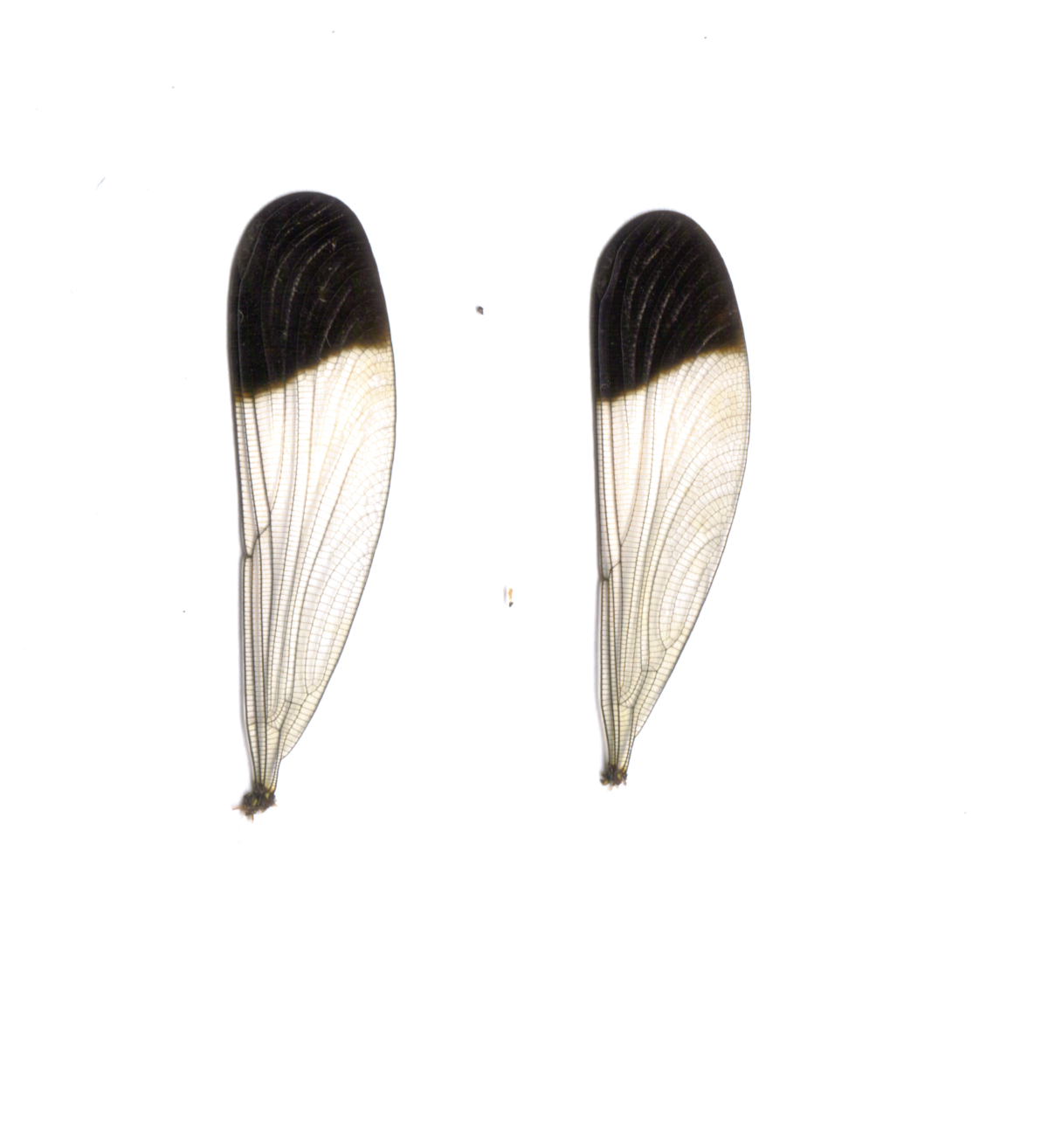

Supplement: S2 File — Compressed folder containing everything needed to run the analyses presented in this paper, including images, data, and a Mathematica notebook. (ZIP) [file pone.0125074.s002.zip › Supplementary file/images/PP30.png]

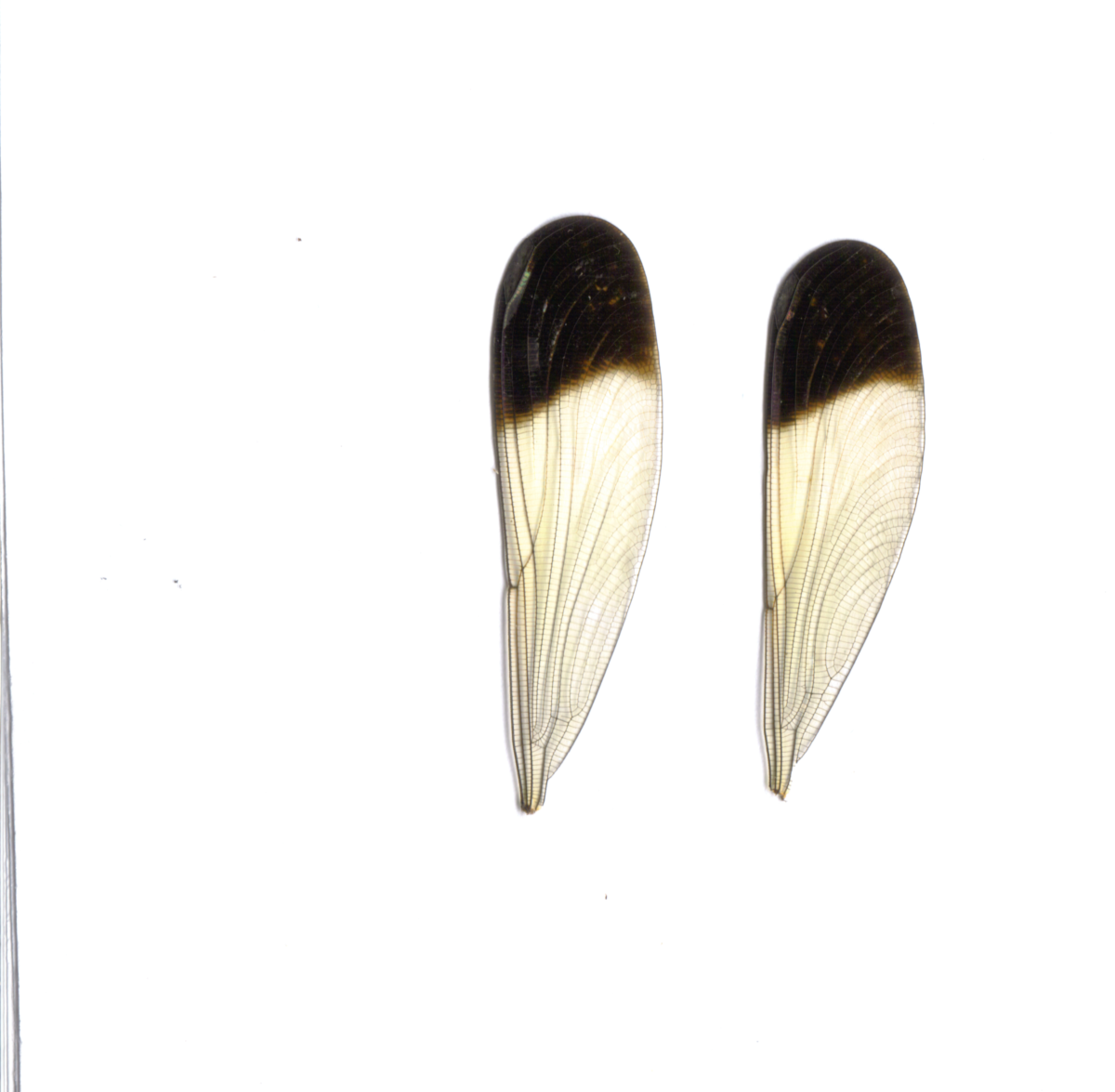

Supplement: S2 File — Compressed folder containing everything needed to run the analyses presented in this paper, including images, data, and a Mathematica notebook. (ZIP) [file pone.0125074.s002.zip › Supplementary file/images/PP67.png]

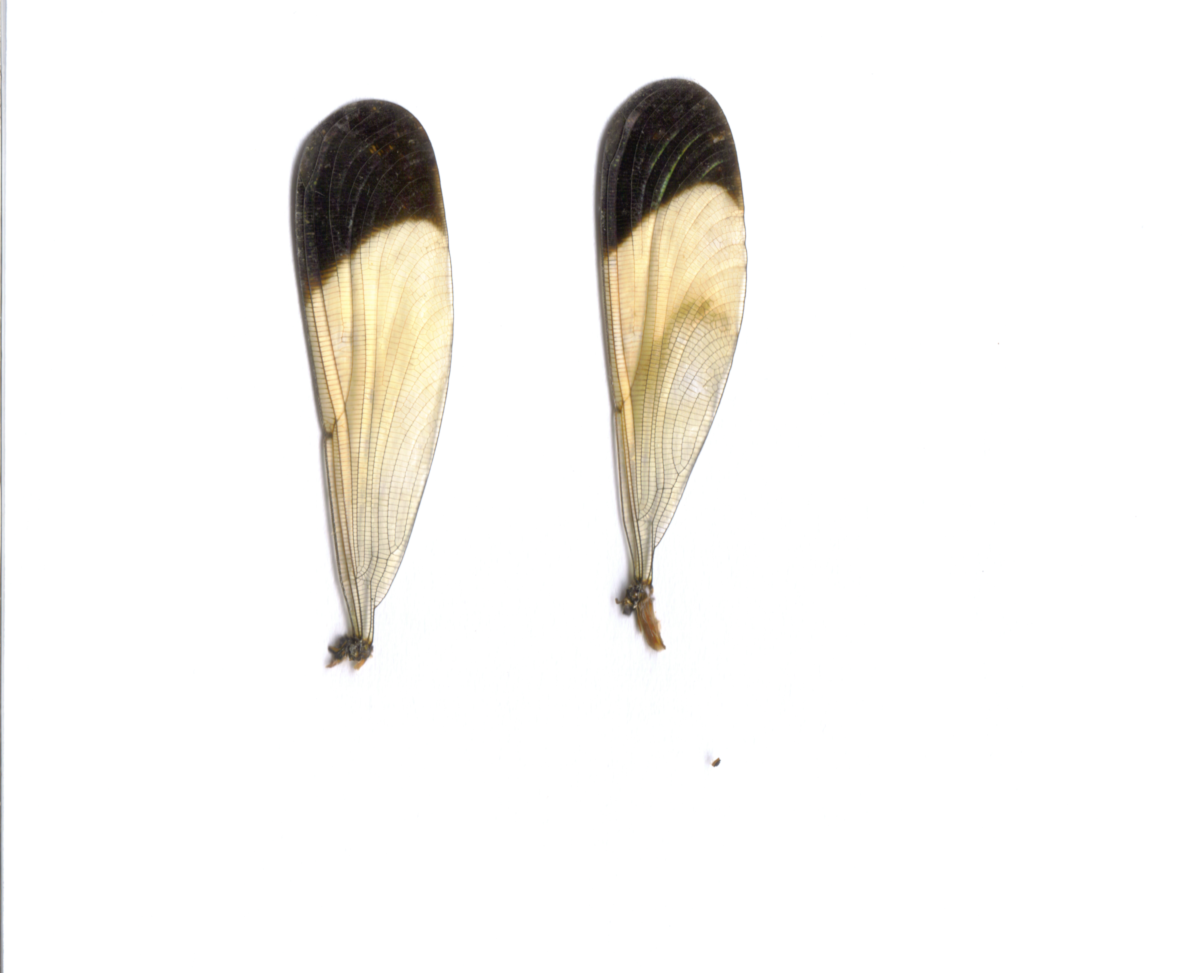

Supplement: S2 File — Compressed folder containing everything needed to run the analyses presented in this paper, including images, data, and a Mathematica notebook. (ZIP) [file pone.0125074.s002.zip › Supplementary file/images/PP68.png]

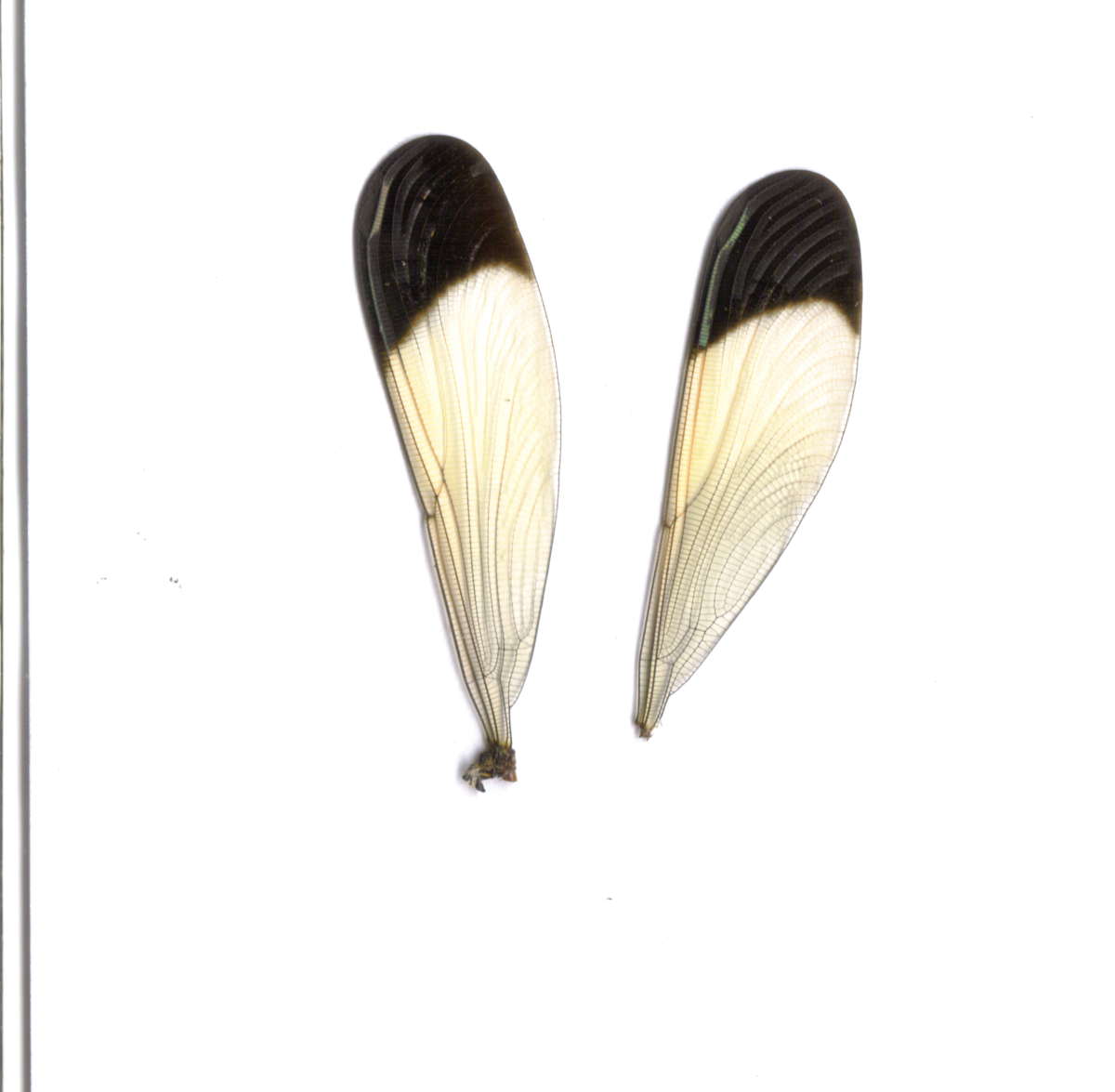

Supplement: S2 File — Compressed folder containing everything needed to run the analyses presented in this paper, including images, data, and a Mathematica notebook. (ZIP) [file pone.0125074.s002.zip › Supplementary file/images/PP69.png]

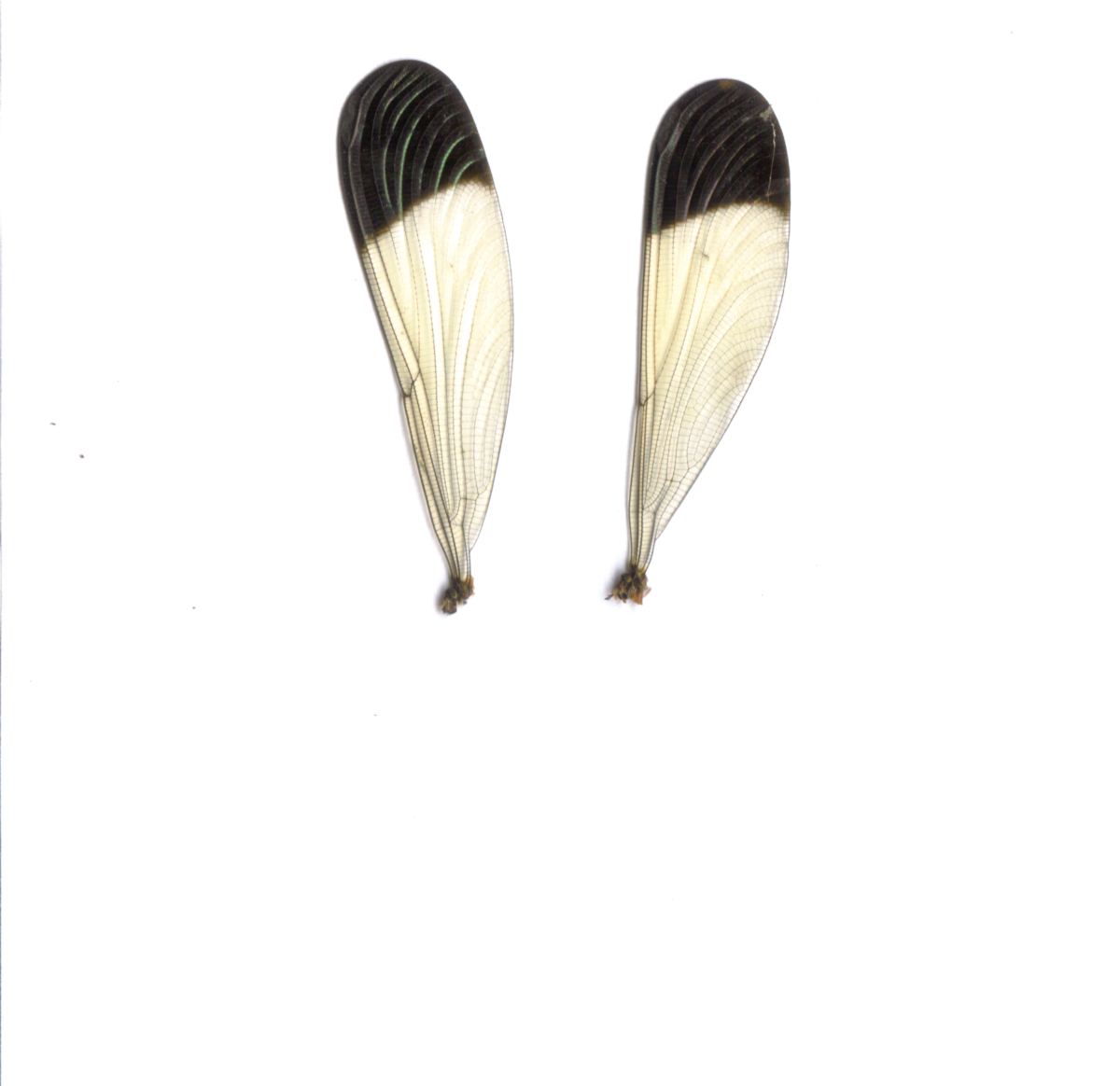

Supplement: S2 File — Compressed folder containing everything needed to run the analyses presented in this paper, including images, data, and a Mathematica notebook. (ZIP) [file pone.0125074.s002.zip › Supplementary file/images/PP77.png]

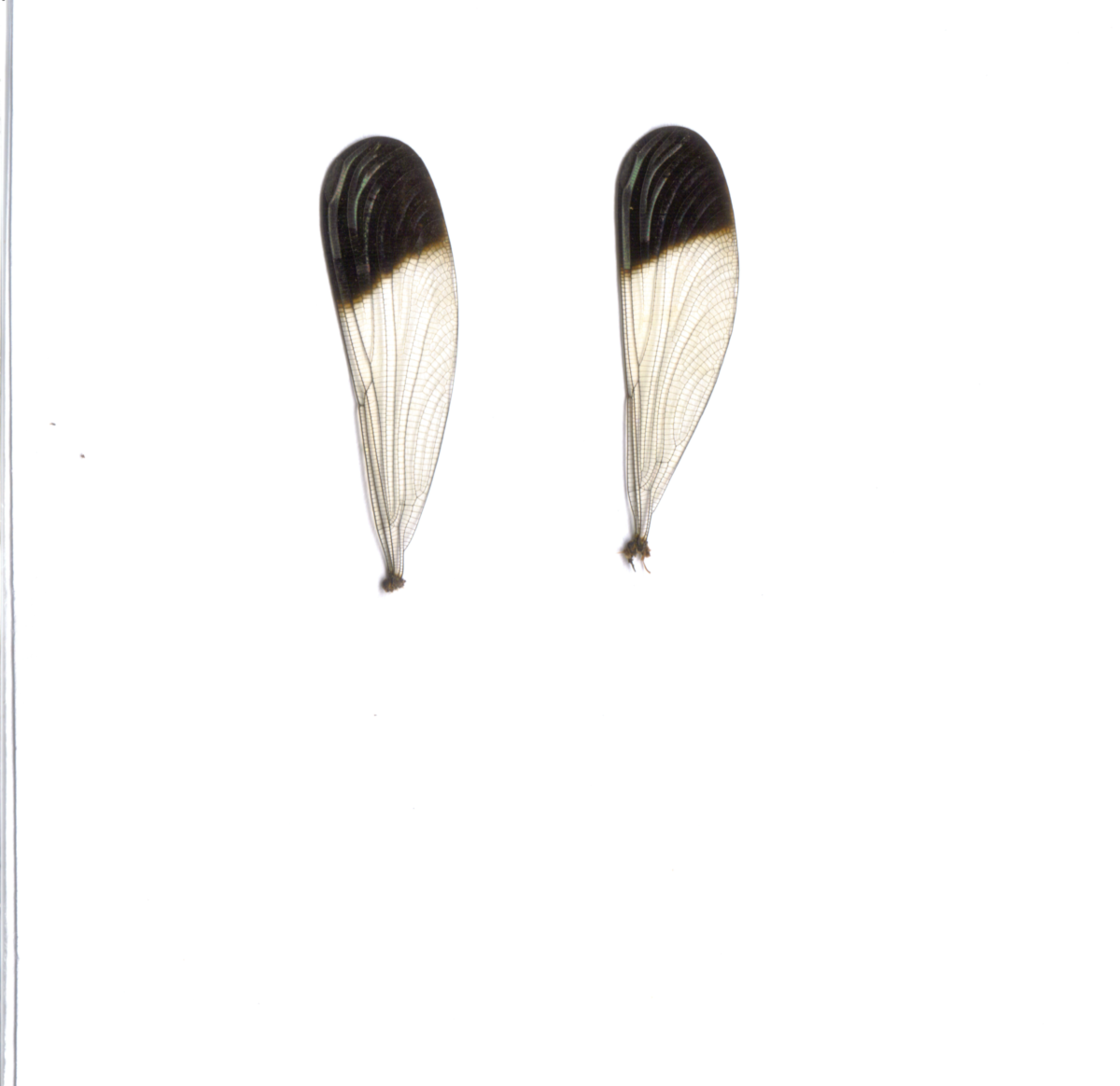

Supplement: S2 File — Compressed folder containing everything needed to run the analyses presented in this paper, including images, data, and a Mathematica notebook. (ZIP) [file pone.0125074.s002.zip › Supplementary file/images/PP78.png]

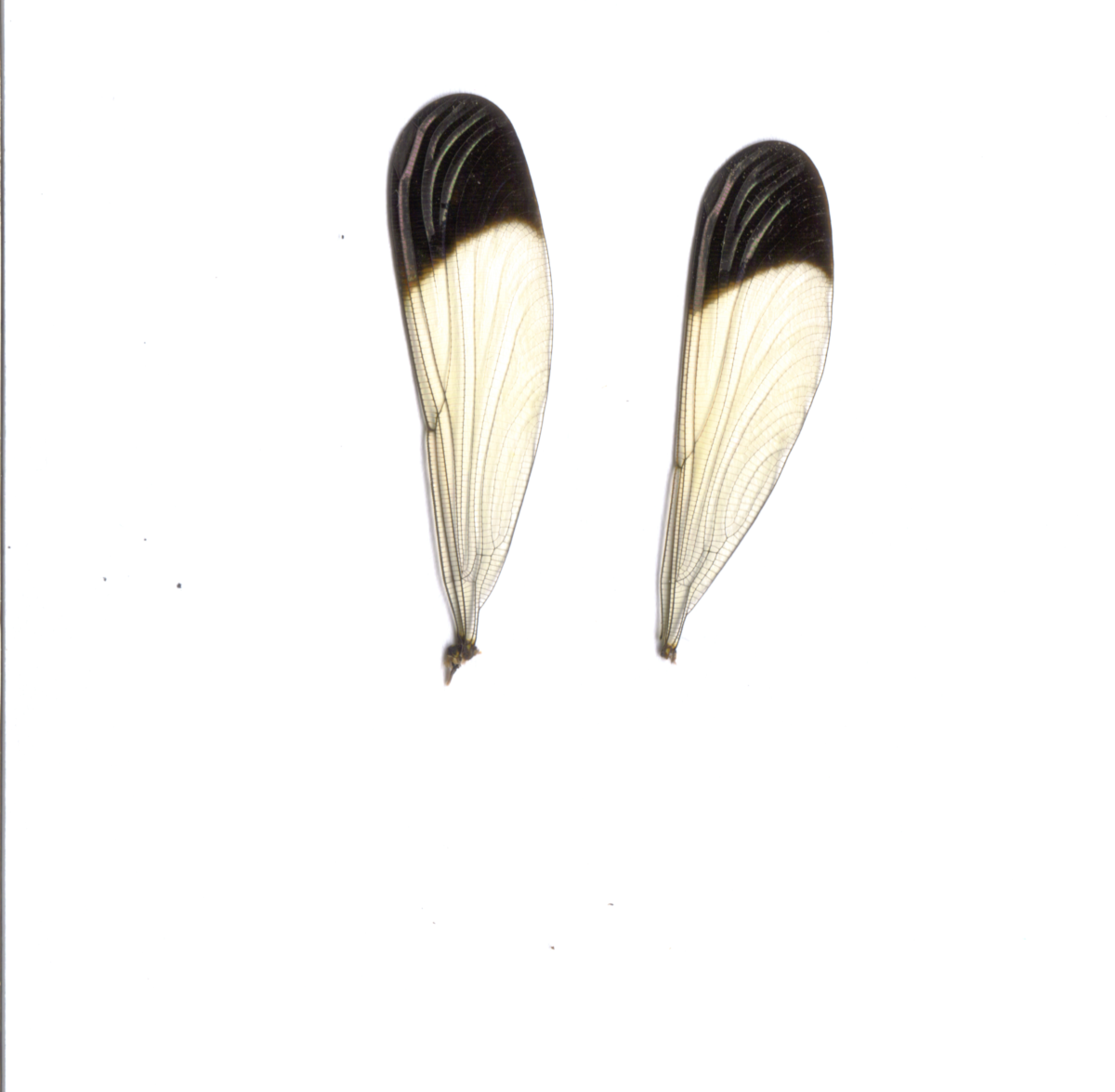

Supplement: S2 File — Compressed folder containing everything needed to run the analyses presented in this paper, including images, data, and a Mathematica notebook. (ZIP) [file pone.0125074.s002.zip › Supplementary file/images/PP9.png]

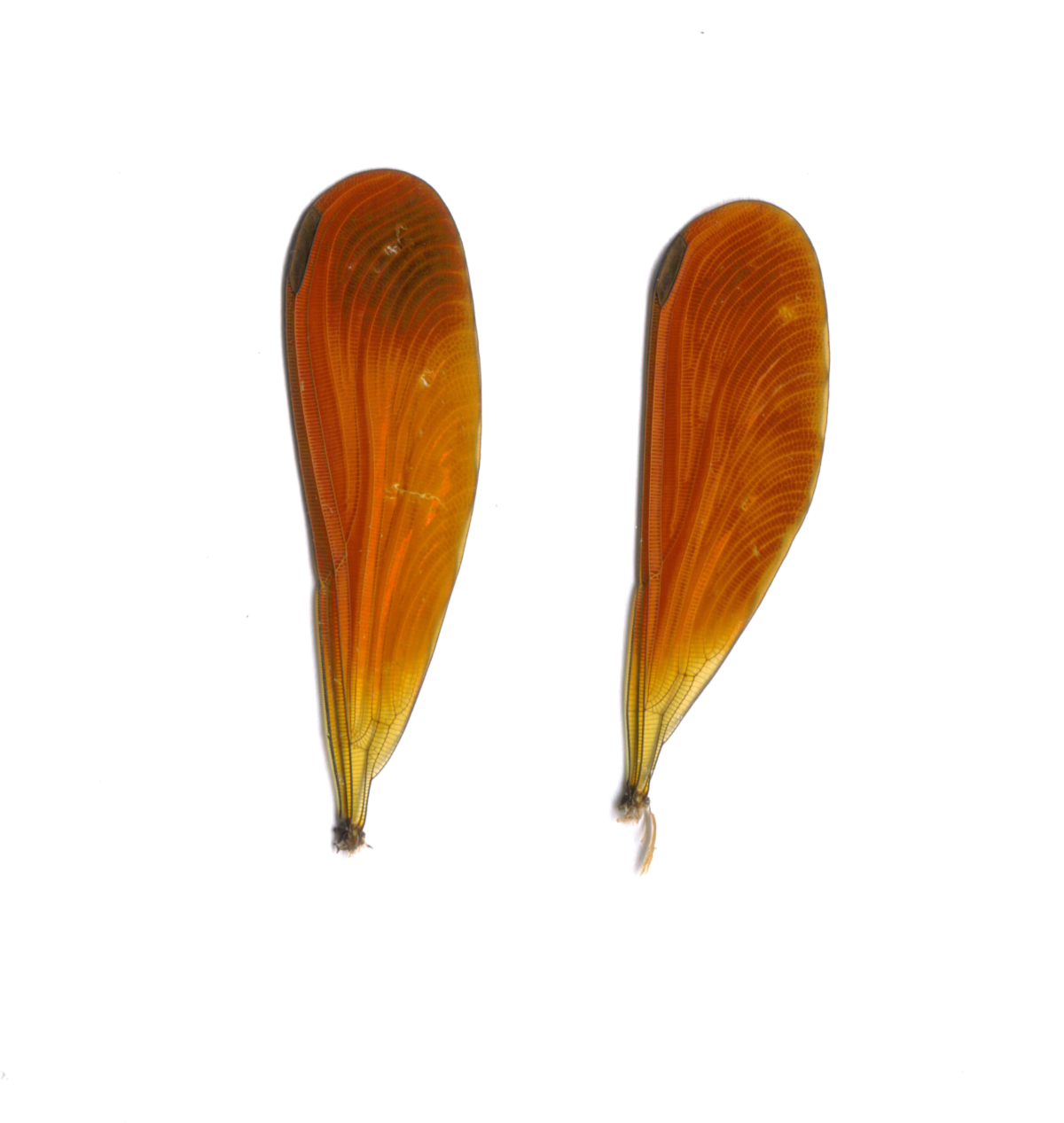

Supplement: S2 File — Compressed folder containing everything needed to run the analyses presented in this paper, including images, data, and a Mathematica notebook. (ZIP) [file pone.0125074.s002.zip › Supplementary file/images/PS06.png]

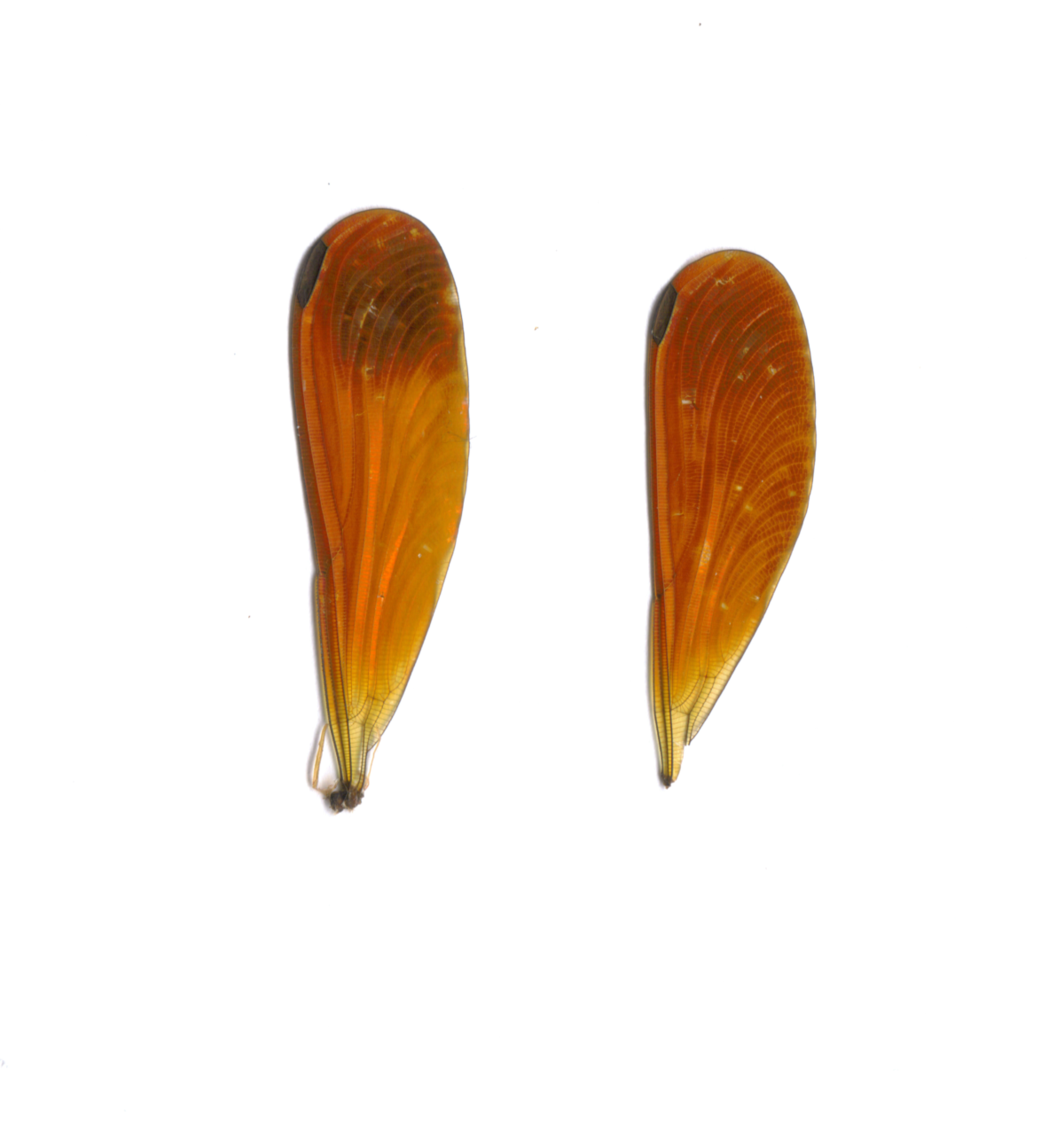

Supplement: S2 File — Compressed folder containing everything needed to run the analyses presented in this paper, including images, data, and a Mathematica notebook. (ZIP) [file pone.0125074.s002.zip › Supplementary file/images/PS07.png]

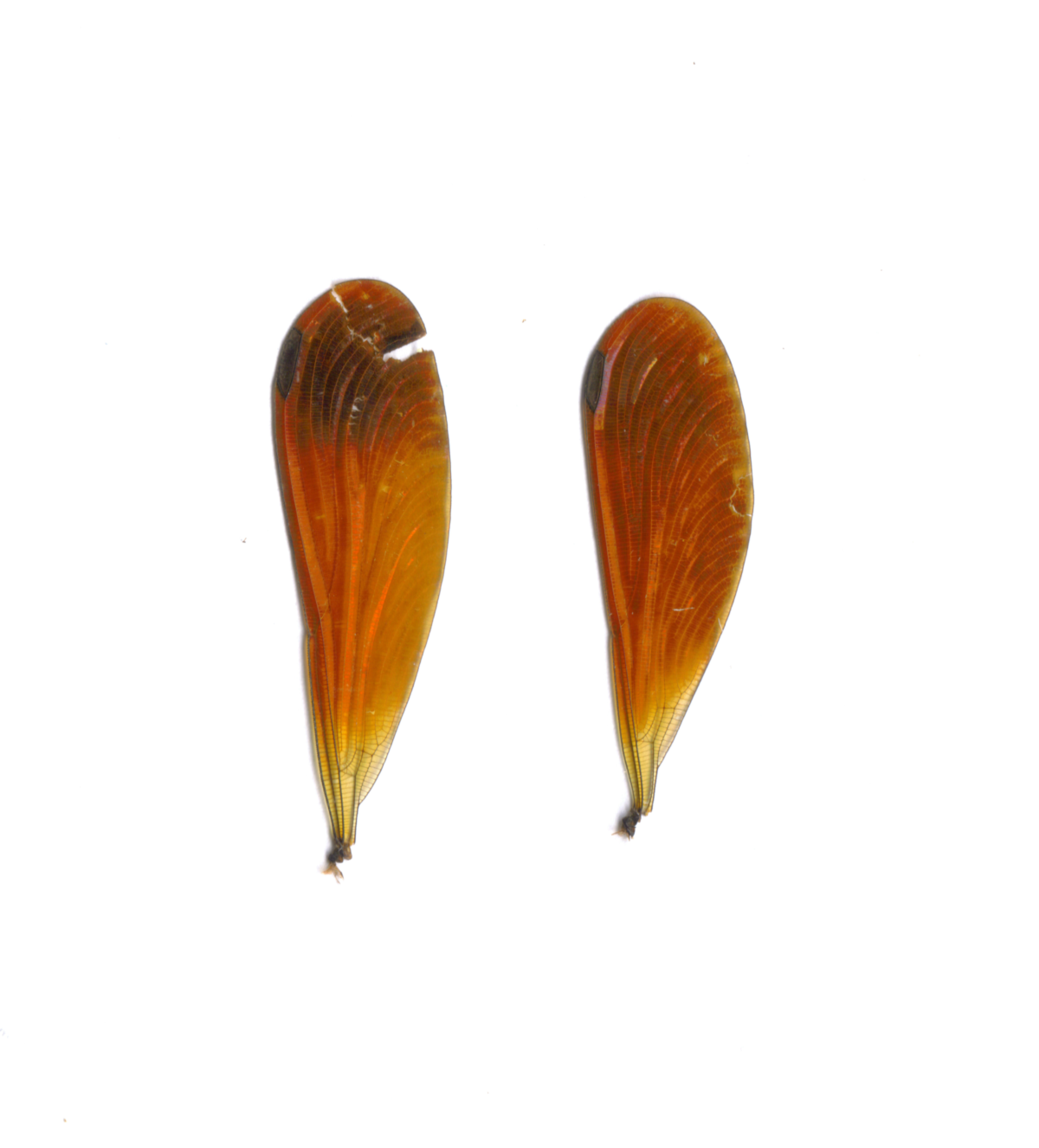

Supplement: S2 File — Compressed folder containing everything needed to run the analyses presented in this paper, including images, data, and a Mathematica notebook. (ZIP) [file pone.0125074.s002.zip › Supplementary file/images/PS08.png]

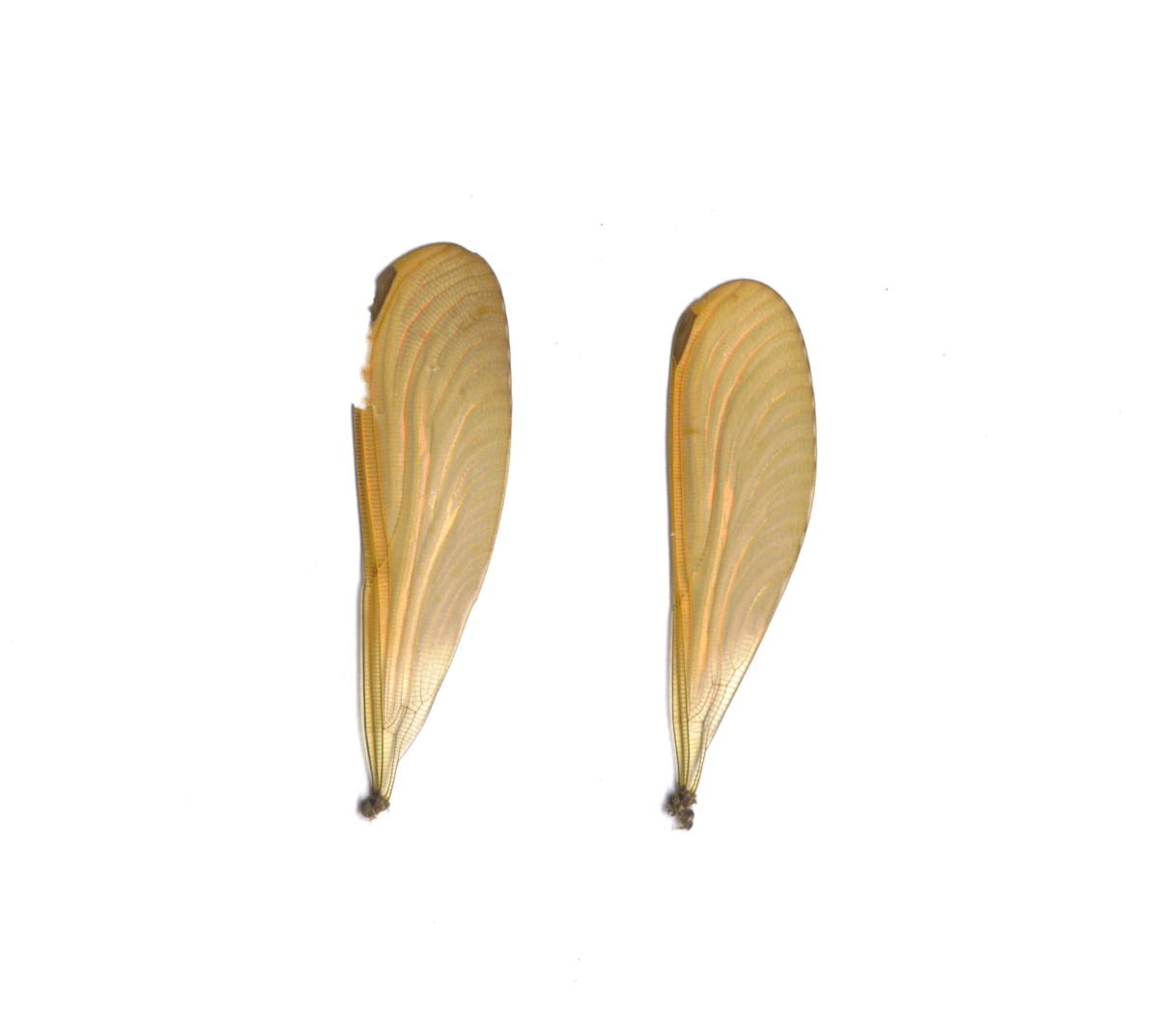

Supplement: S2 File — Compressed folder containing everything needed to run the analyses presented in this paper, including images, data, and a Mathematica notebook. (ZIP) [file pone.0125074.s002.zip › Supplementary file/images/PS09.png]

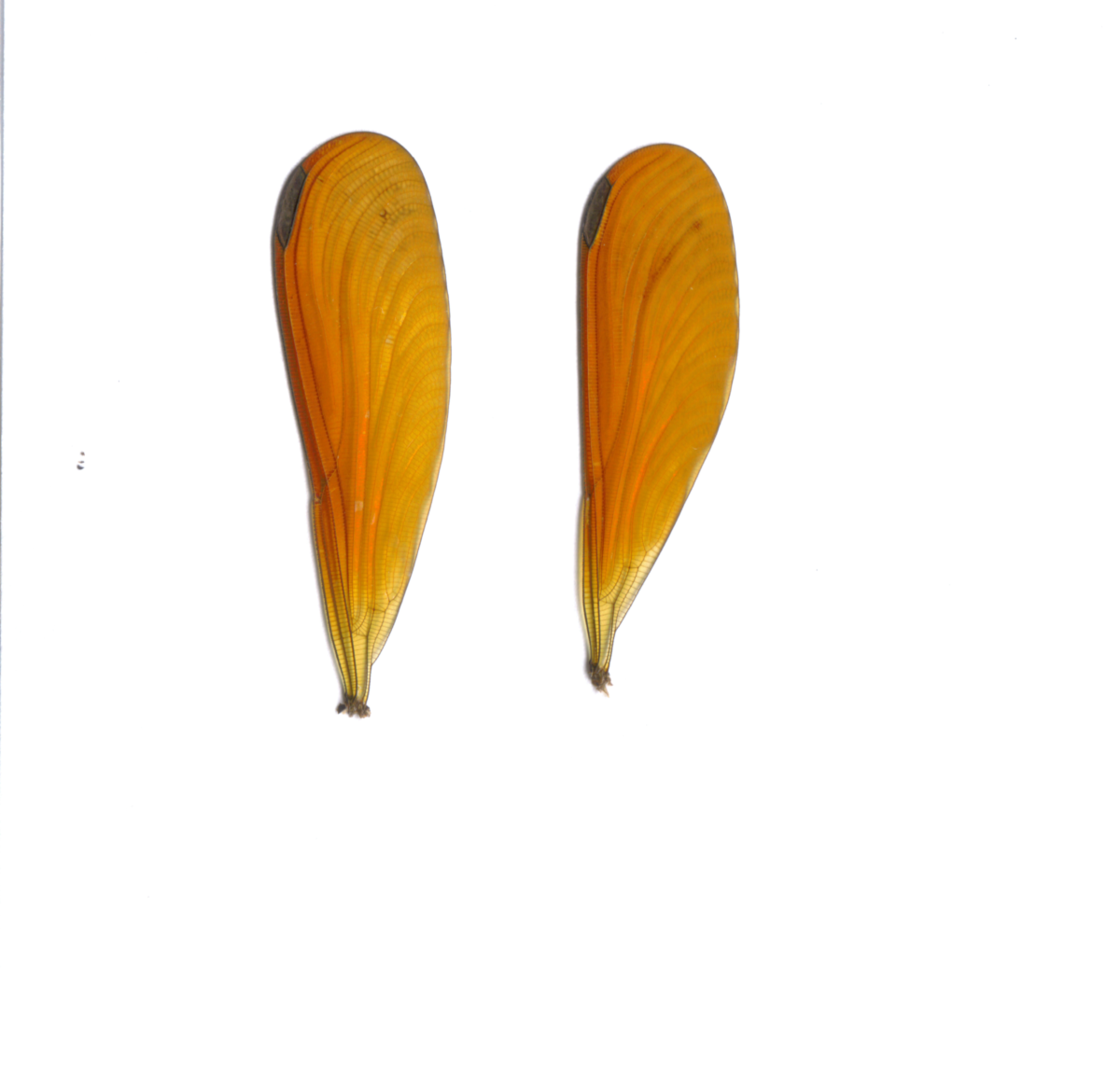

Supplement: S2 File — Compressed folder containing everything needed to run the analyses presented in this paper, including images, data, and a Mathematica notebook. (ZIP) [file pone.0125074.s002.zip › Supplementary file/images/PS54.png]

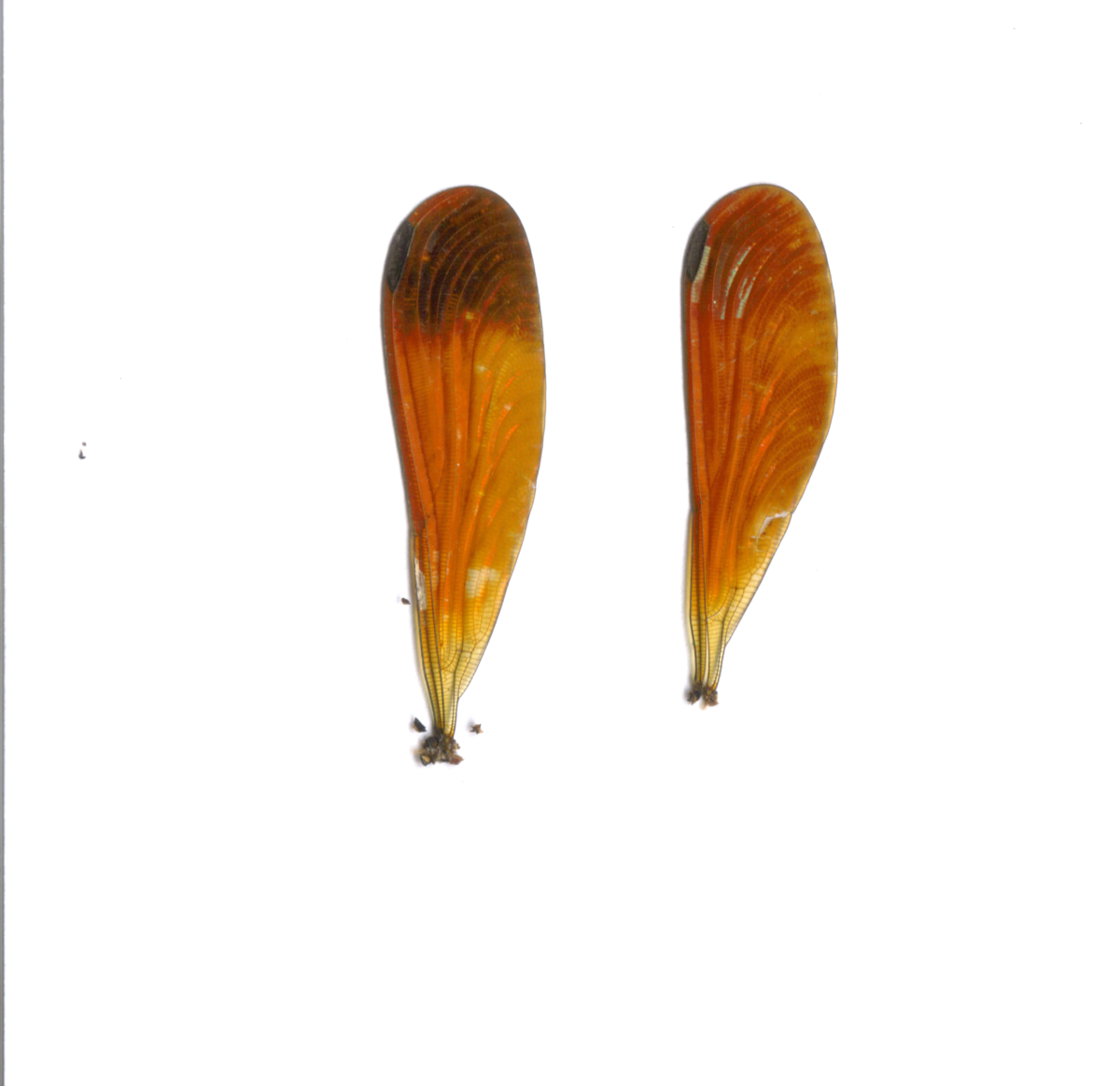

Supplement: S2 File — Compressed folder containing everything needed to run the analyses presented in this paper, including images, data, and a Mathematica notebook. (ZIP) [file pone.0125074.s002.zip › Supplementary file/images/PS57.png]

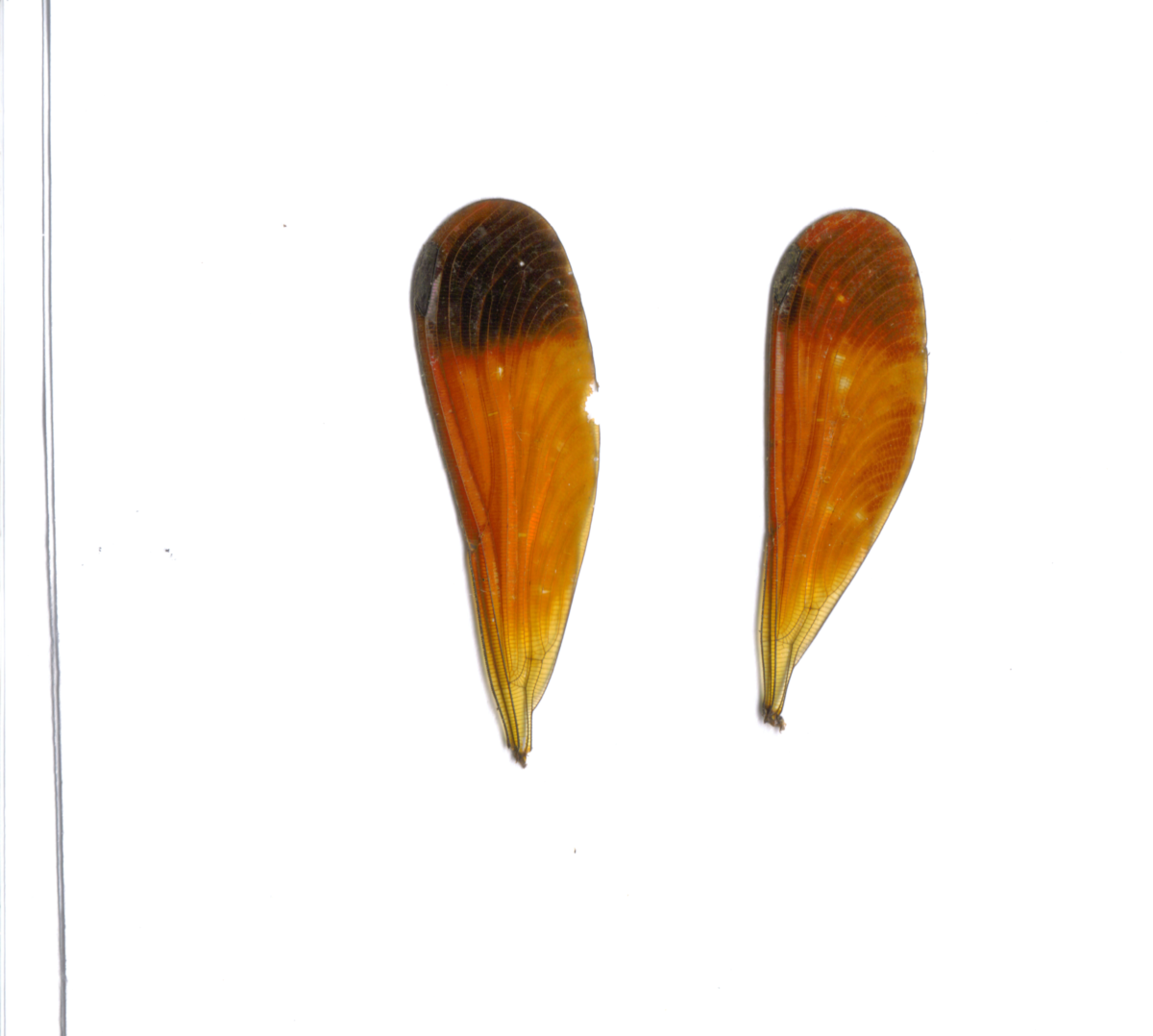

Supplement: S2 File — Compressed folder containing everything needed to run the analyses presented in this paper, including images, data, and a Mathematica notebook. (ZIP) [file pone.0125074.s002.zip › Supplementary file/images/PS90.png]

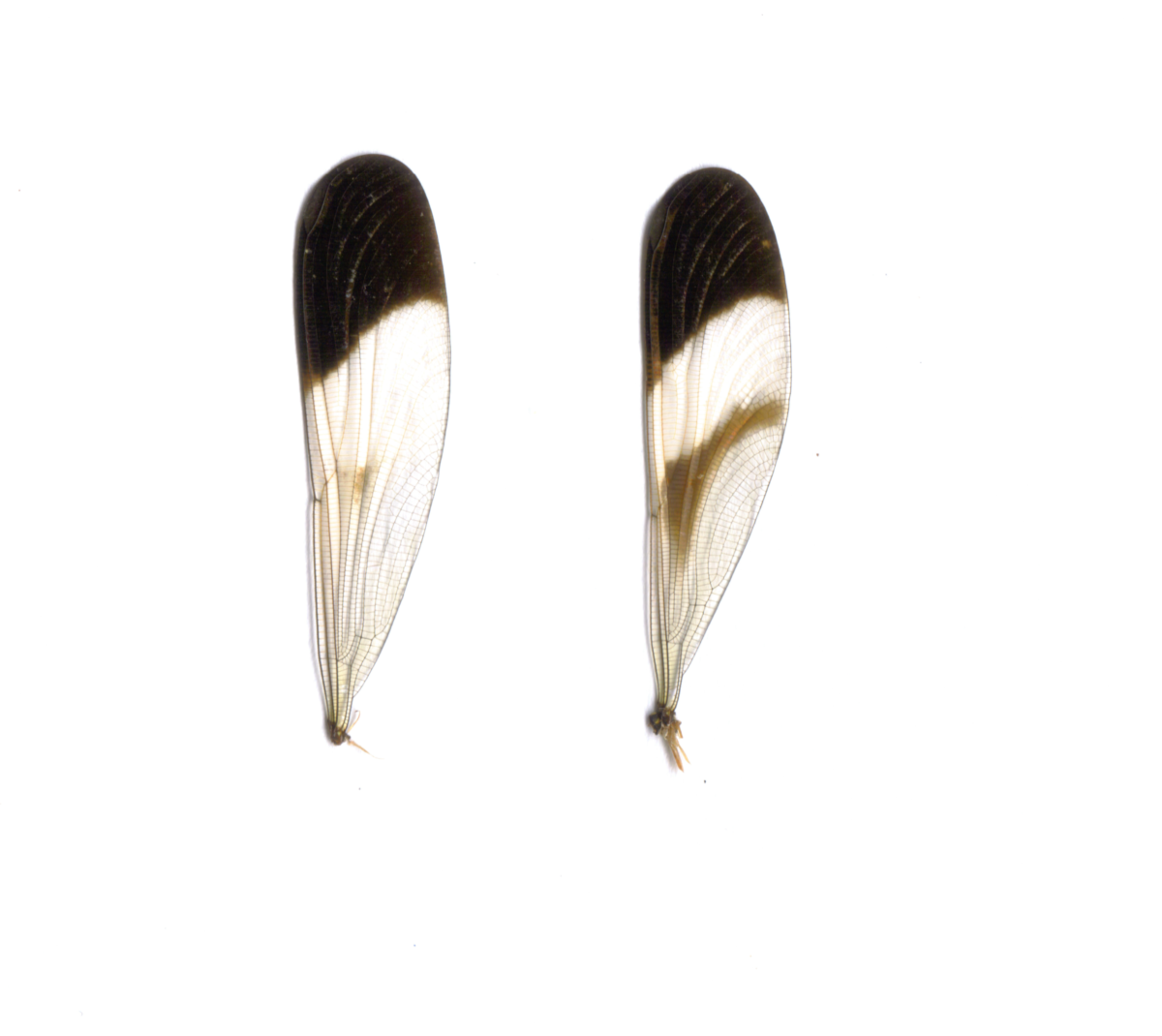

Supplement: S2 File — Compressed folder containing everything needed to run the analyses presented in this paper, including images, data, and a Mathematica notebook. (ZIP) [file pone.0125074.s002.zip › Supplementary file/images/PV12.png]

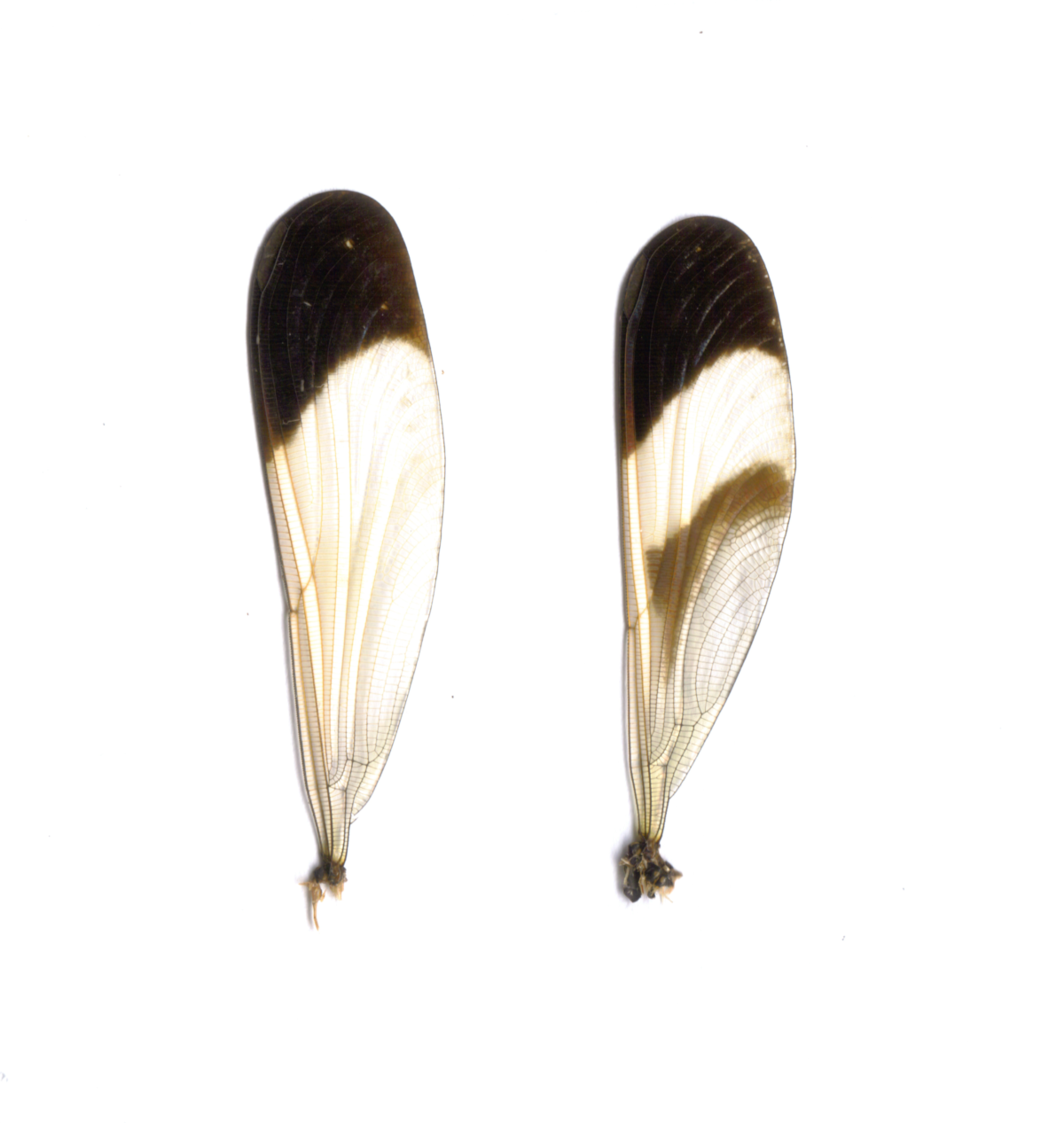

Supplement: S2 File — Compressed folder containing everything needed to run the analyses presented in this paper, including images, data, and a Mathematica notebook. (ZIP) [file pone.0125074.s002.zip › Supplementary file/images/PV13.png]

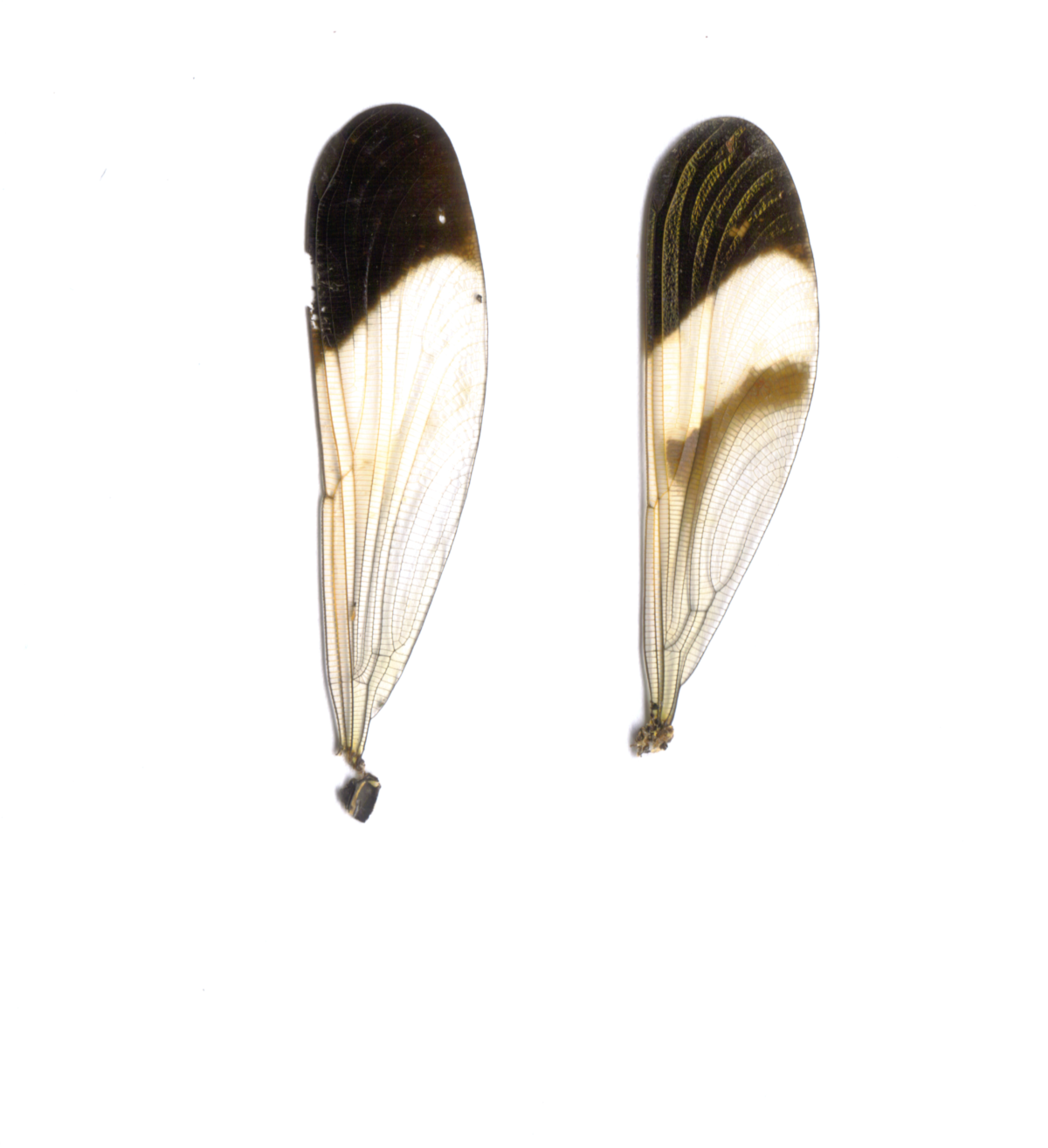

Supplement: S2 File — Compressed folder containing everything needed to run the analyses presented in this paper, including images, data, and a Mathematica notebook. (ZIP) [file pone.0125074.s002.zip › Supplementary file/images/PV15.png]

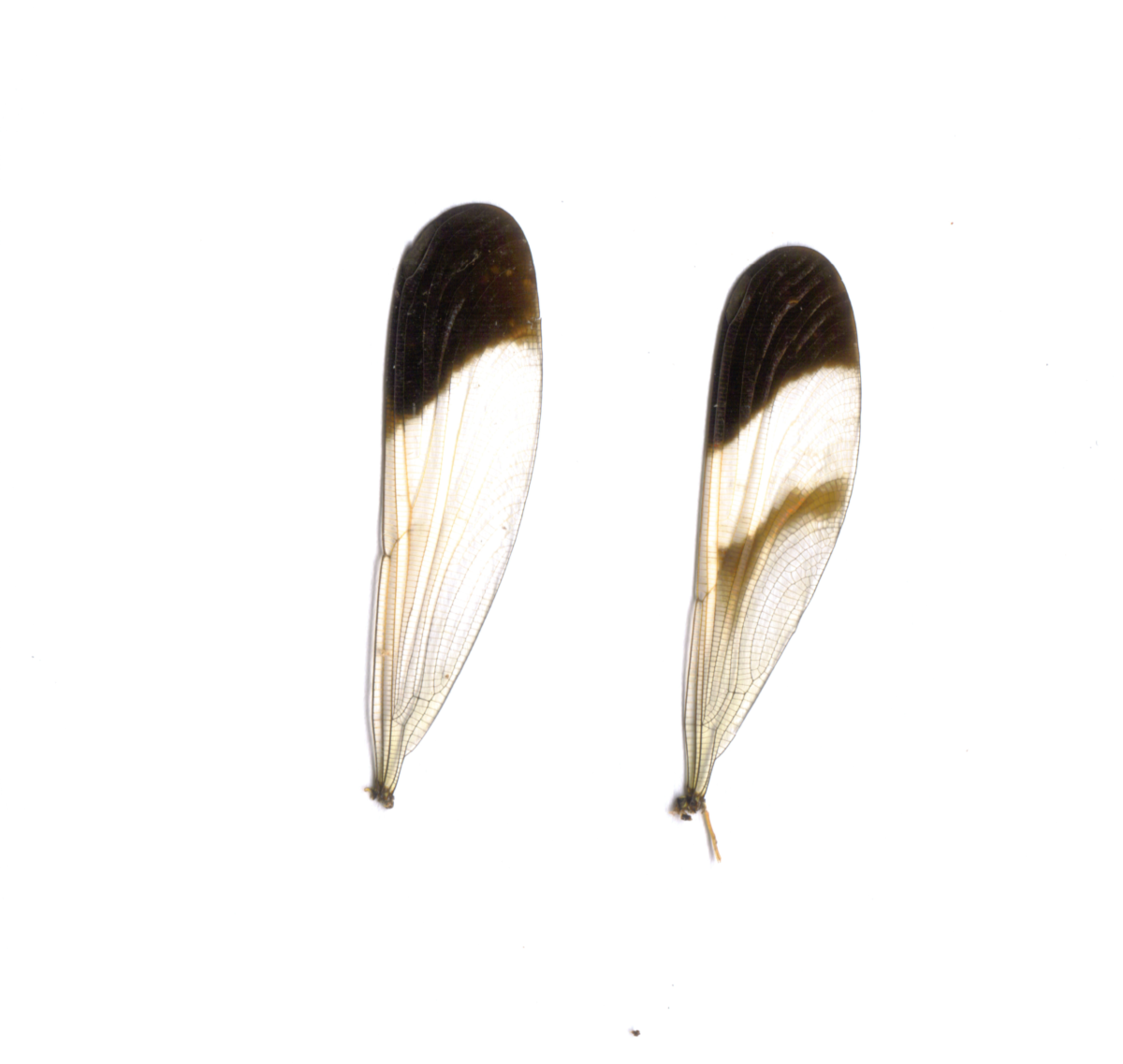

Supplement: S2 File — Compressed folder containing everything needed to run the analyses presented in this paper, including images, data, and a Mathematica notebook. (ZIP) [file pone.0125074.s002.zip › Supplementary file/images/PV16.png]

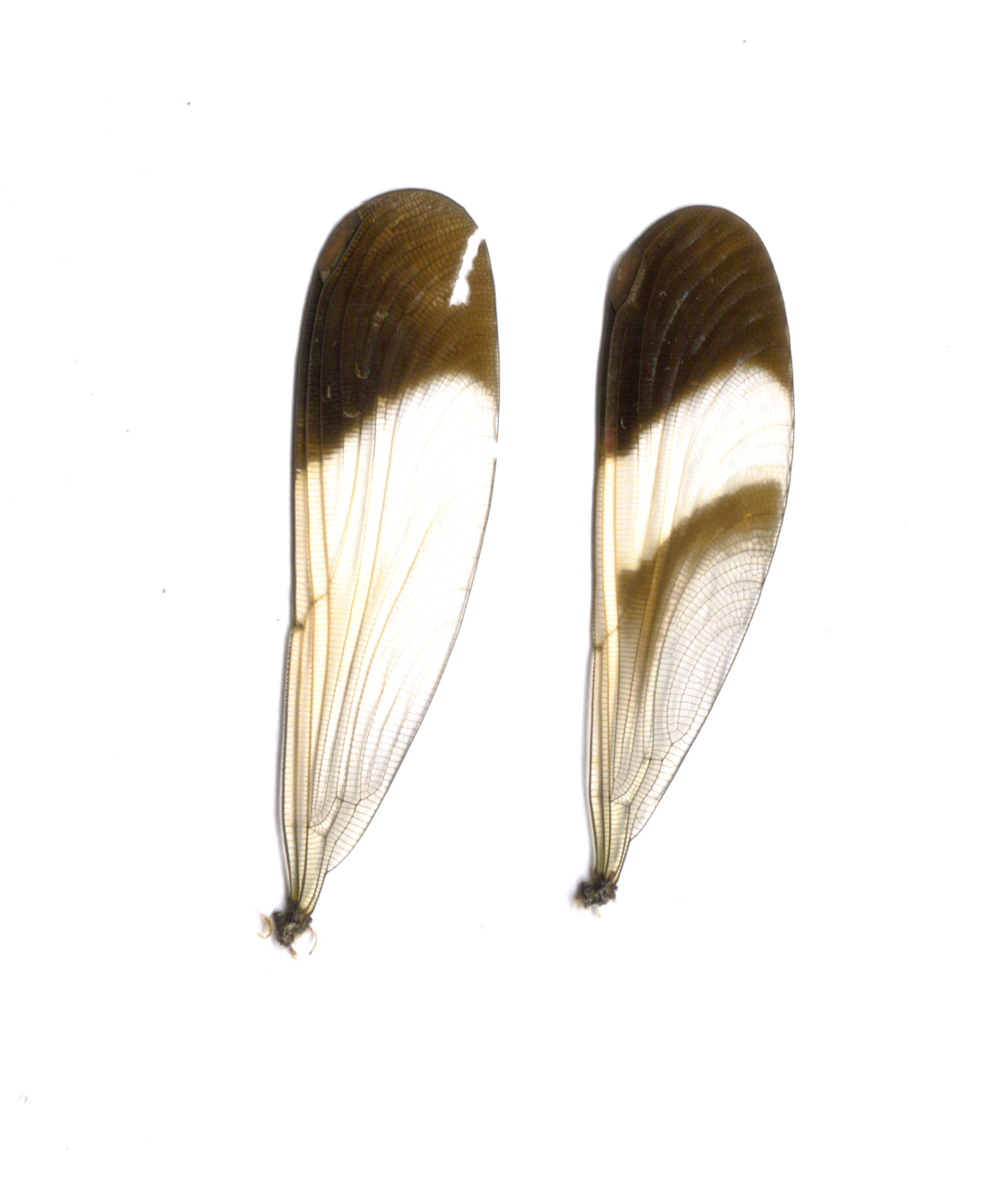

Supplement: S2 File — Compressed folder containing everything needed to run the analyses presented in this paper, including images, data, and a Mathematica notebook. (ZIP) [file pone.0125074.s002.zip › Supplementary file/images/PV18.png]

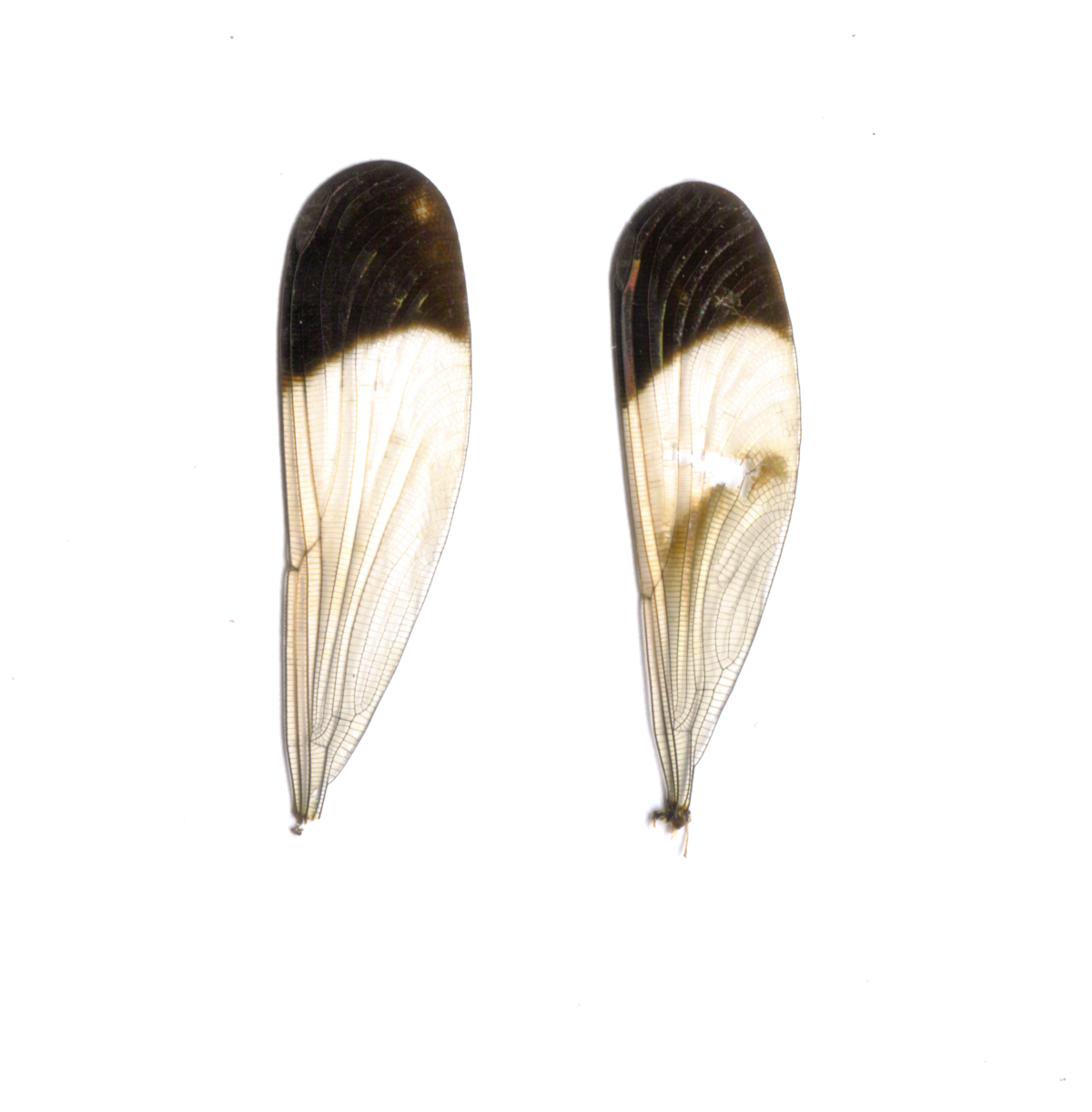

Supplement: S2 File — Compressed folder containing everything needed to run the analyses presented in this paper, including images, data, and a Mathematica notebook. (ZIP) [file pone.0125074.s002.zip › Supplementary file/images/PV19.png]

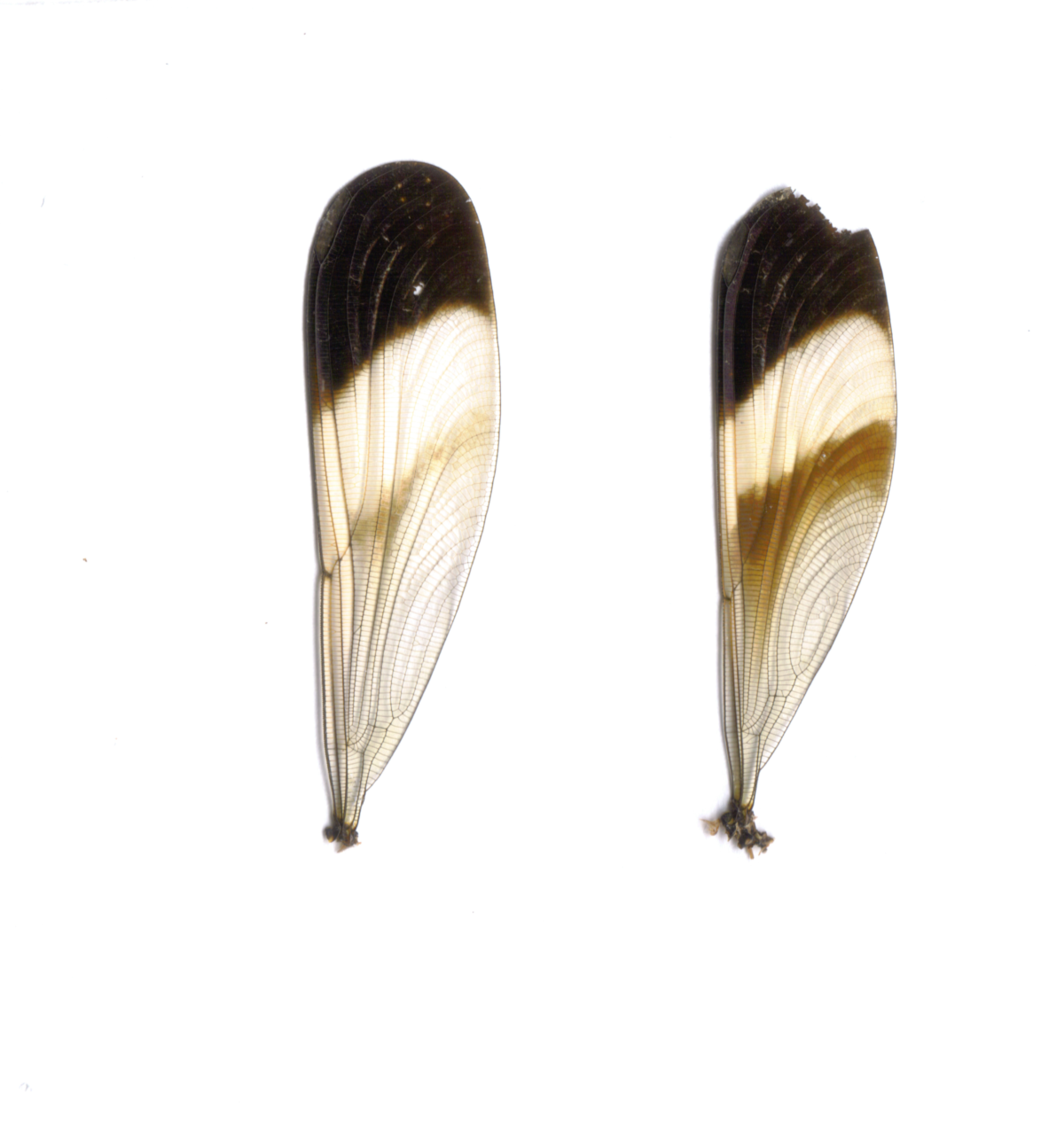

Supplement: S2 File — Compressed folder containing everything needed to run the analyses presented in this paper, including images, data, and a Mathematica notebook. (ZIP) [file pone.0125074.s002.zip › Supplementary file/images/PV20.png]

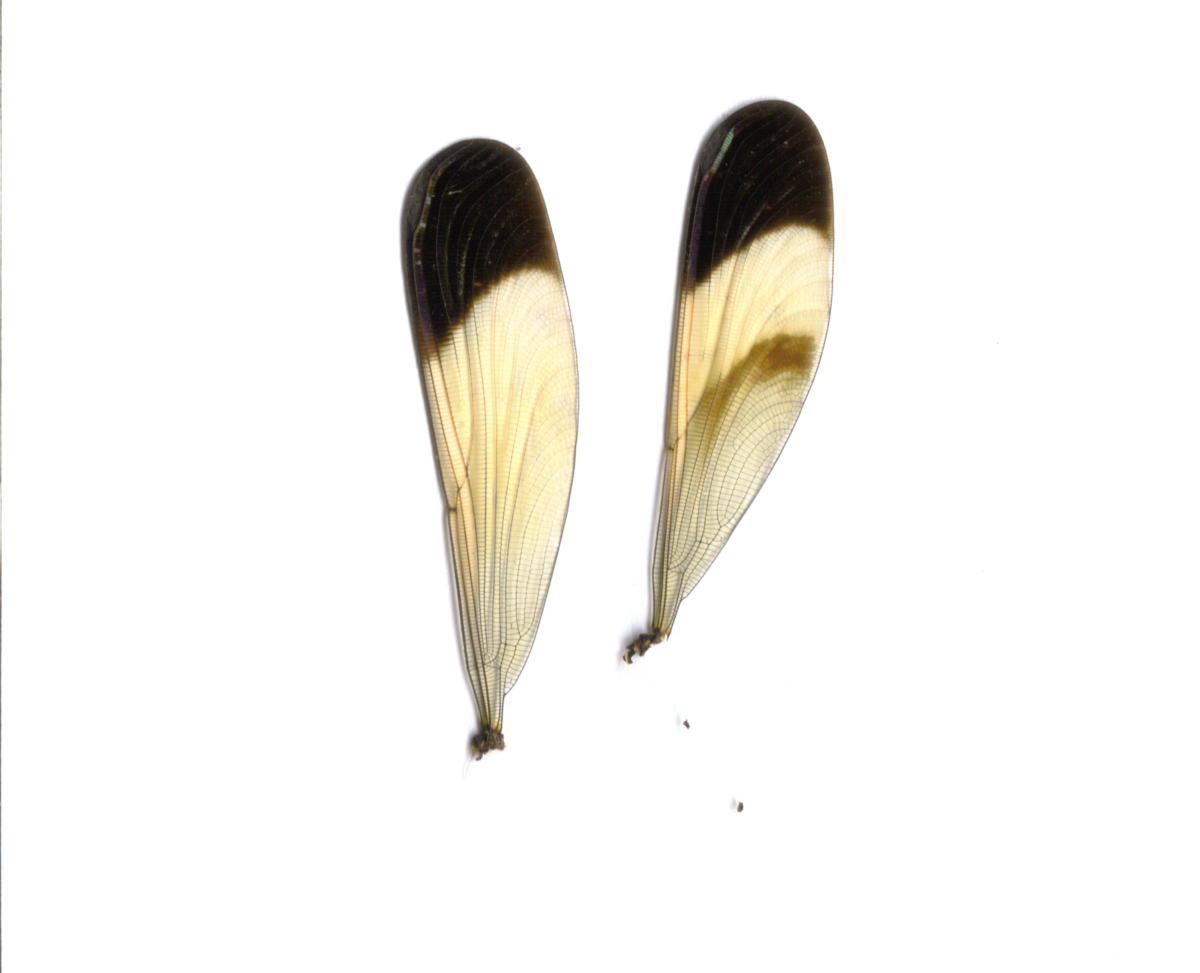

Supplement: S2 File — Compressed folder containing everything needed to run the analyses presented in this paper, including images, data, and a Mathematica notebook. (ZIP) [file pone.0125074.s002.zip › Supplementary file/images/PV72.png]

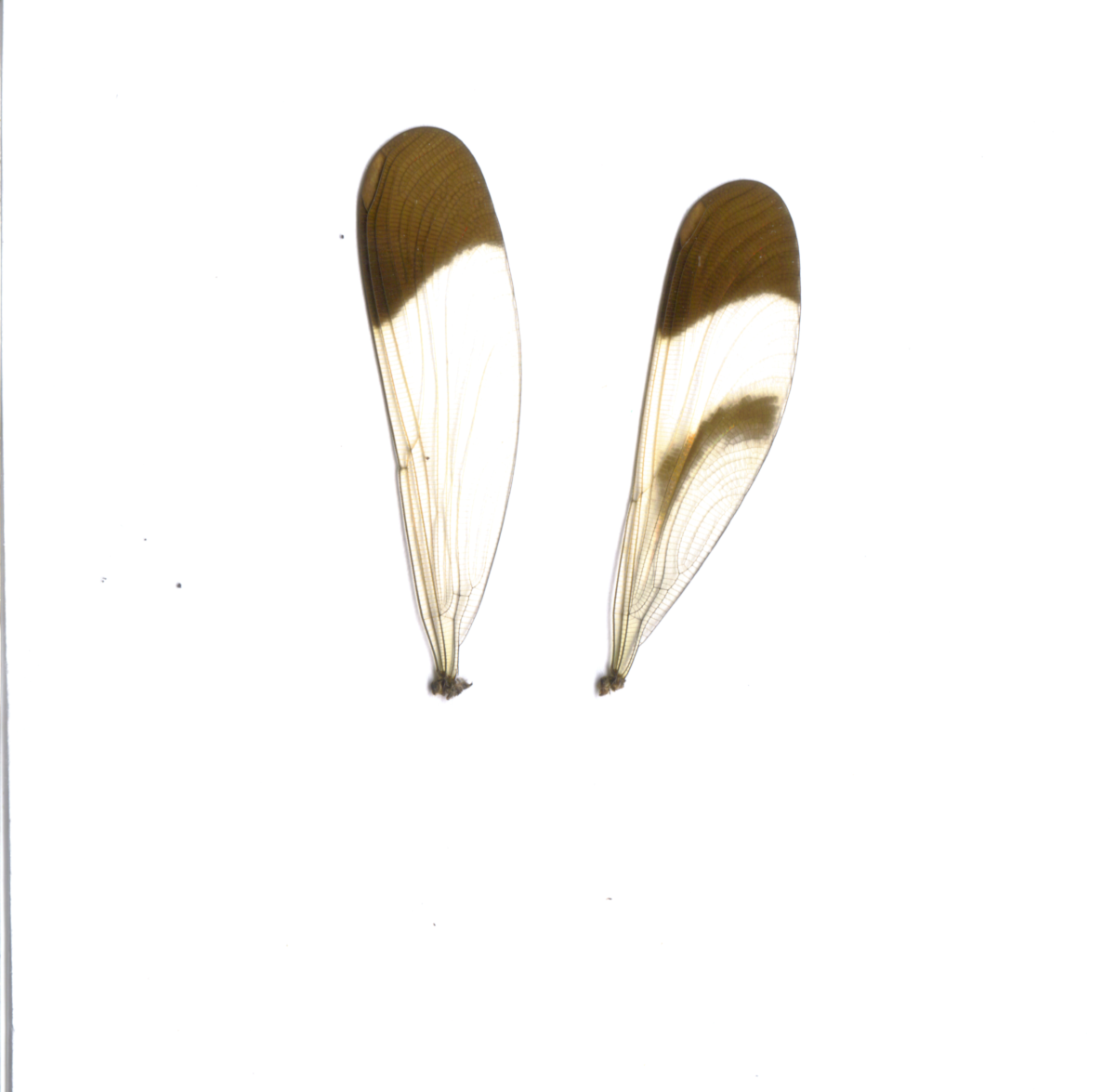

Supplement: S2 File — Compressed folder containing everything needed to run the analyses presented in this paper, including images, data, and a Mathematica notebook. (ZIP) [file pone.0125074.s002.zip › Supplementary file/images/PV83.png]

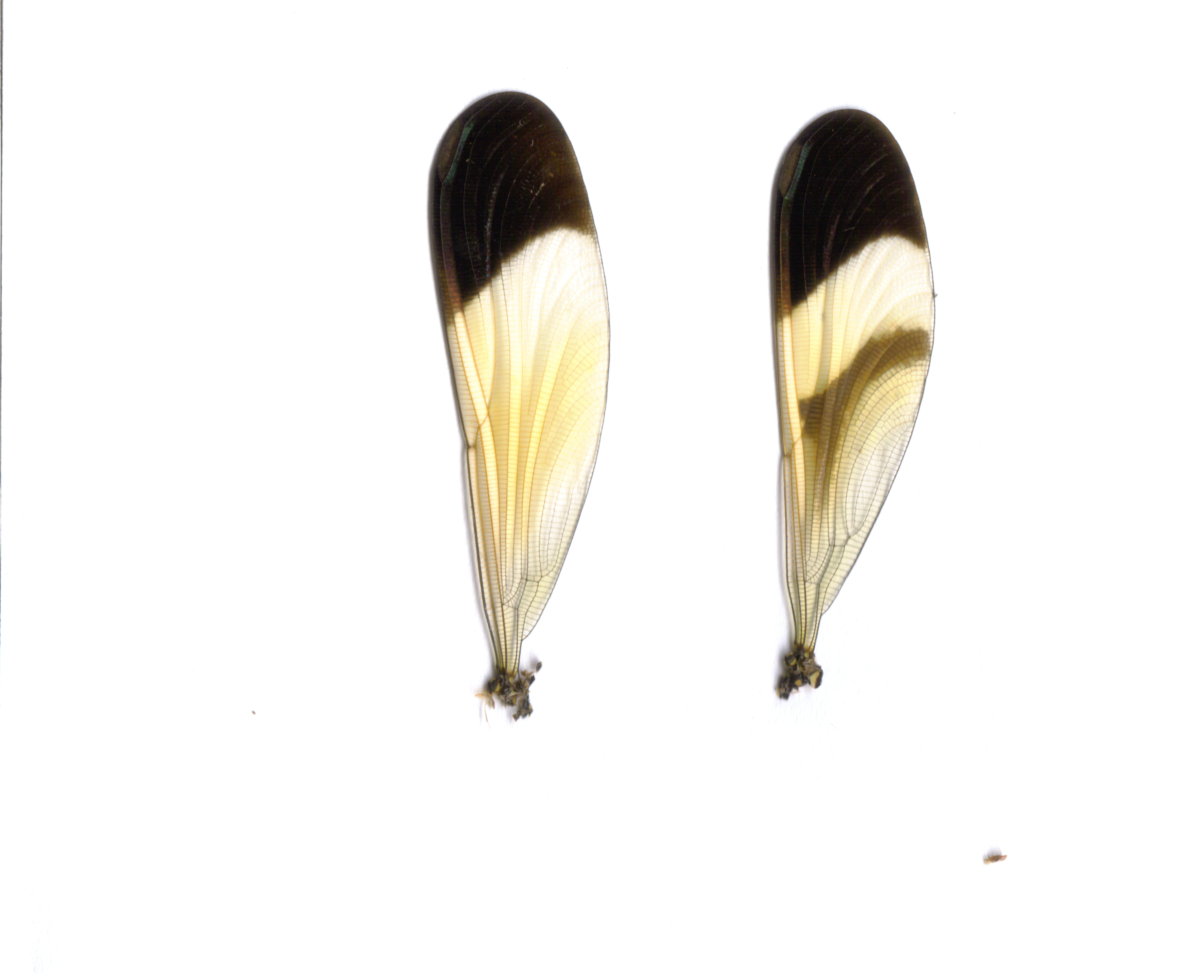

Supplement: S2 File — Compressed folder containing everything needed to run the analyses presented in this paper, including images, data, and a Mathematica notebook. (ZIP) [file pone.0125074.s002.zip › Supplementary file/images/PV85.png]

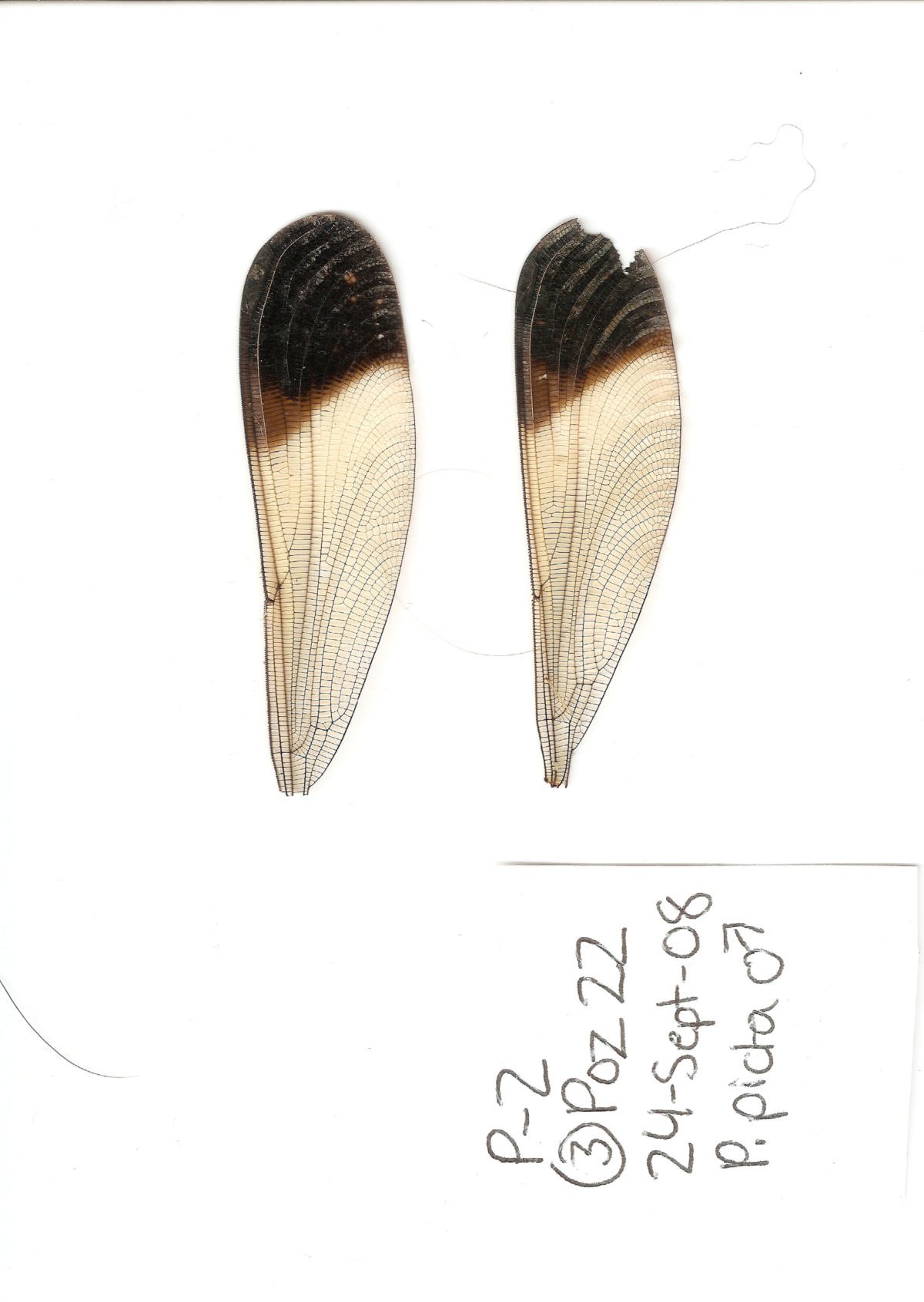

Supplement: S2 File — Compressed folder containing everything needed to run the analyses presented in this paper, including images, data, and a Mathematica notebook. (ZIP) [file pone.0125074.s002.zip › Supplementary file/images/scan0004.png]

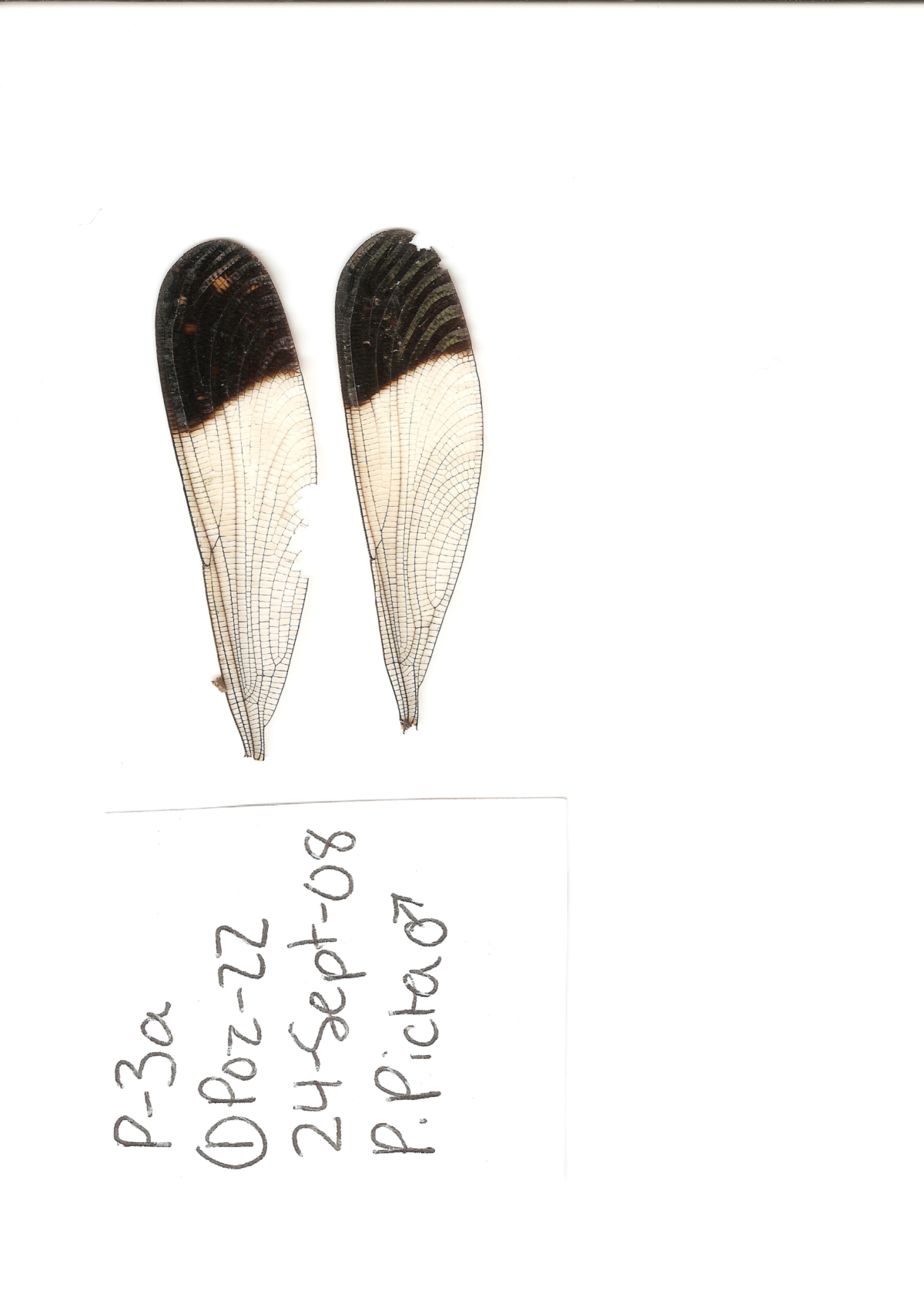

Supplement: S2 File — Compressed folder containing everything needed to run the analyses presented in this paper, including images, data, and a Mathematica notebook. (ZIP) [file pone.0125074.s002.zip › Supplementary file/images/scan0005.png]

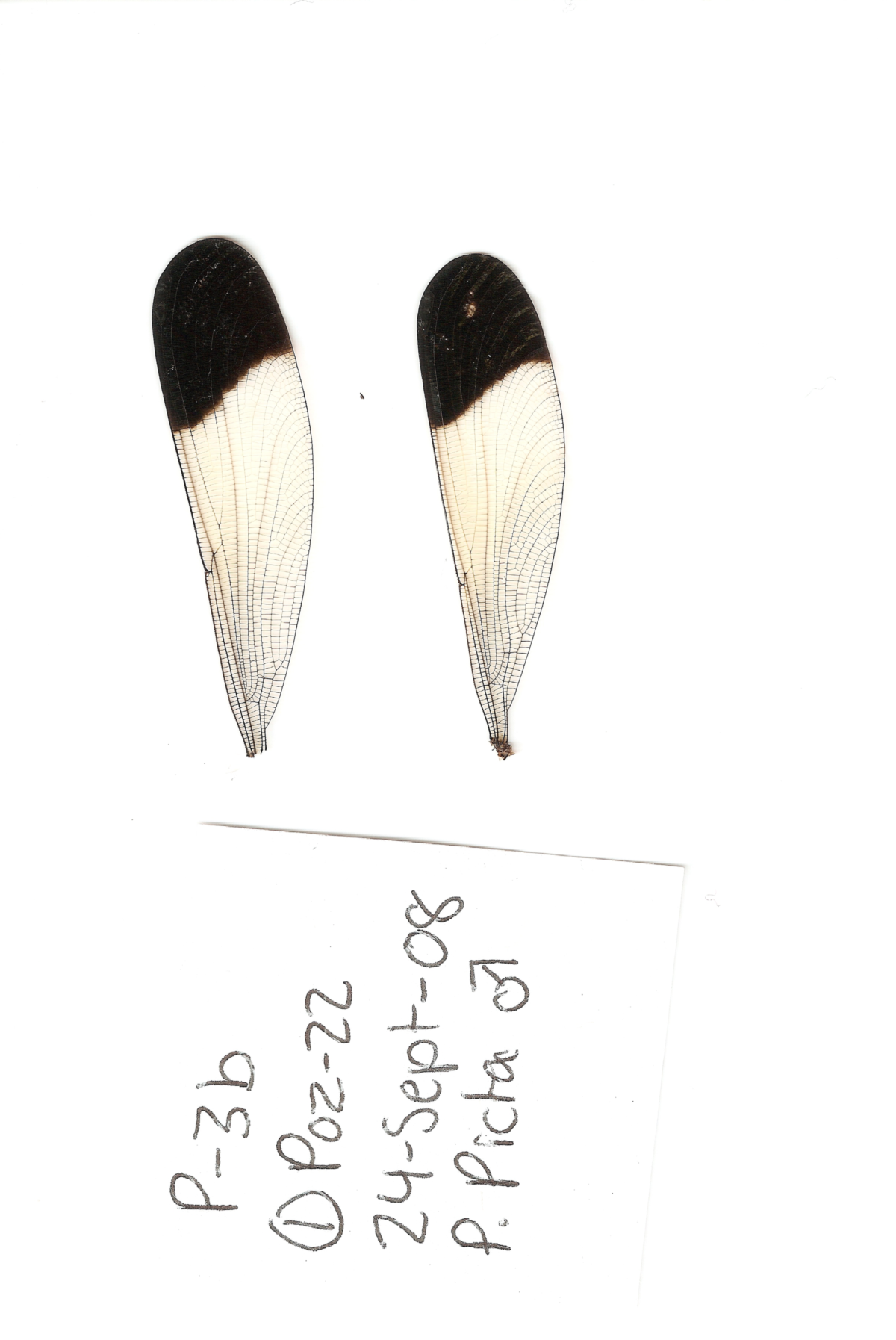

Supplement: S2 File — Compressed folder containing everything needed to run the analyses presented in this paper, including images, data, and a Mathematica notebook. (ZIP) [file pone.0125074.s002.zip › Supplementary file/images/scan0006.png]

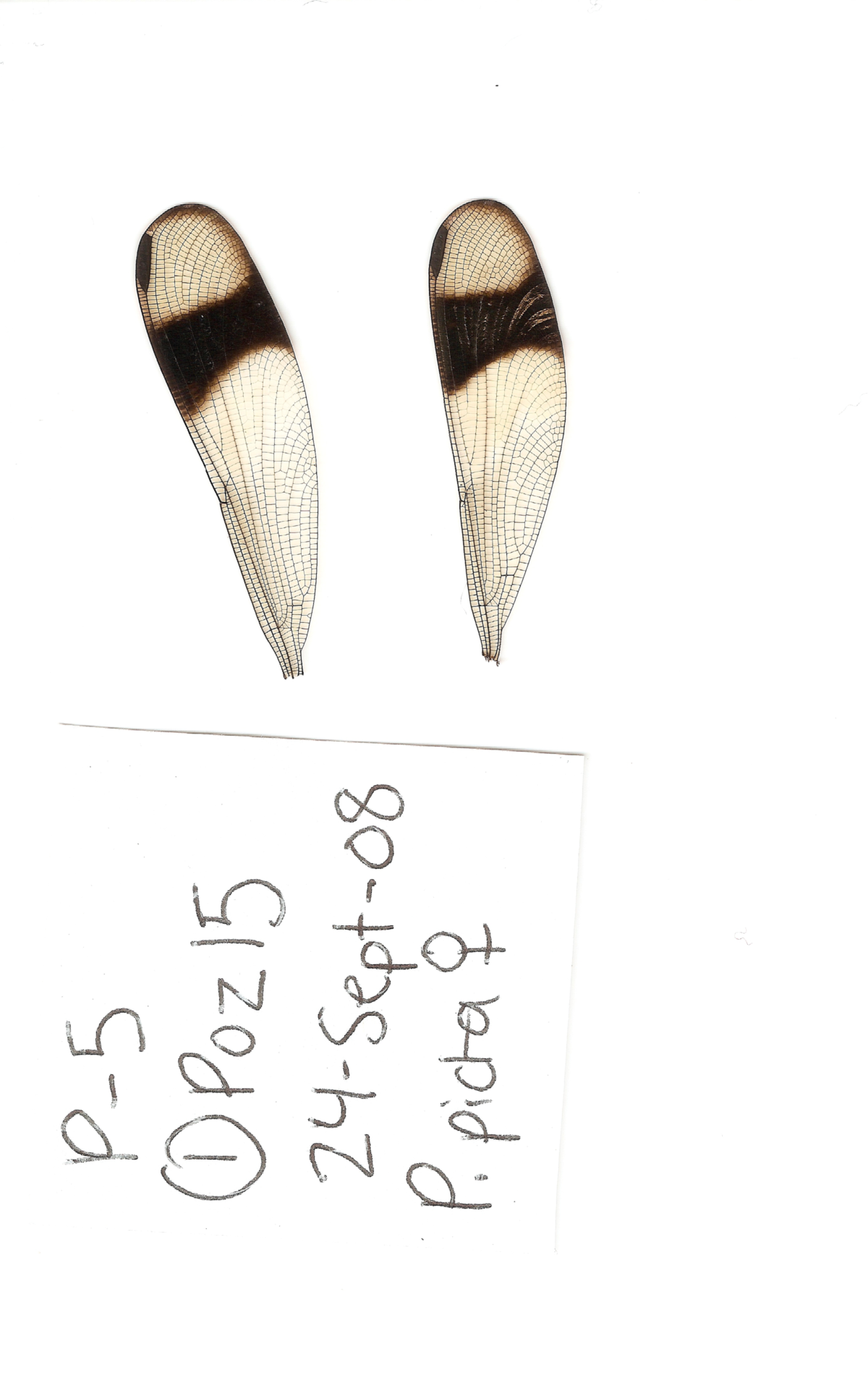

Supplement: S2 File — Compressed folder containing everything needed to run the analyses presented in this paper, including images, data, and a Mathematica notebook. (ZIP) [file pone.0125074.s002.zip › Supplementary file/images/scan0007.png]

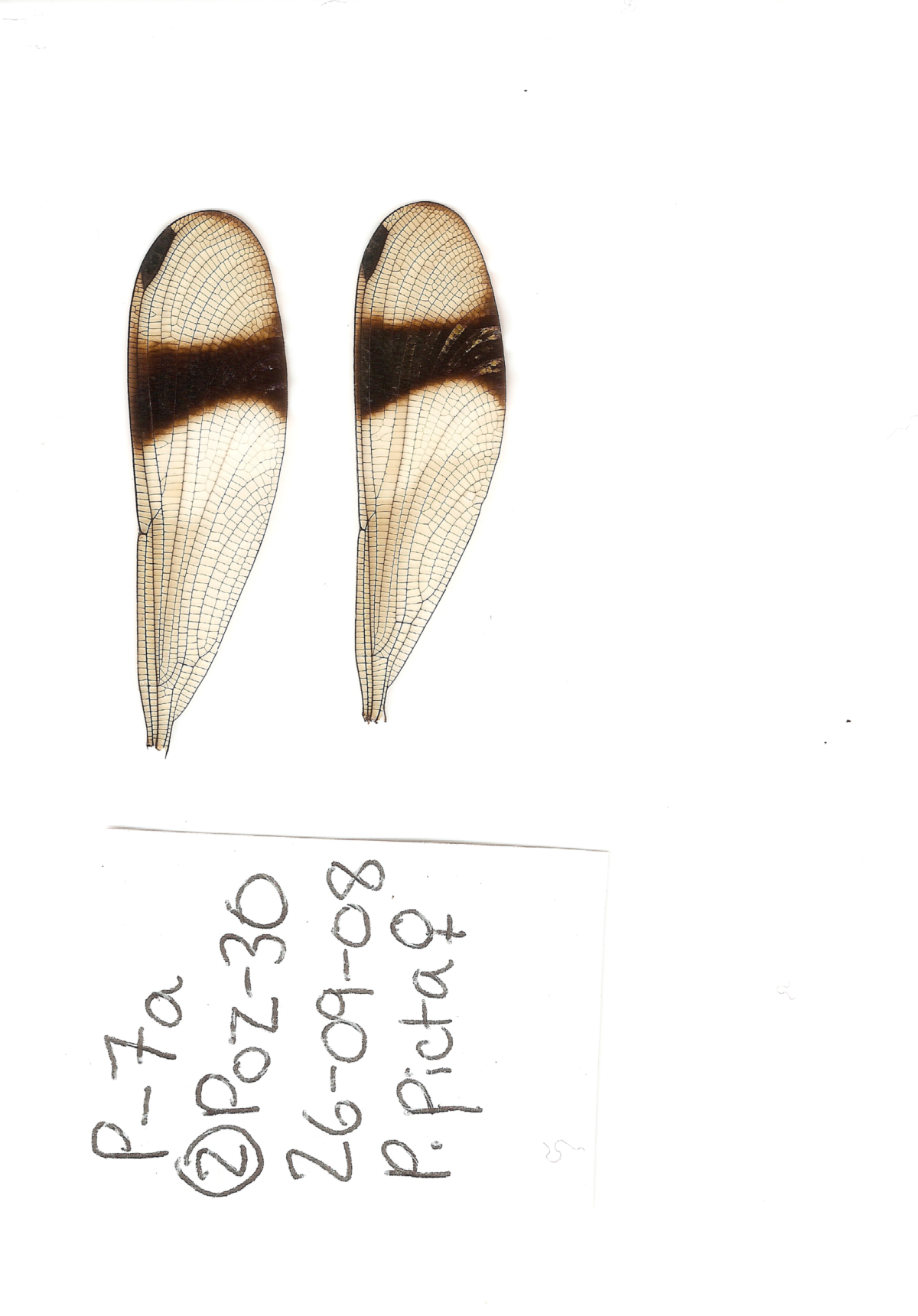

Supplement: S2 File — Compressed folder containing everything needed to run the analyses presented in this paper, including images, data, and a Mathematica notebook. (ZIP) [file pone.0125074.s002.zip › Supplementary file/images/scan0008.png]

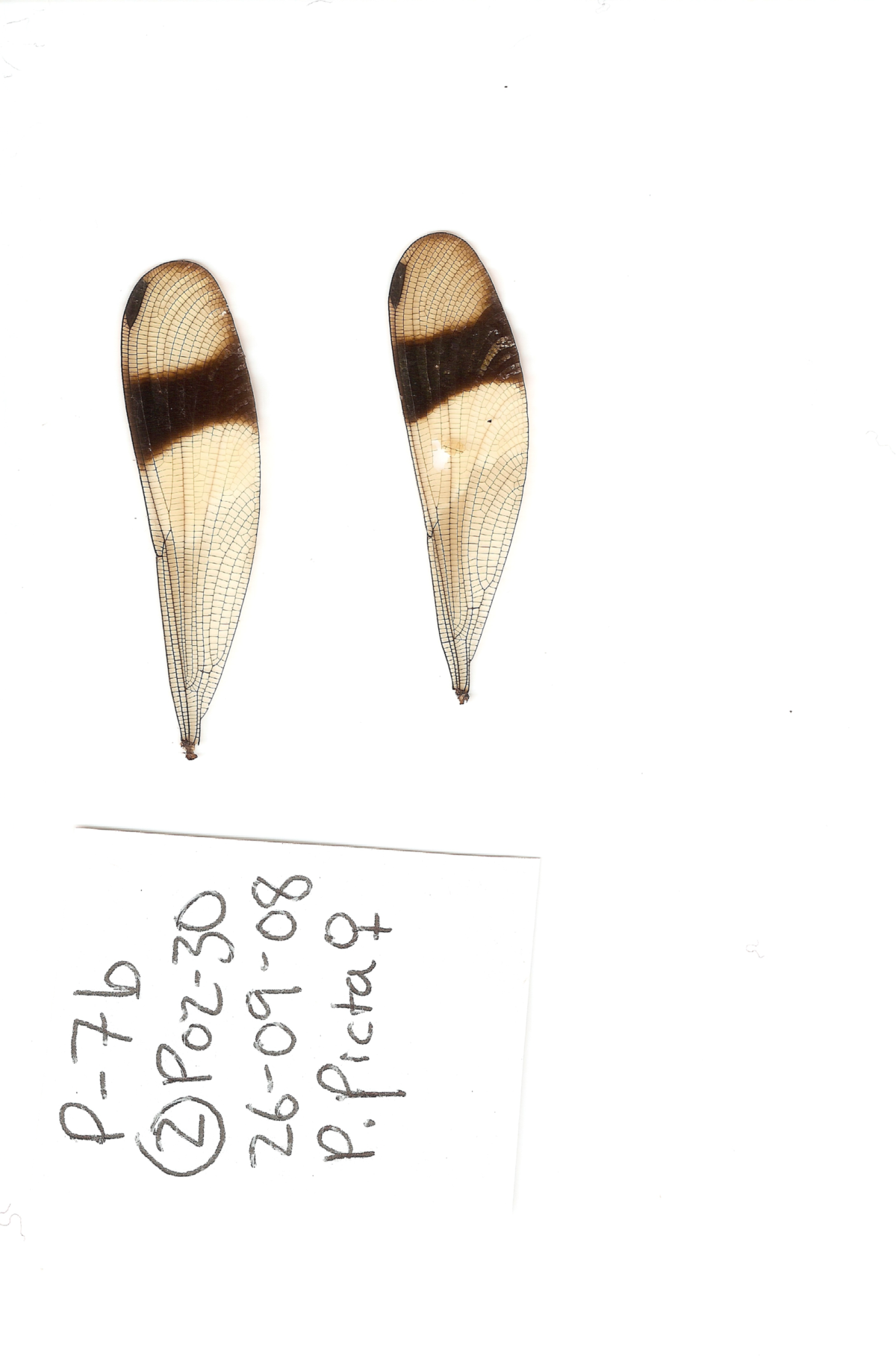

Supplement: S2 File — Compressed folder containing everything needed to run the analyses presented in this paper, including images, data, and a Mathematica notebook. (ZIP) [file pone.0125074.s002.zip › Supplementary file/images/scan0009.png]

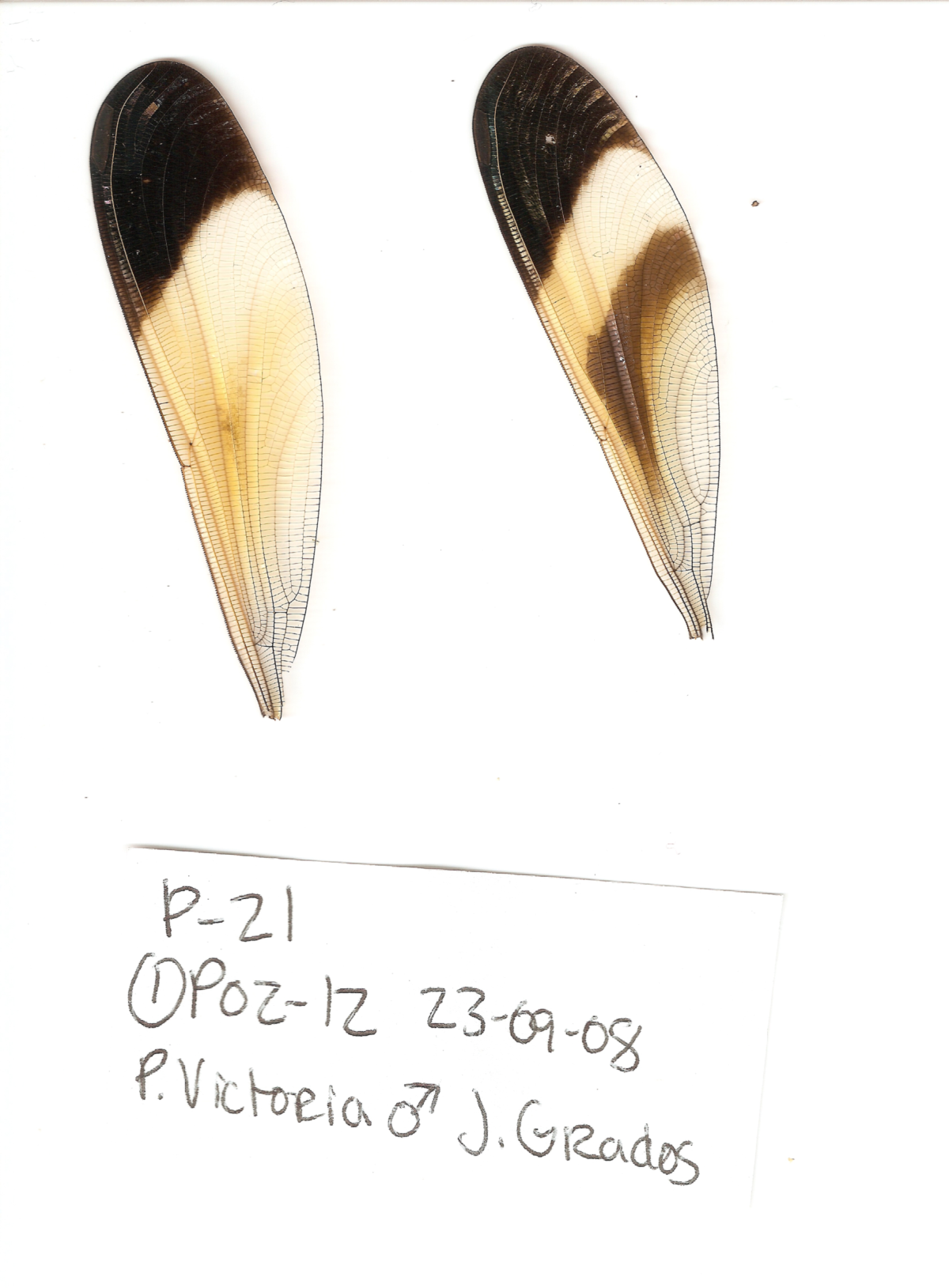

Supplement: S2 File — Compressed folder containing everything needed to run the analyses presented in this paper, including images, data, and a Mathematica notebook. (ZIP) [file pone.0125074.s002.zip › Supplementary file/images/scan0011.png]

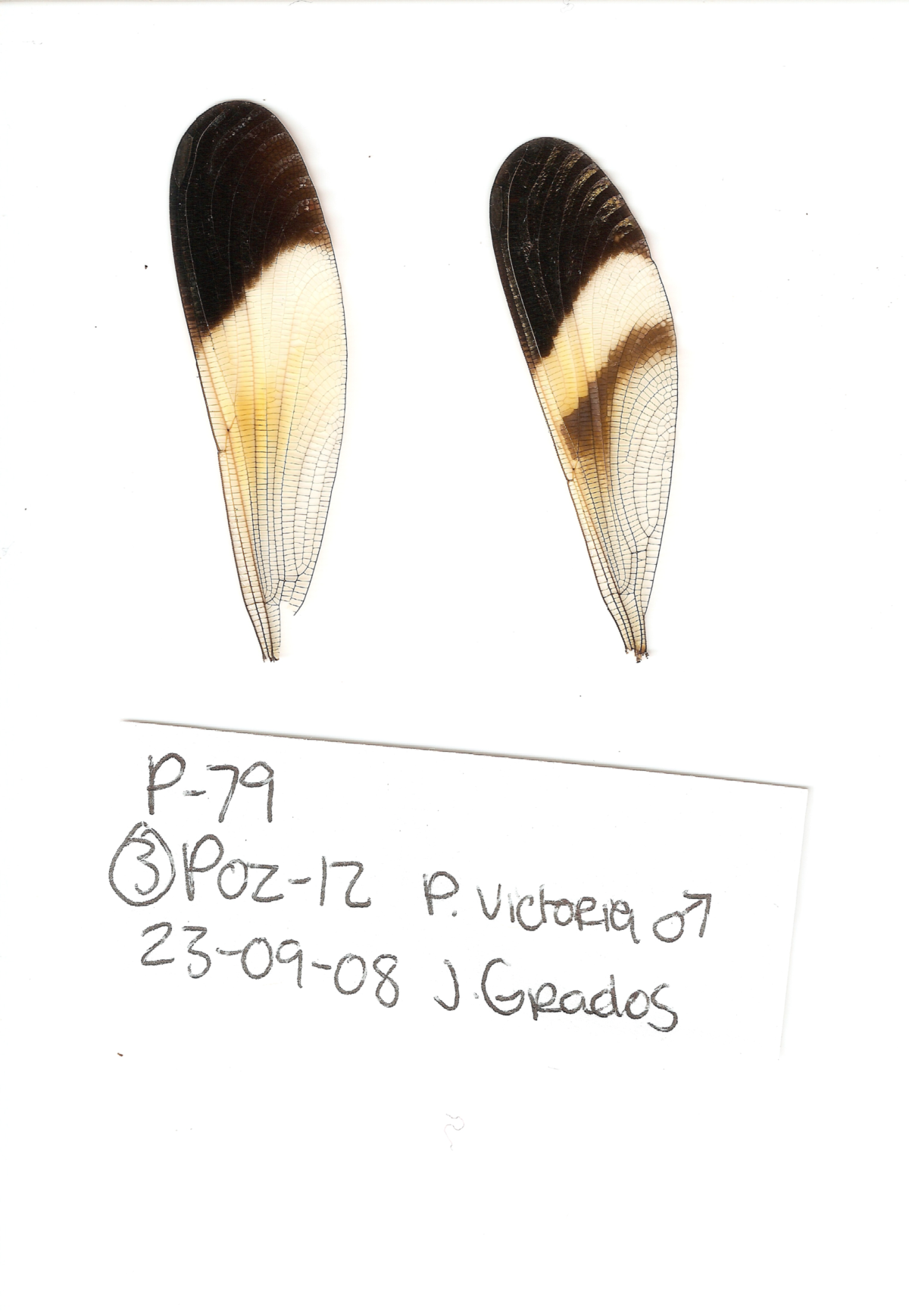

Supplement: S2 File — Compressed folder containing everything needed to run the analyses presented in this paper, including images, data, and a Mathematica notebook. (ZIP) [file pone.0125074.s002.zip › Supplementary file/images/scan0012.png]

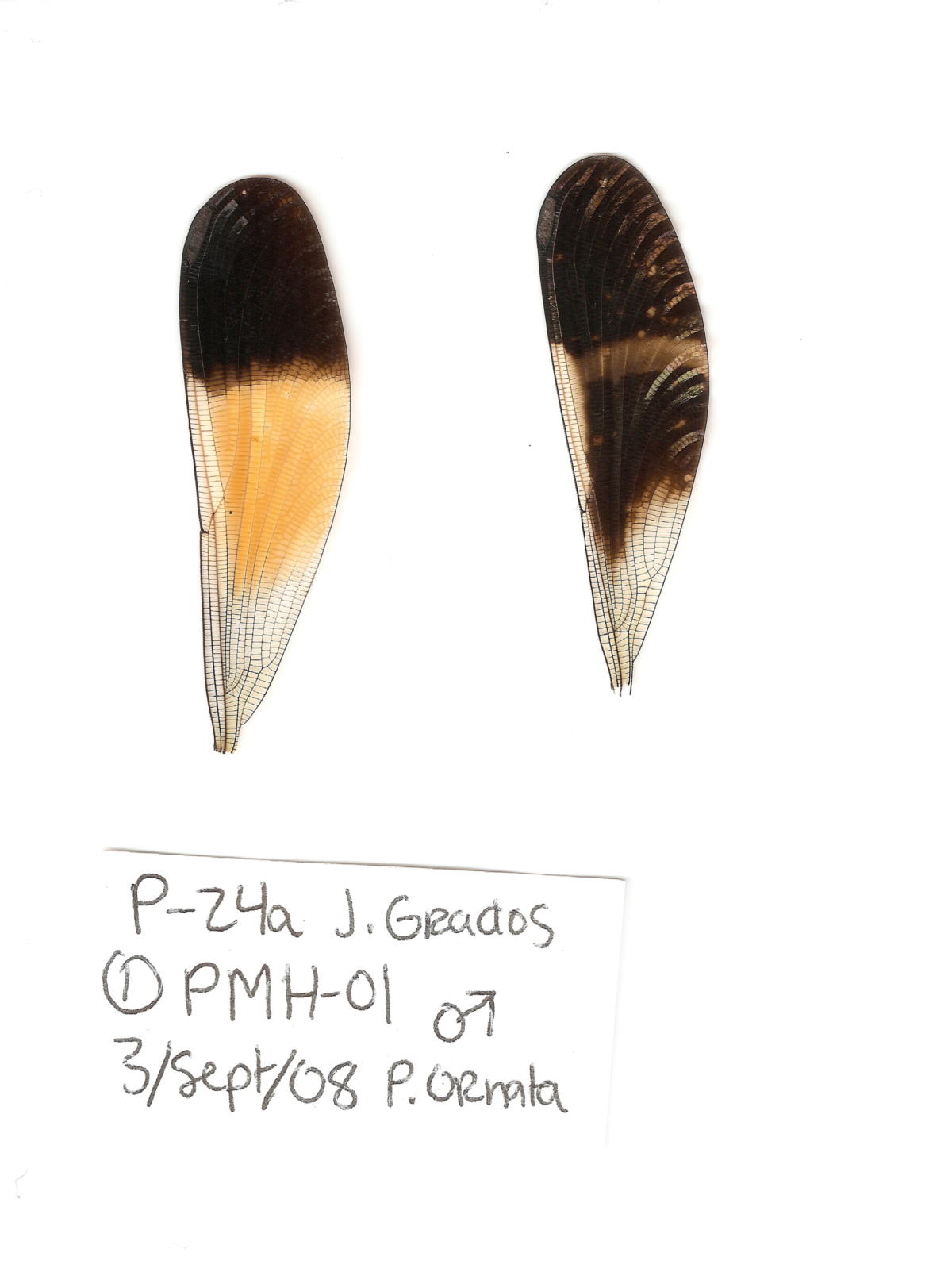

Supplement: S2 File — Compressed folder containing everything needed to run the analyses presented in this paper, including images, data, and a Mathematica notebook. (ZIP) [file pone.0125074.s002.zip › Supplementary file/images/scan0013.png]

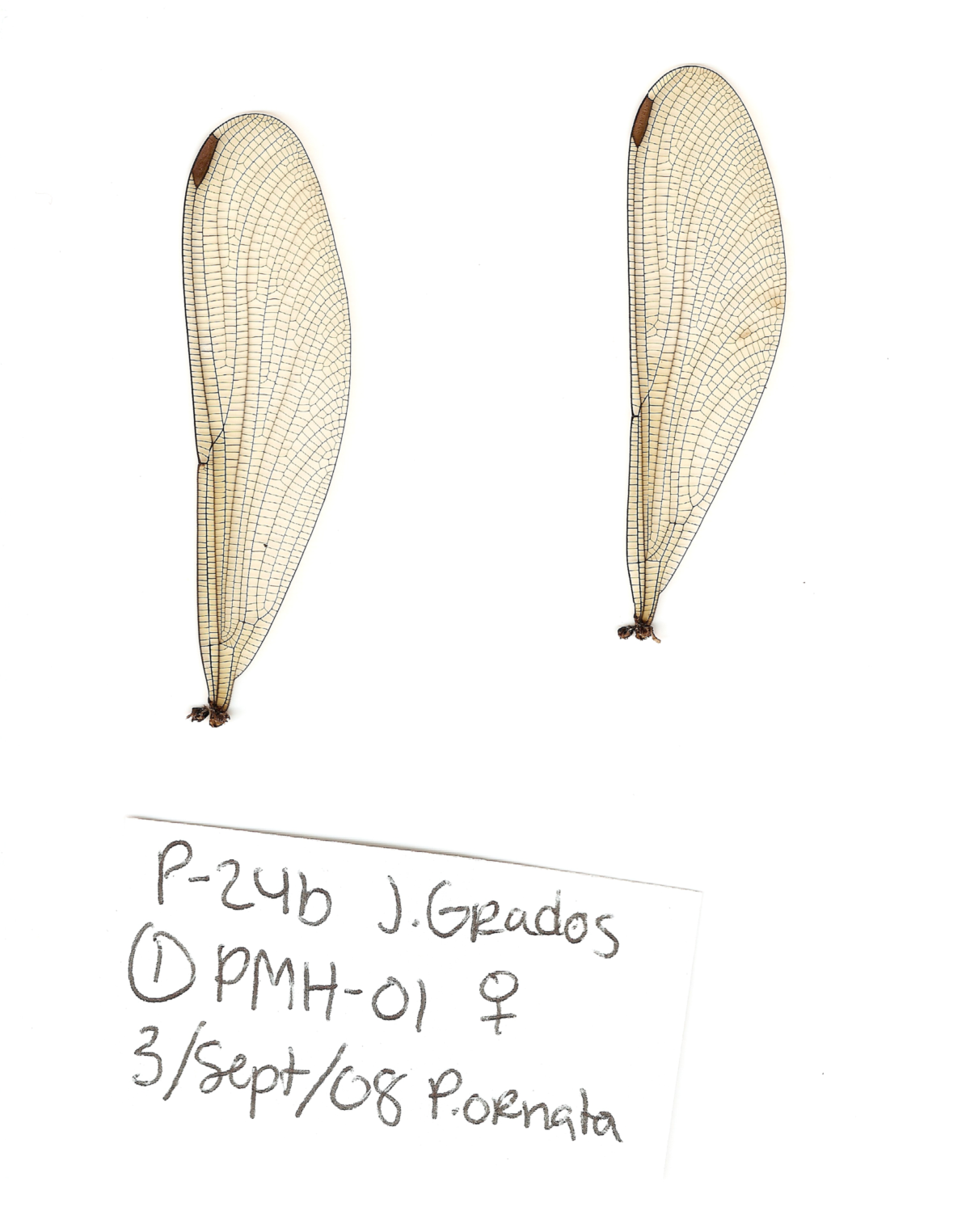

Supplement: S2 File — Compressed folder containing everything needed to run the analyses presented in this paper, including images, data, and a Mathematica notebook. (ZIP) [file pone.0125074.s002.zip › Supplementary file/images/scan0014.png]

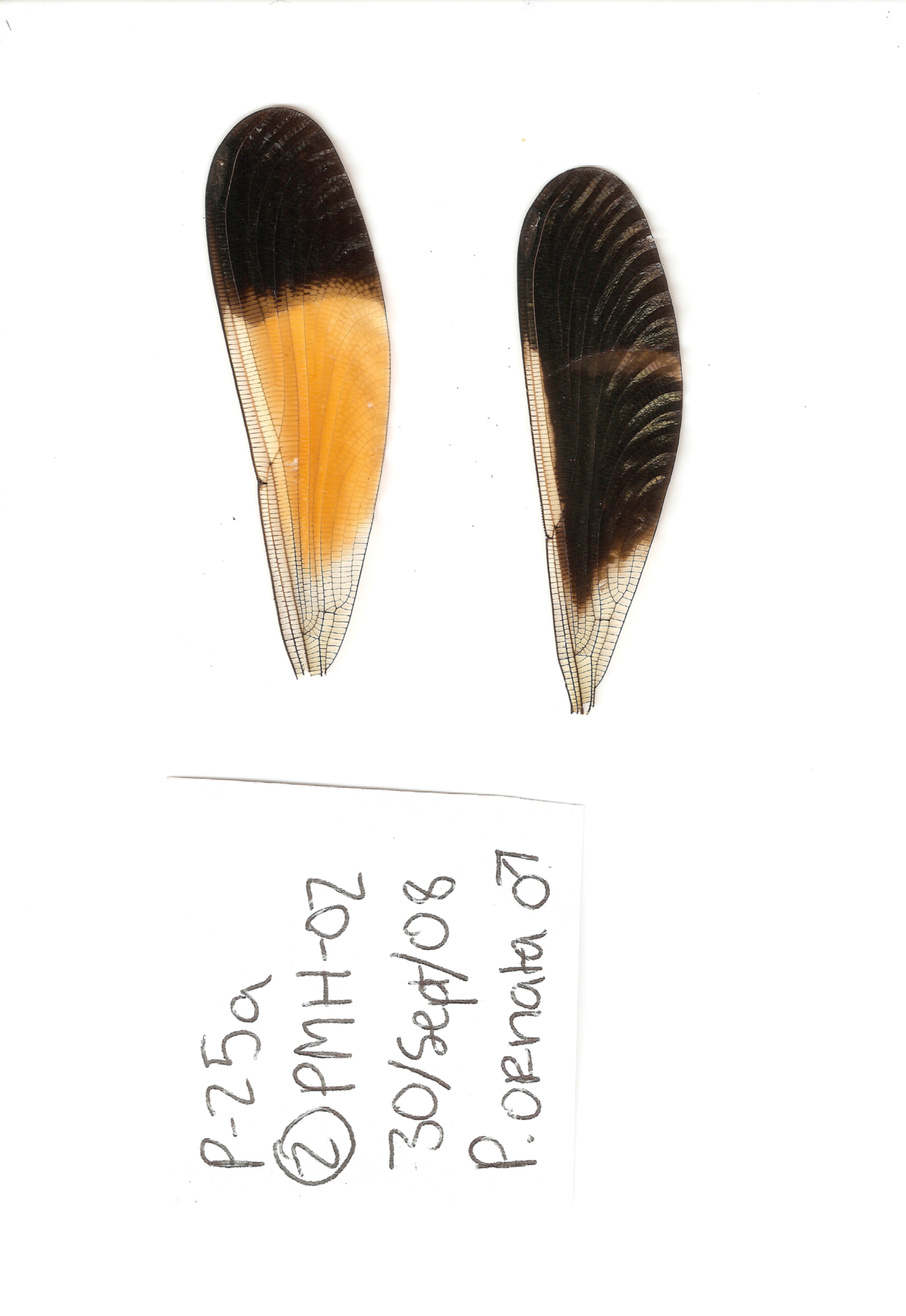

Supplement: S2 File — Compressed folder containing everything needed to run the analyses presented in this paper, including images, data, and a Mathematica notebook. (ZIP) [file pone.0125074.s002.zip › Supplementary file/images/scan0015.png]

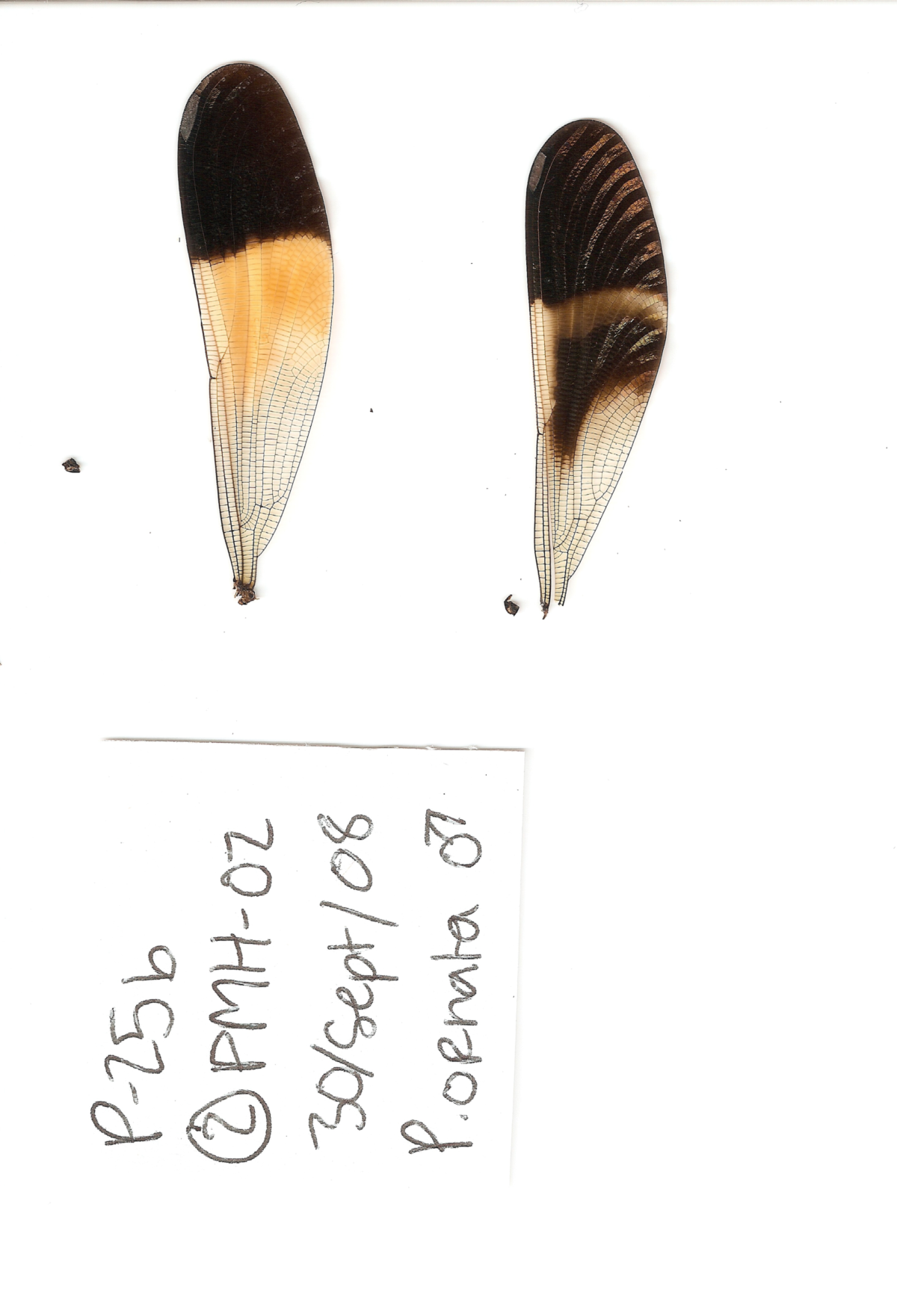

Supplement: S2 File — Compressed folder containing everything needed to run the analyses presented in this paper, including images, data, and a Mathematica notebook. (ZIP) [file pone.0125074.s002.zip › Supplementary file/images/scan0016.png]

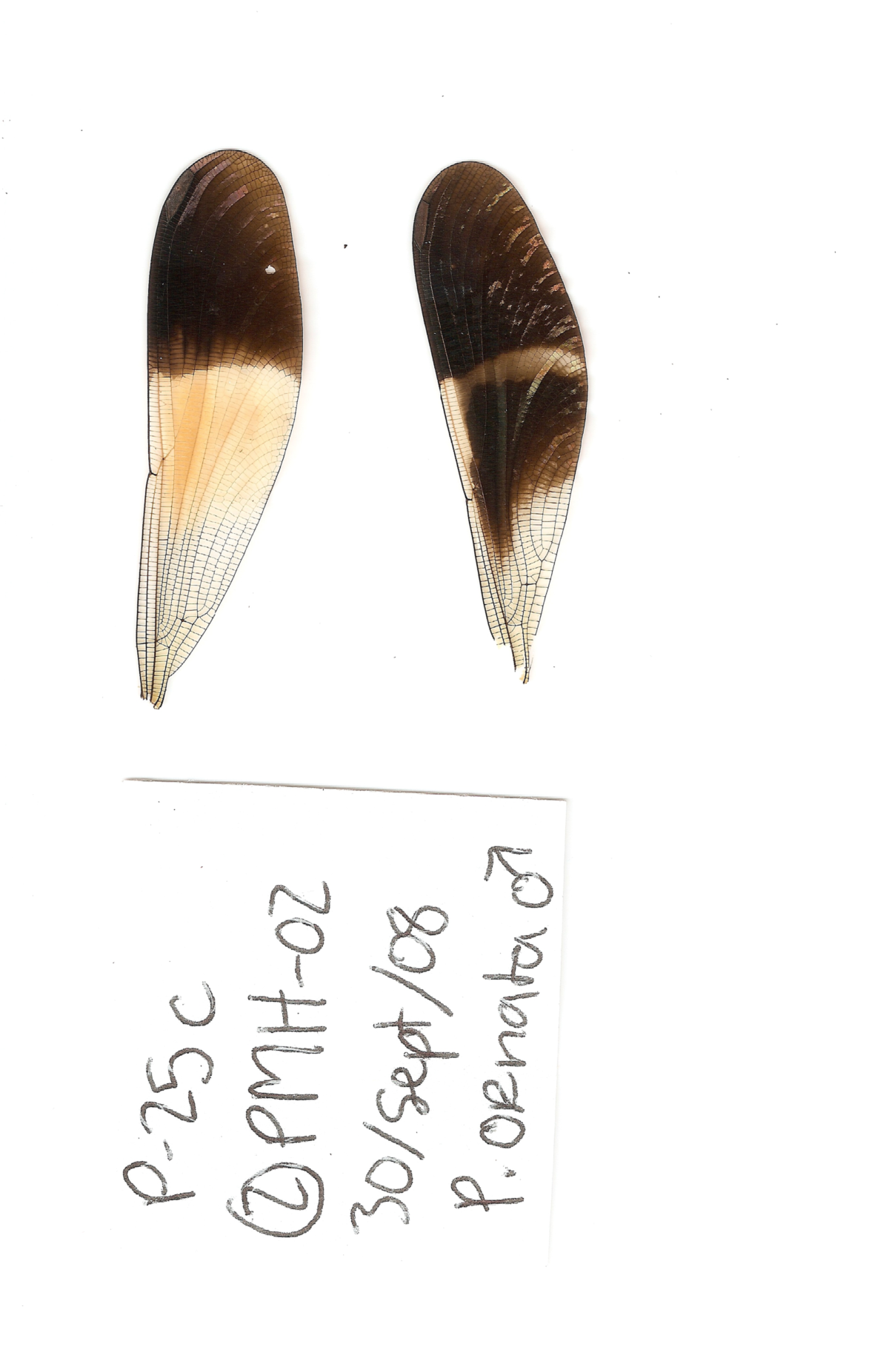

Supplement: S2 File — Compressed folder containing everything needed to run the analyses presented in this paper, including images, data, and a Mathematica notebook. (ZIP) [file pone.0125074.s002.zip › Supplementary file/images/scan0017.png]

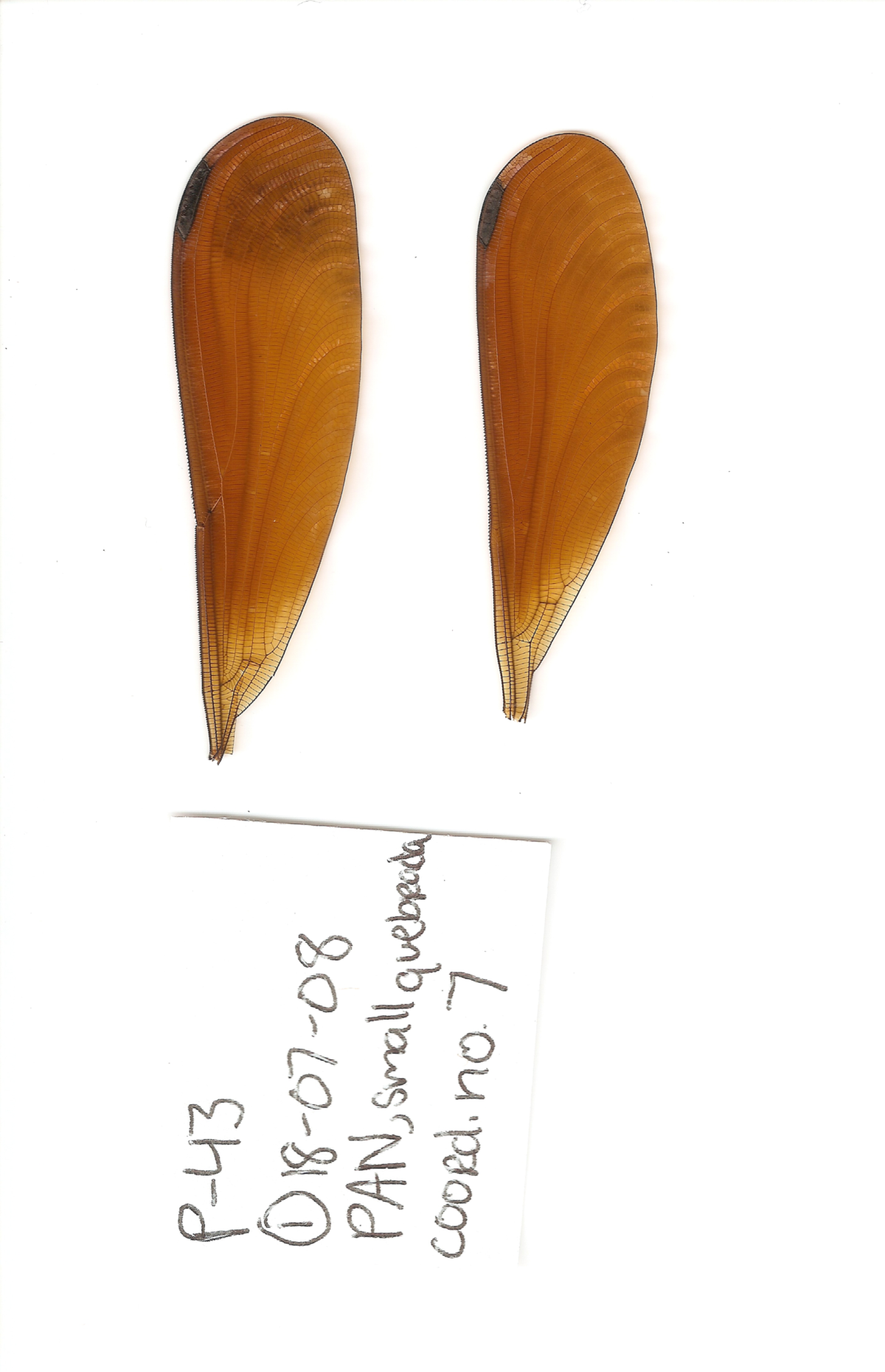

Supplement: S2 File — Compressed folder containing everything needed to run the analyses presented in this paper, including images, data, and a Mathematica notebook. (ZIP) [file pone.0125074.s002.zip › Supplementary file/images/scan0018.png]

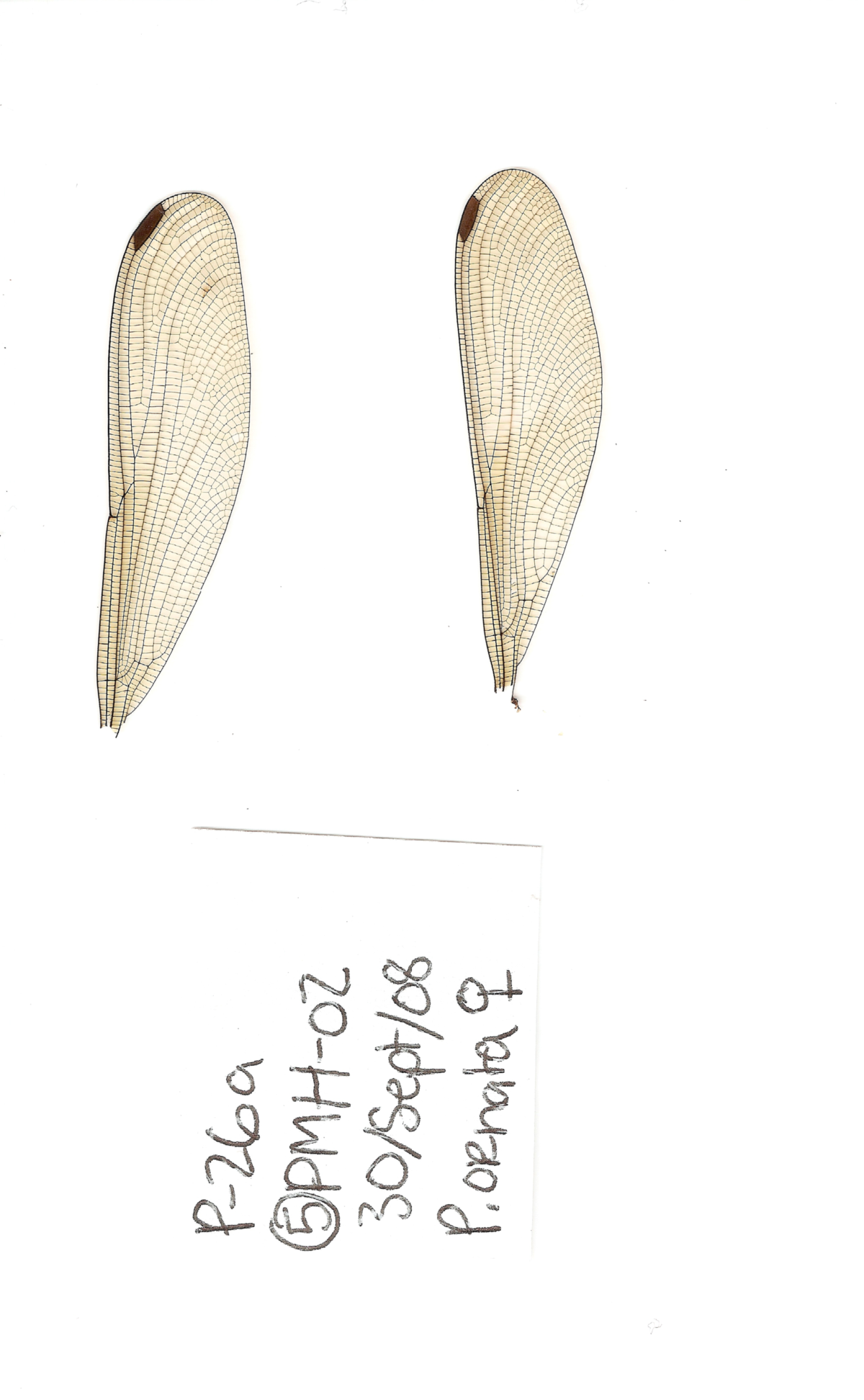

Supplement: S2 File — Compressed folder containing everything needed to run the analyses presented in this paper, including images, data, and a Mathematica notebook. (ZIP) [file pone.0125074.s002.zip › Supplementary file/images/scan0019.png]

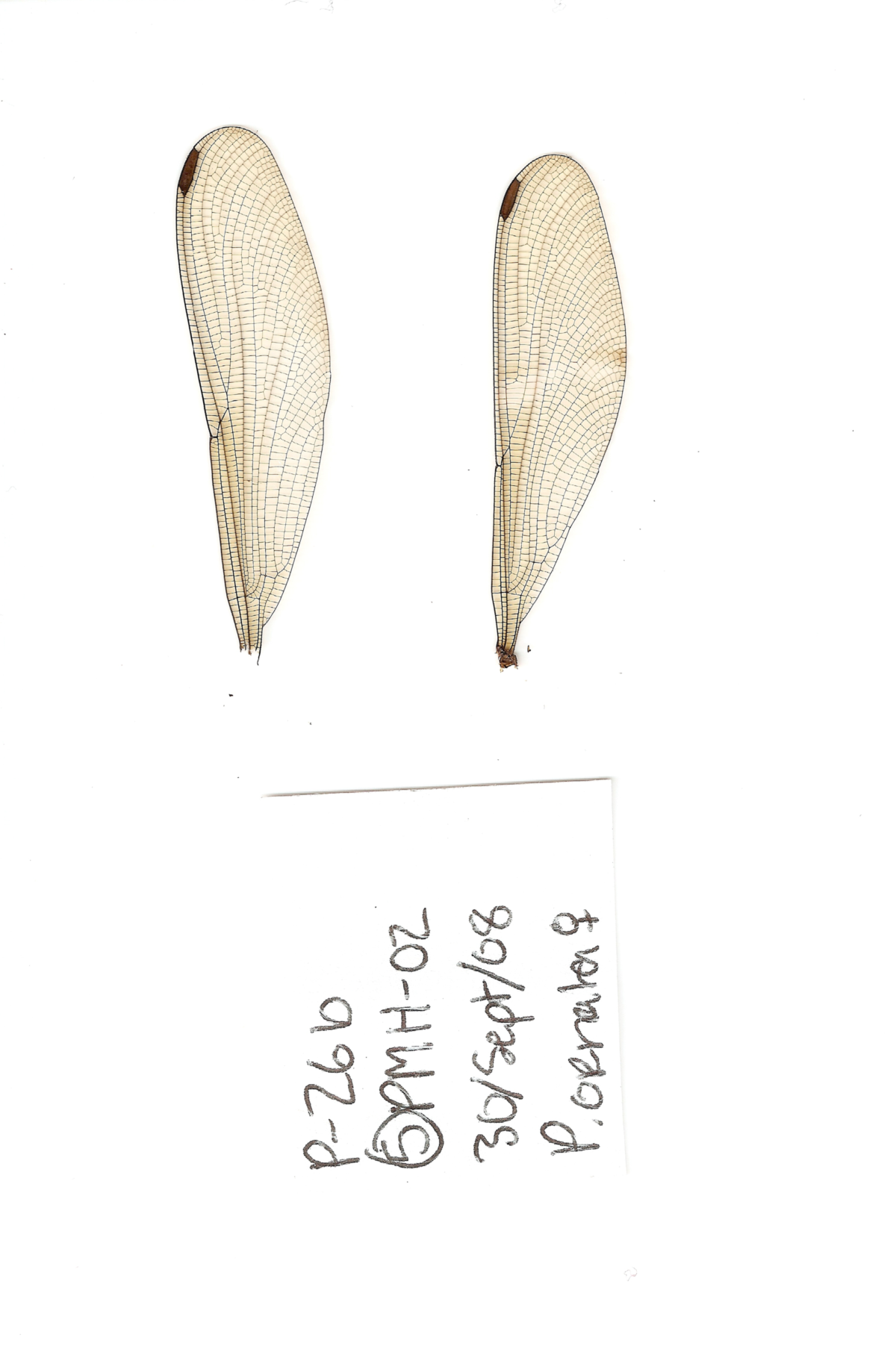

Supplement: S2 File — Compressed folder containing everything needed to run the analyses presented in this paper, including images, data, and a Mathematica notebook. (ZIP) [file pone.0125074.s002.zip › Supplementary file/images/scan0020.png]

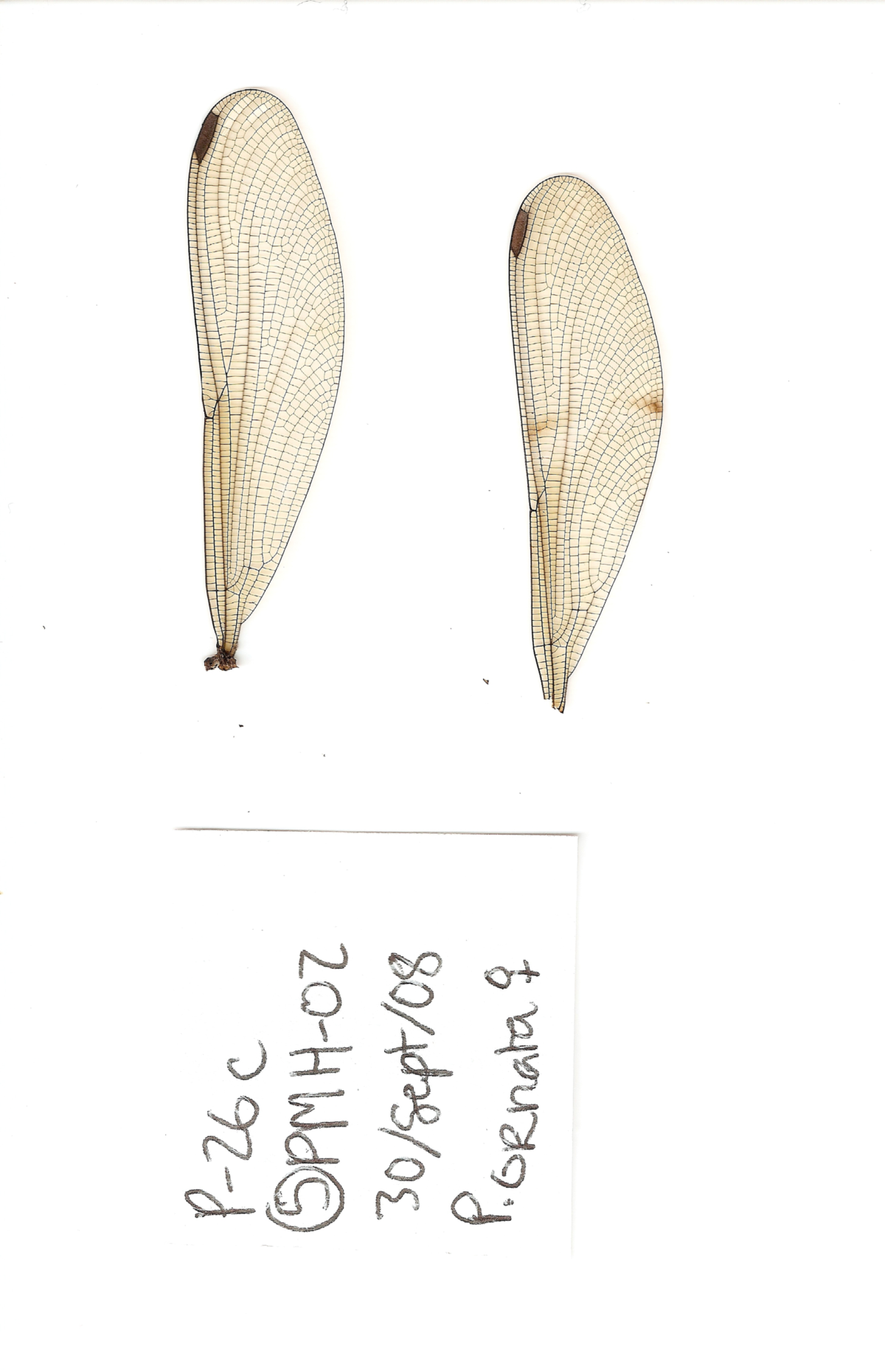

Supplement: S2 File — Compressed folder containing everything needed to run the analyses presented in this paper, including images, data, and a Mathematica notebook. (ZIP) [file pone.0125074.s002.zip › Supplementary file/images/scan0021.png]

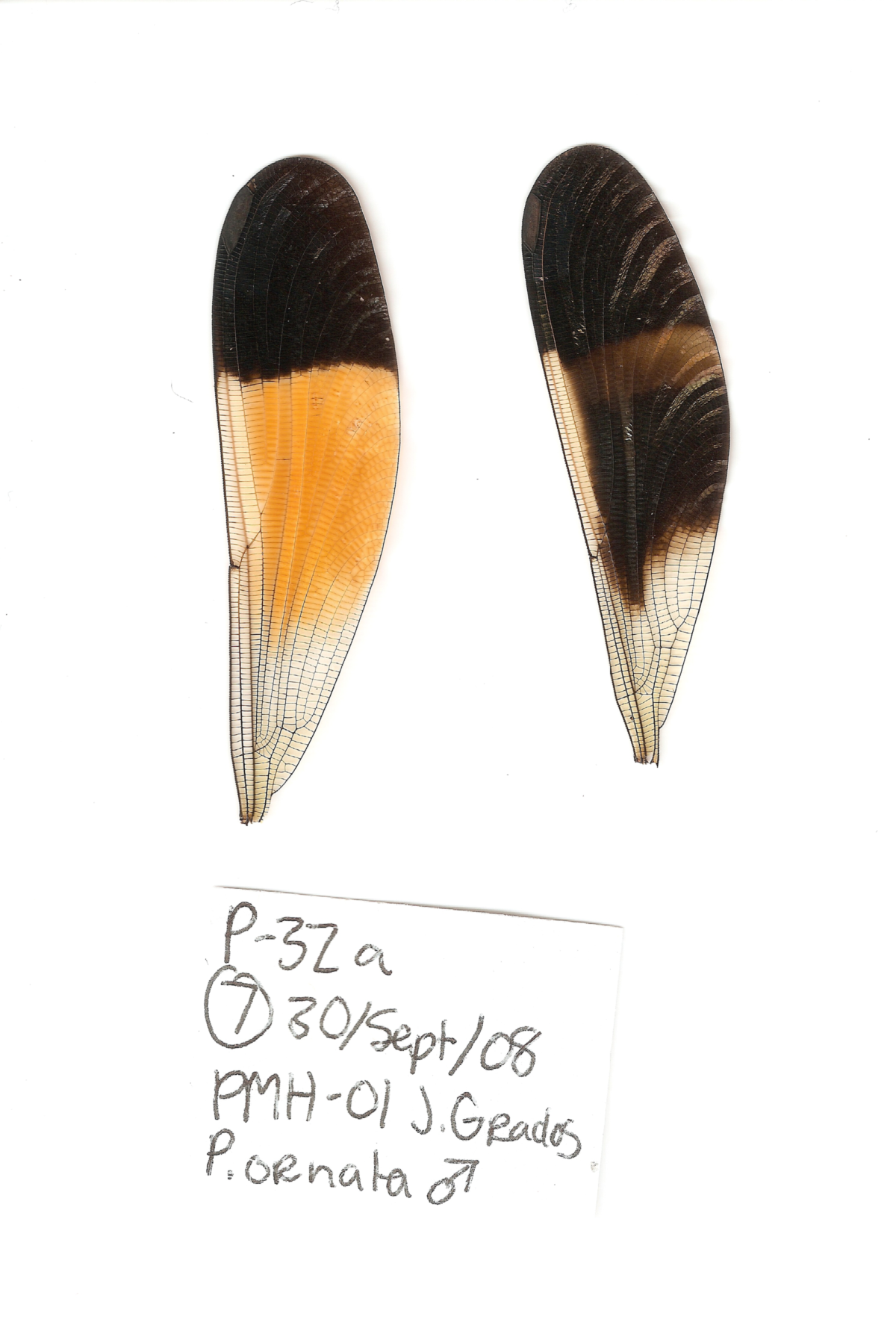

Supplement: S2 File — Compressed folder containing everything needed to run the analyses presented in this paper, including images, data, and a Mathematica notebook. (ZIP) [file pone.0125074.s002.zip › Supplementary file/images/scan0022.png]

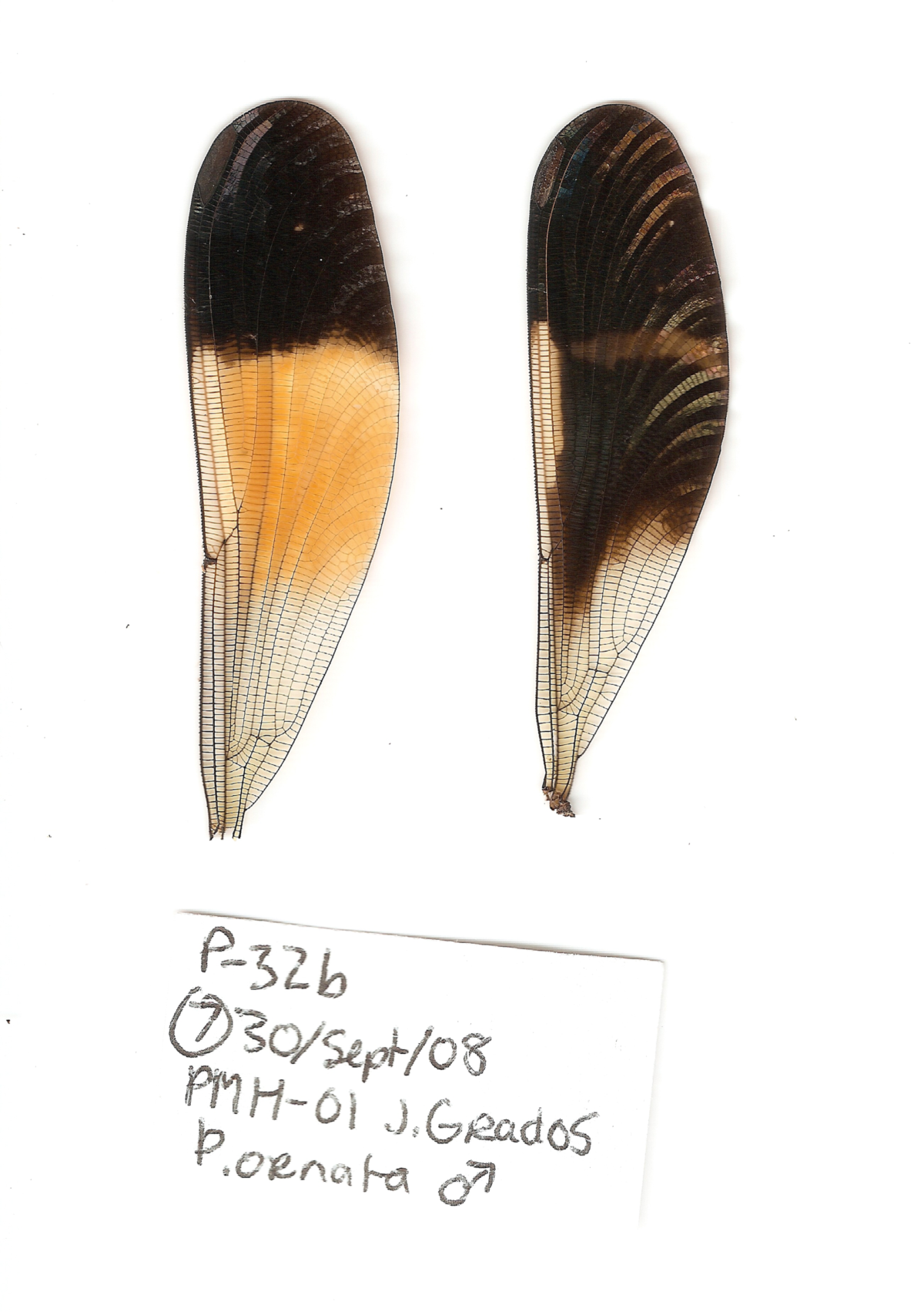

Supplement: S2 File — Compressed folder containing everything needed to run the analyses presented in this paper, including images, data, and a Mathematica notebook. (ZIP) [file pone.0125074.s002.zip › Supplementary file/images/scan0023.png]

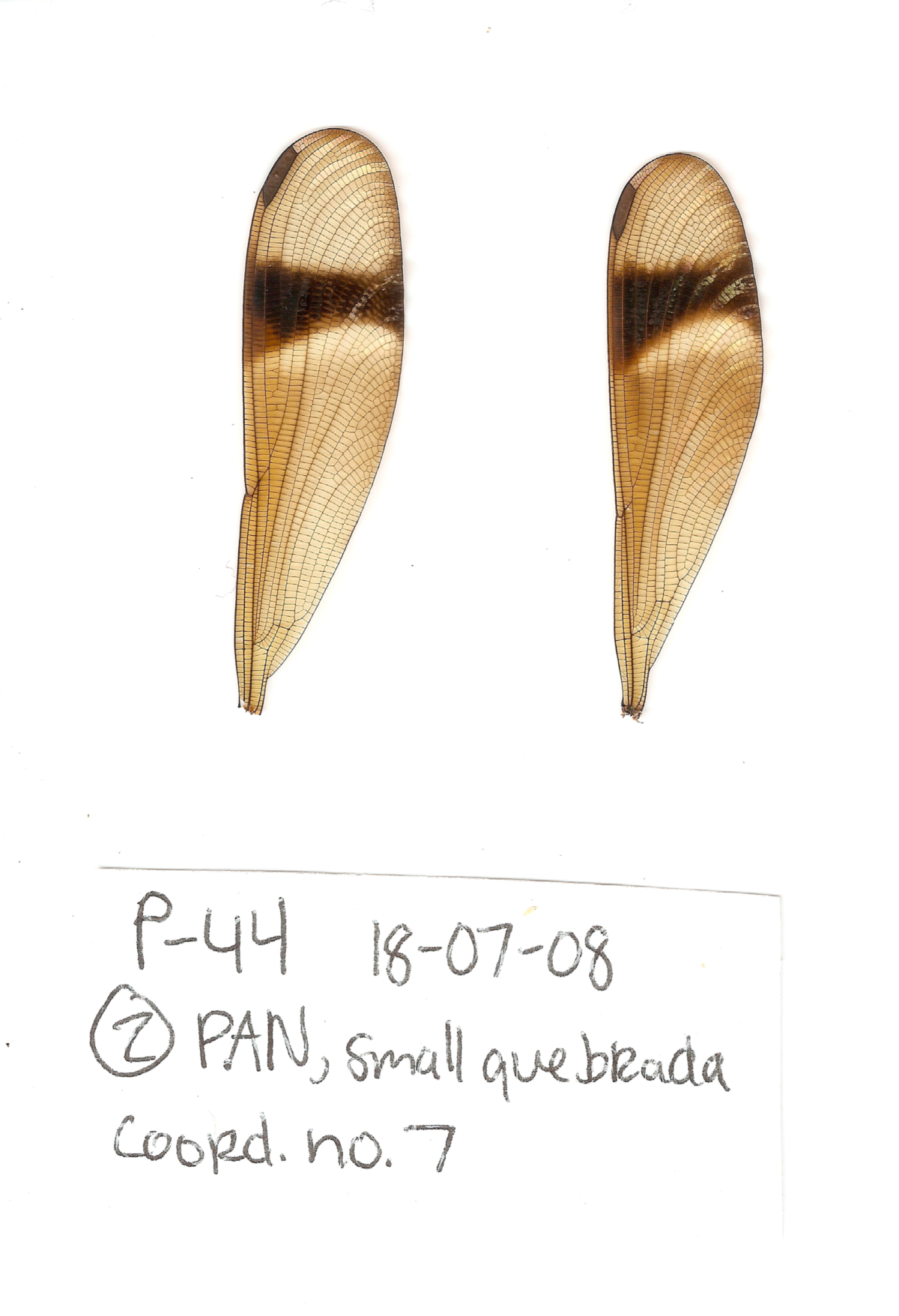

Supplement: S2 File — Compressed folder containing everything needed to run the analyses presented in this paper, including images, data, and a Mathematica notebook. (ZIP) [file pone.0125074.s002.zip › Supplementary file/images/scan0024.png]

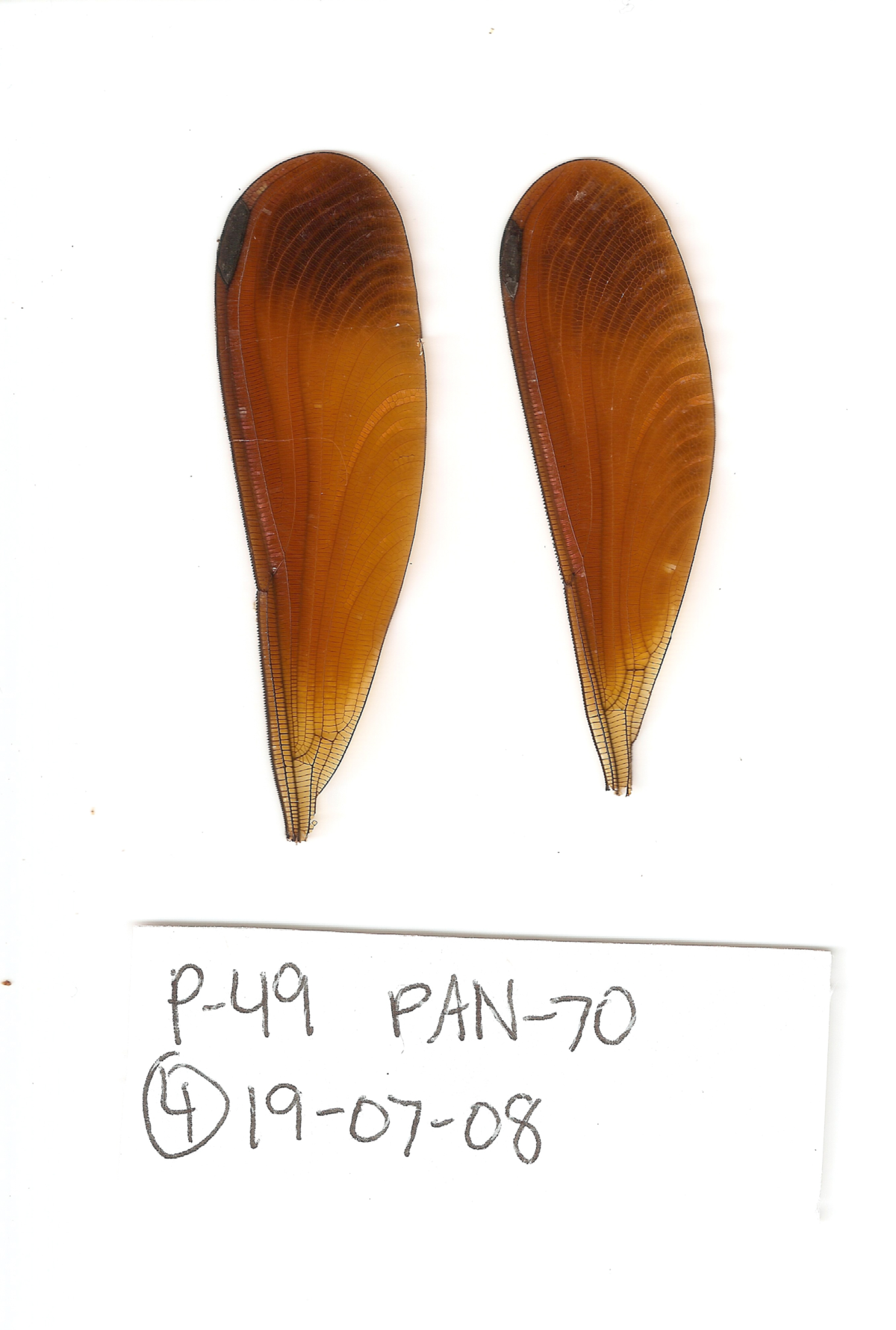

Supplement: S2 File — Compressed folder containing everything needed to run the analyses presented in this paper, including images, data, and a Mathematica notebook. (ZIP) [file pone.0125074.s002.zip › Supplementary file/images/scan0025.png]

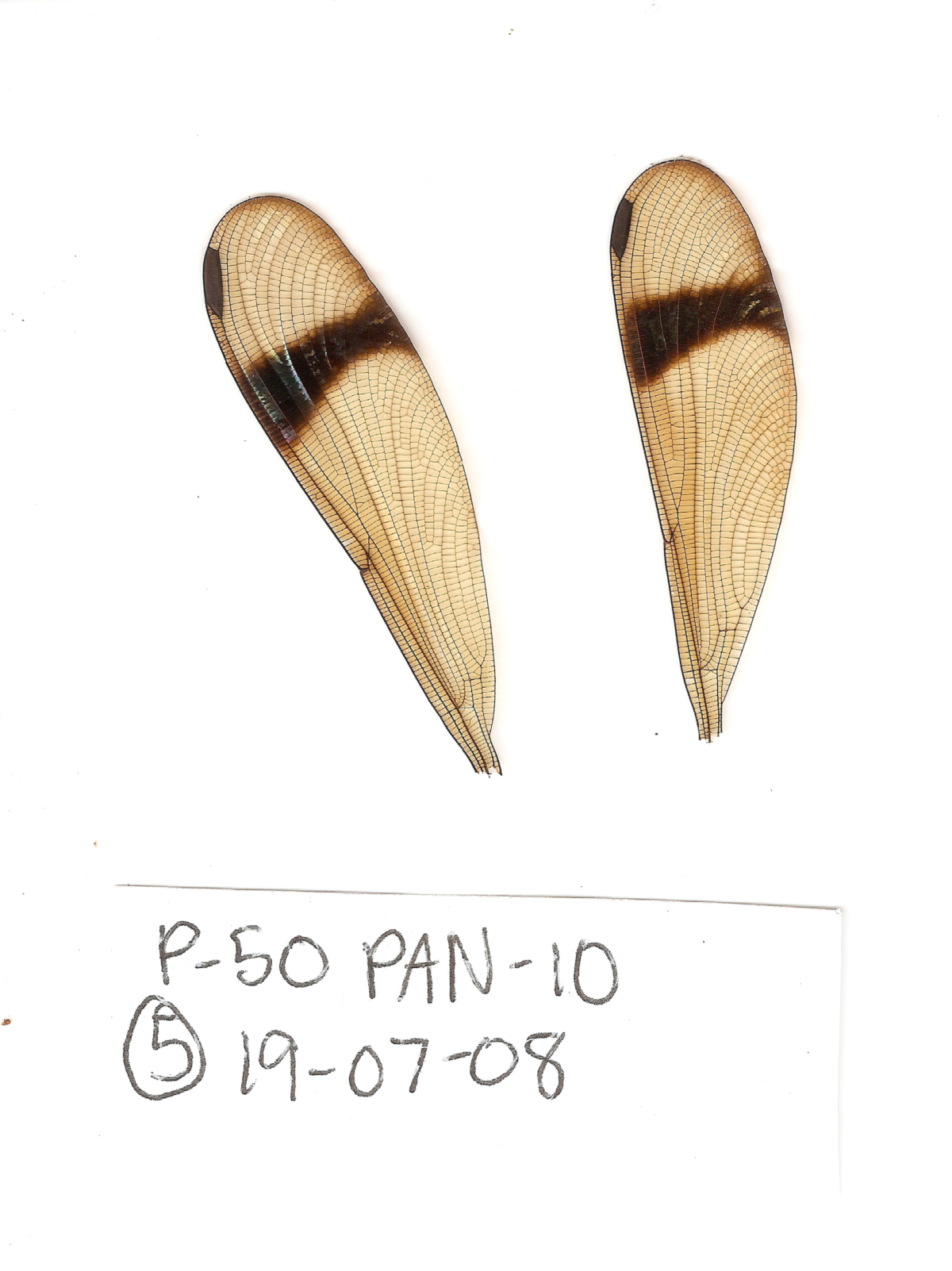

Supplement: S2 File — Compressed folder containing everything needed to run the analyses presented in this paper, including images, data, and a Mathematica notebook. (ZIP) [file pone.0125074.s002.zip › Supplementary file/images/scan0026.png]

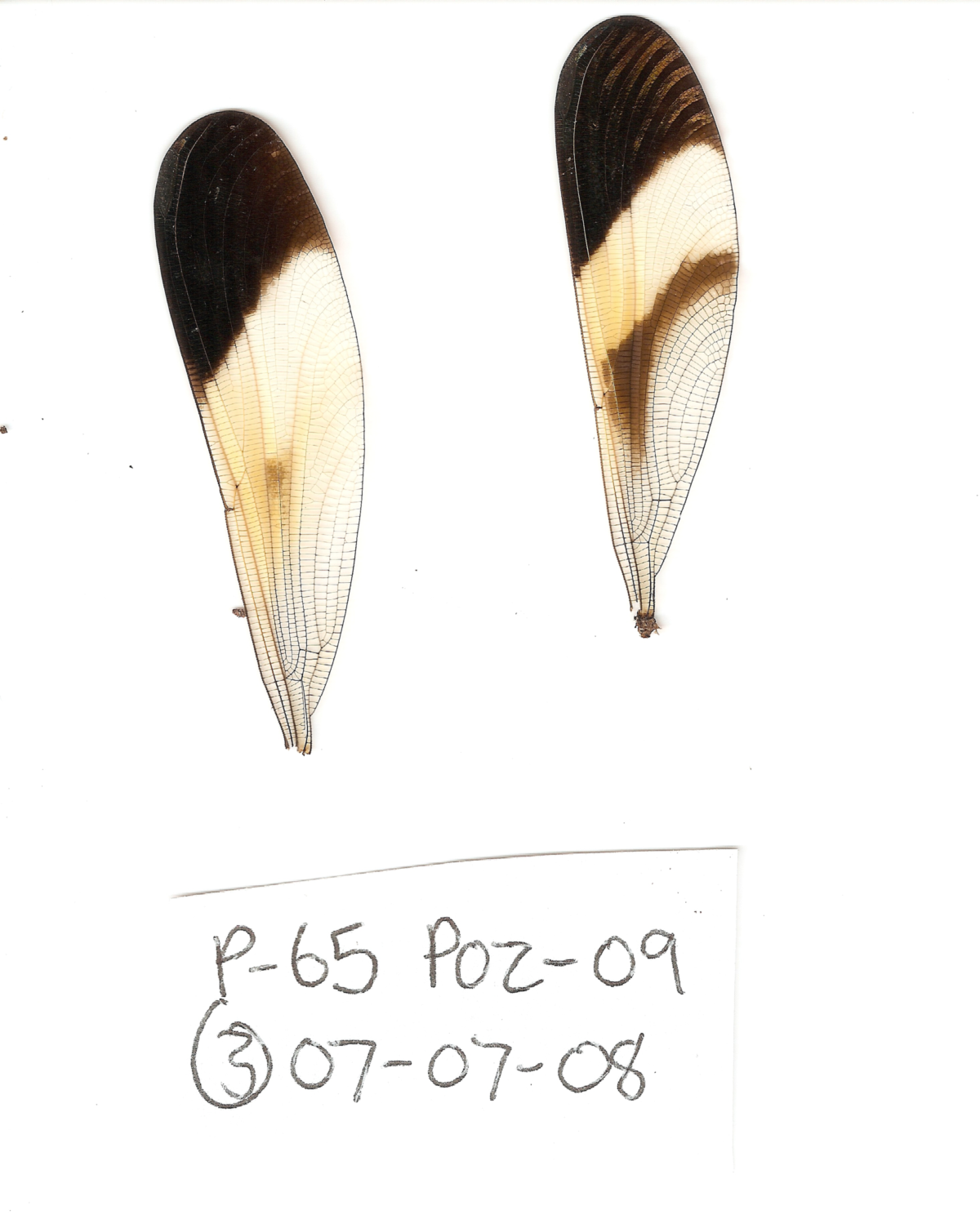

Supplement: S2 File — Compressed folder containing everything needed to run the analyses presented in this paper, including images, data, and a Mathematica notebook. (ZIP) [file pone.0125074.s002.zip › Supplementary file/images/scan0027.png]

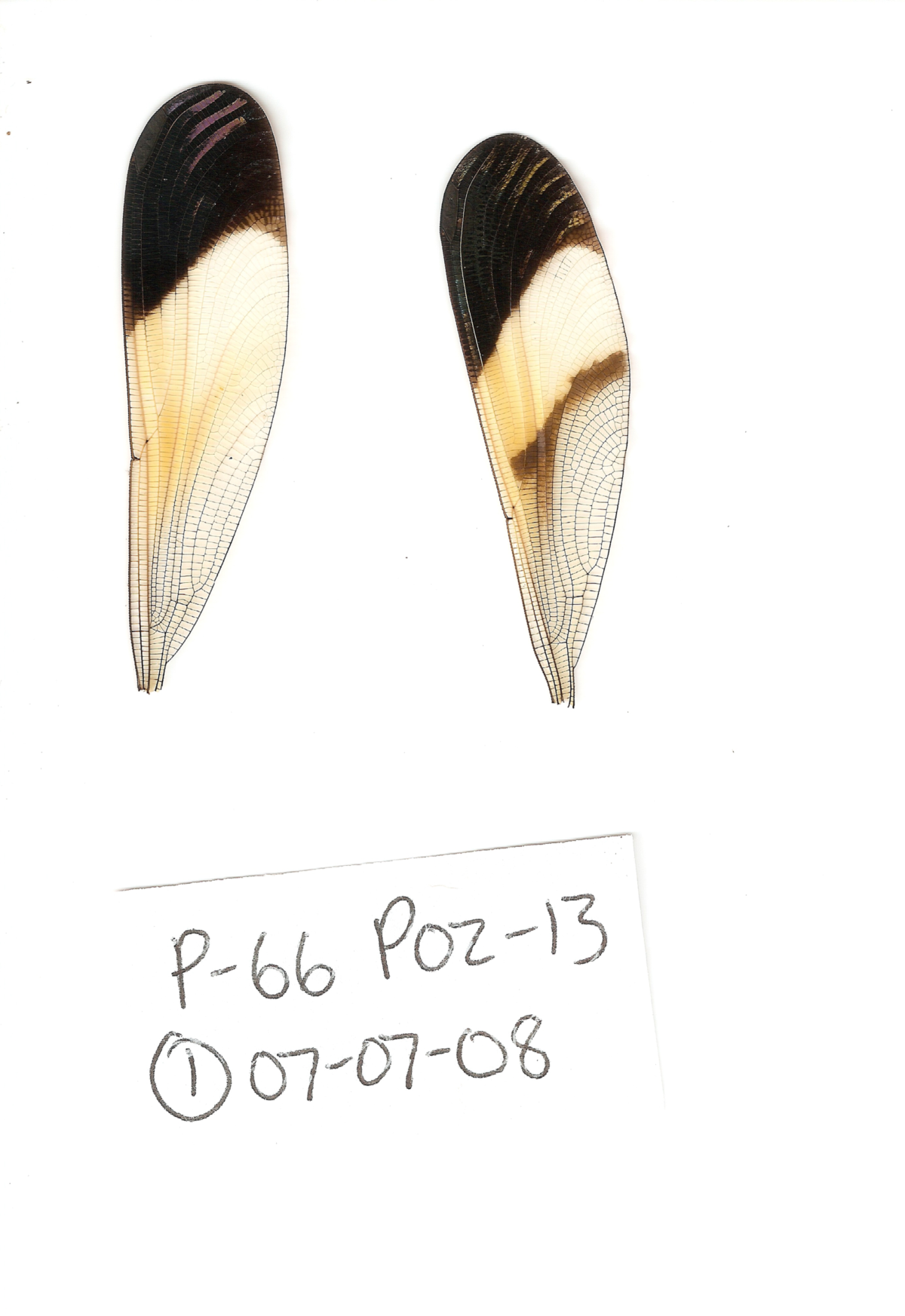

Supplement: S2 File — Compressed folder containing everything needed to run the analyses presented in this paper, including images, data, and a Mathematica notebook. (ZIP) [file pone.0125074.s002.zip › Supplementary file/images/scan0028.png]

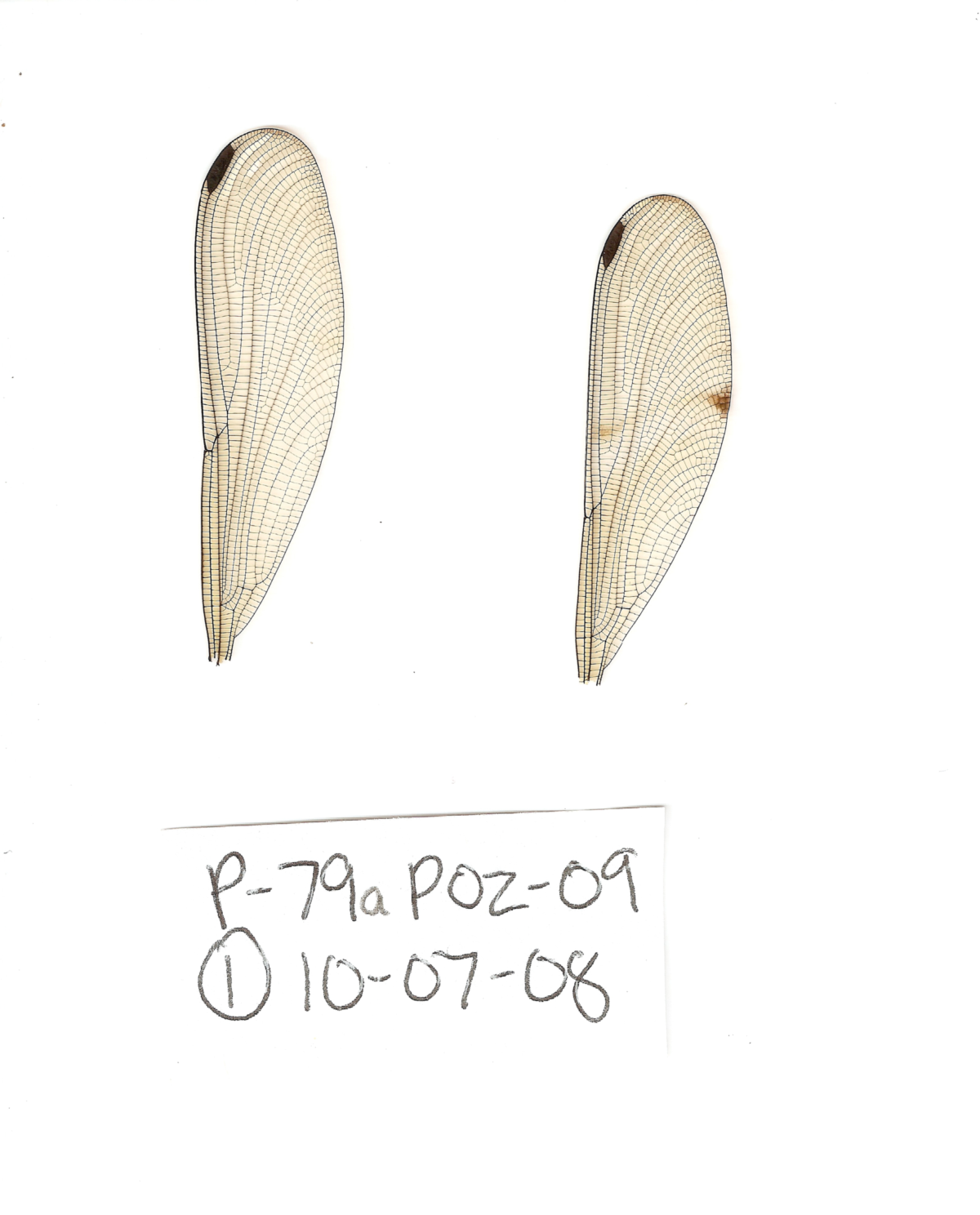

Supplement: S2 File — Compressed folder containing everything needed to run the analyses presented in this paper, including images, data, and a Mathematica notebook. (ZIP) [file pone.0125074.s002.zip › Supplementary file/images/scan0030.png]

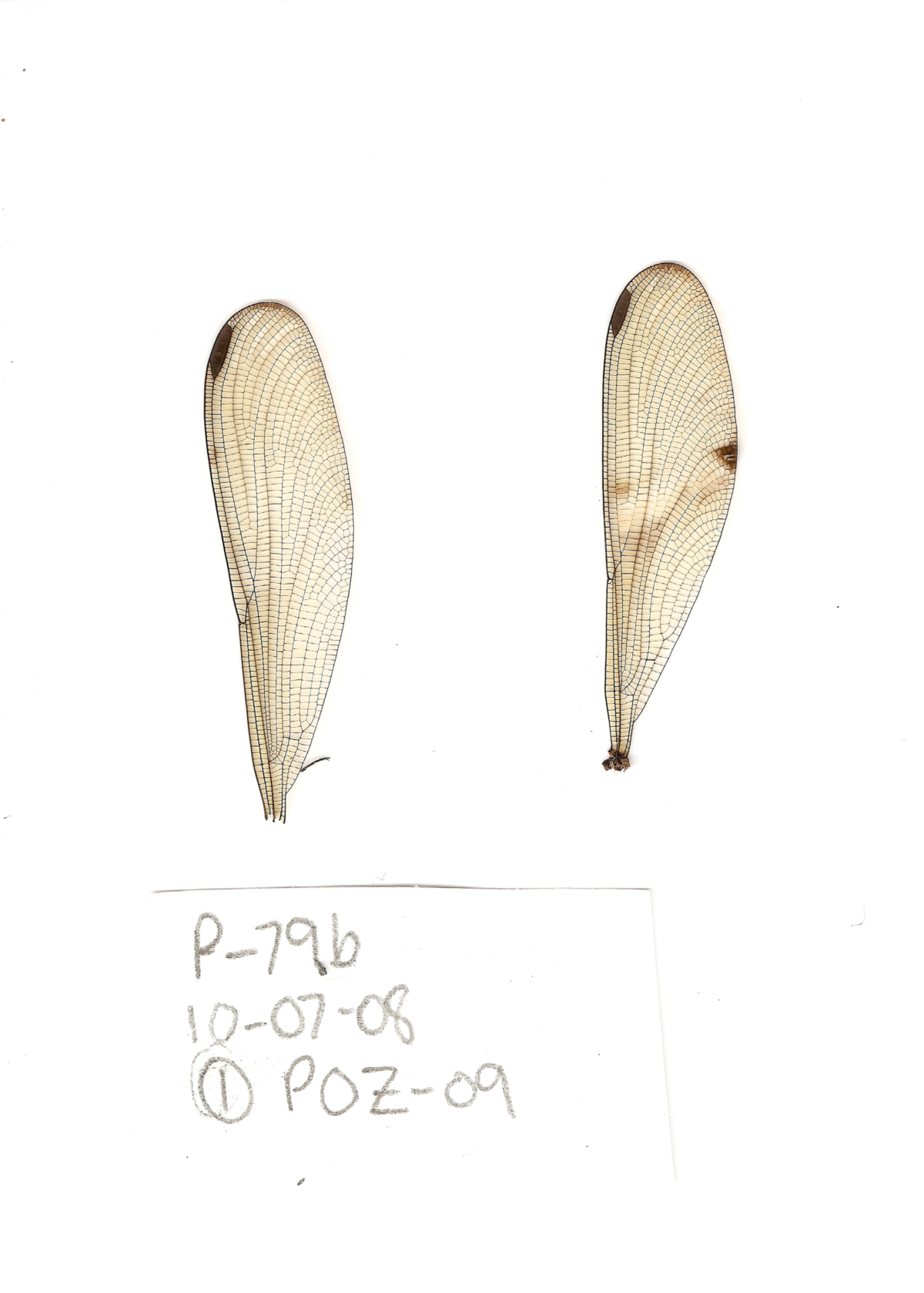

Supplement: S2 File — Compressed folder containing everything needed to run the analyses presented in this paper, including images, data, and a Mathematica notebook. (ZIP) [file pone.0125074.s002.zip › Supplementary file/images/scan0031.png]

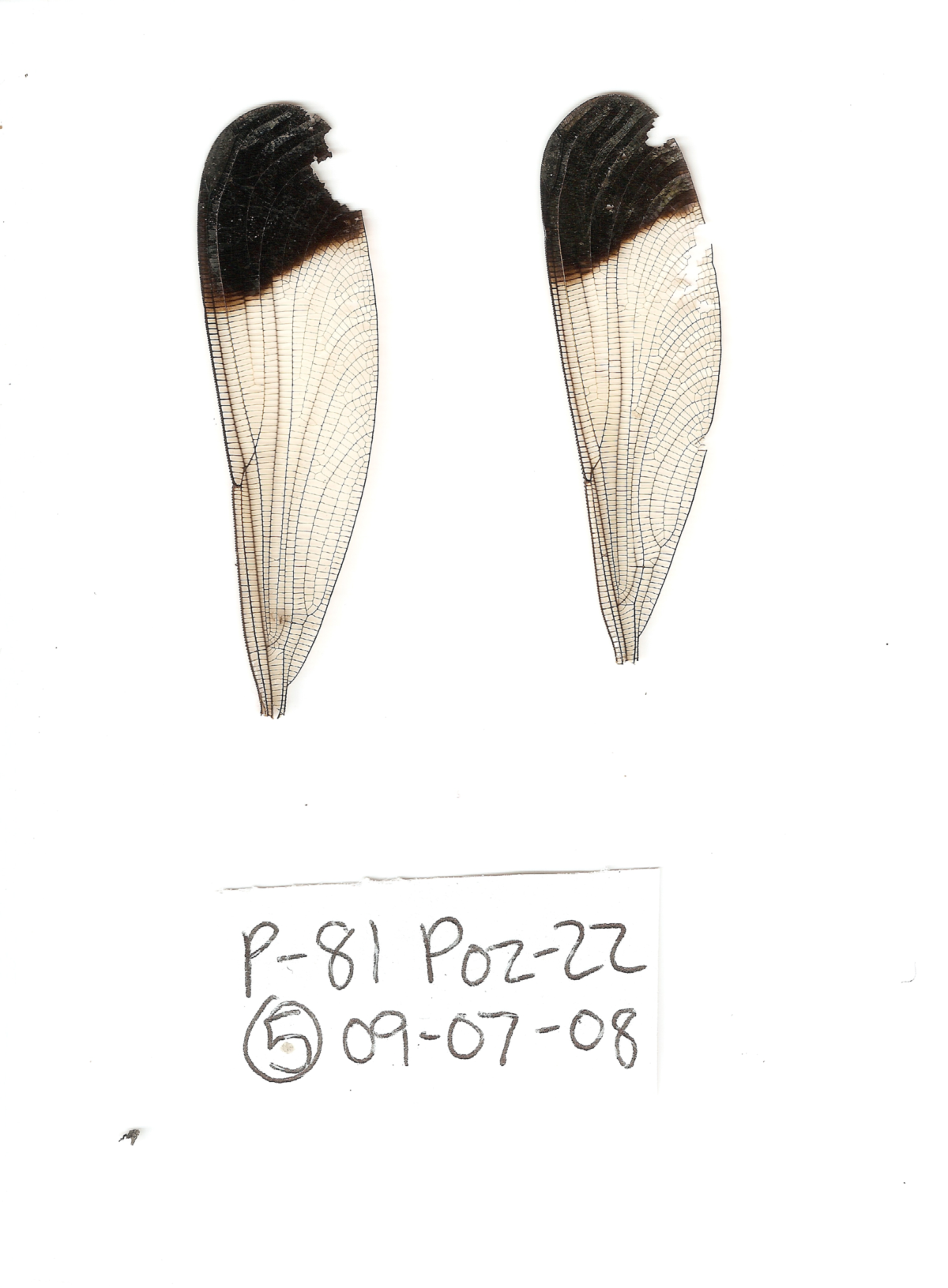

Supplement: S2 File — Compressed folder containing everything needed to run the analyses presented in this paper, including images, data, and a Mathematica notebook. (ZIP) [file pone.0125074.s002.zip › Supplementary file/images/scan0032.png]

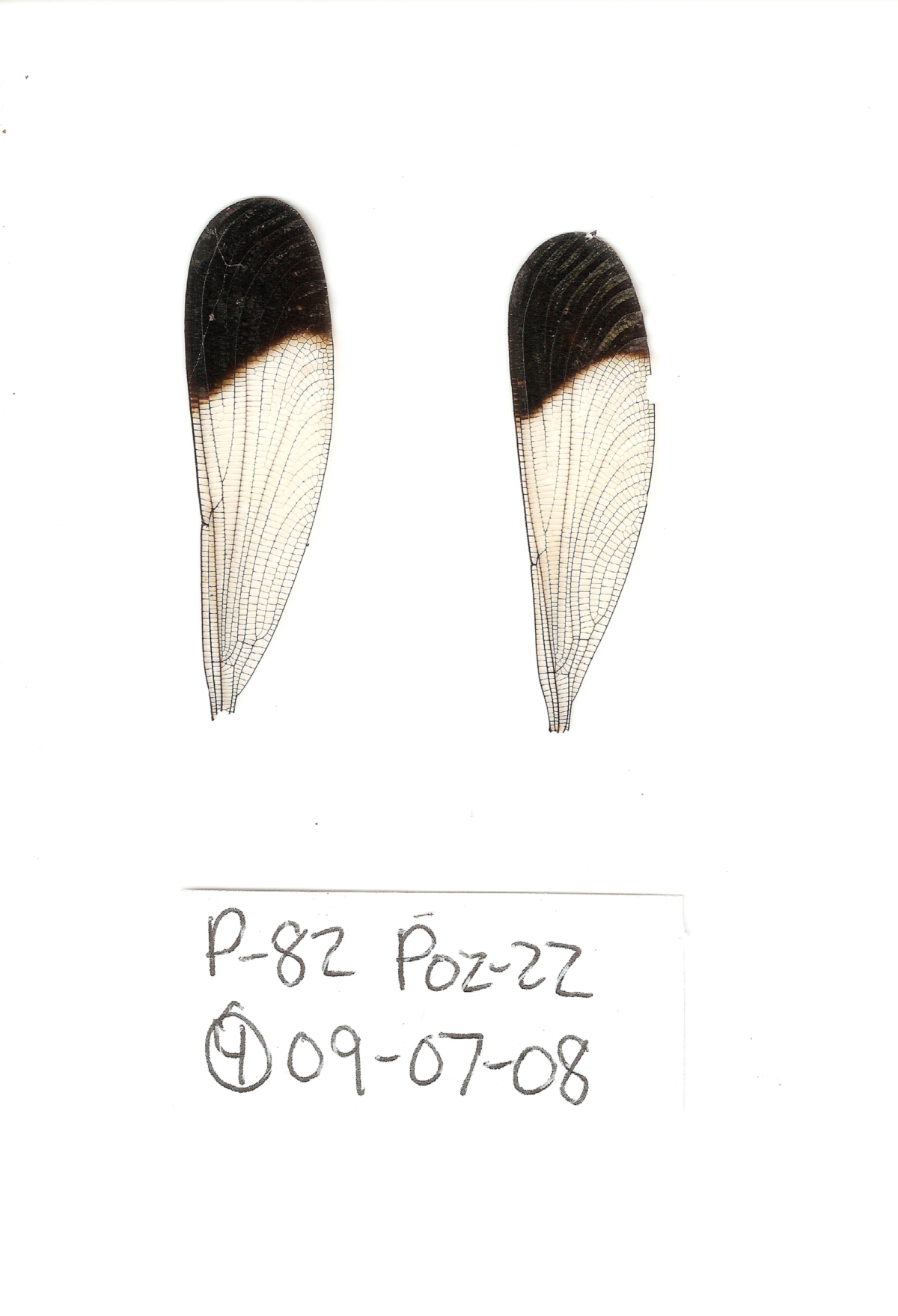

Supplement: S2 File — Compressed folder containing everything needed to run the analyses presented in this paper, including images, data, and a Mathematica notebook. (ZIP) [file pone.0125074.s002.zip › Supplementary file/images/scan0033.png]

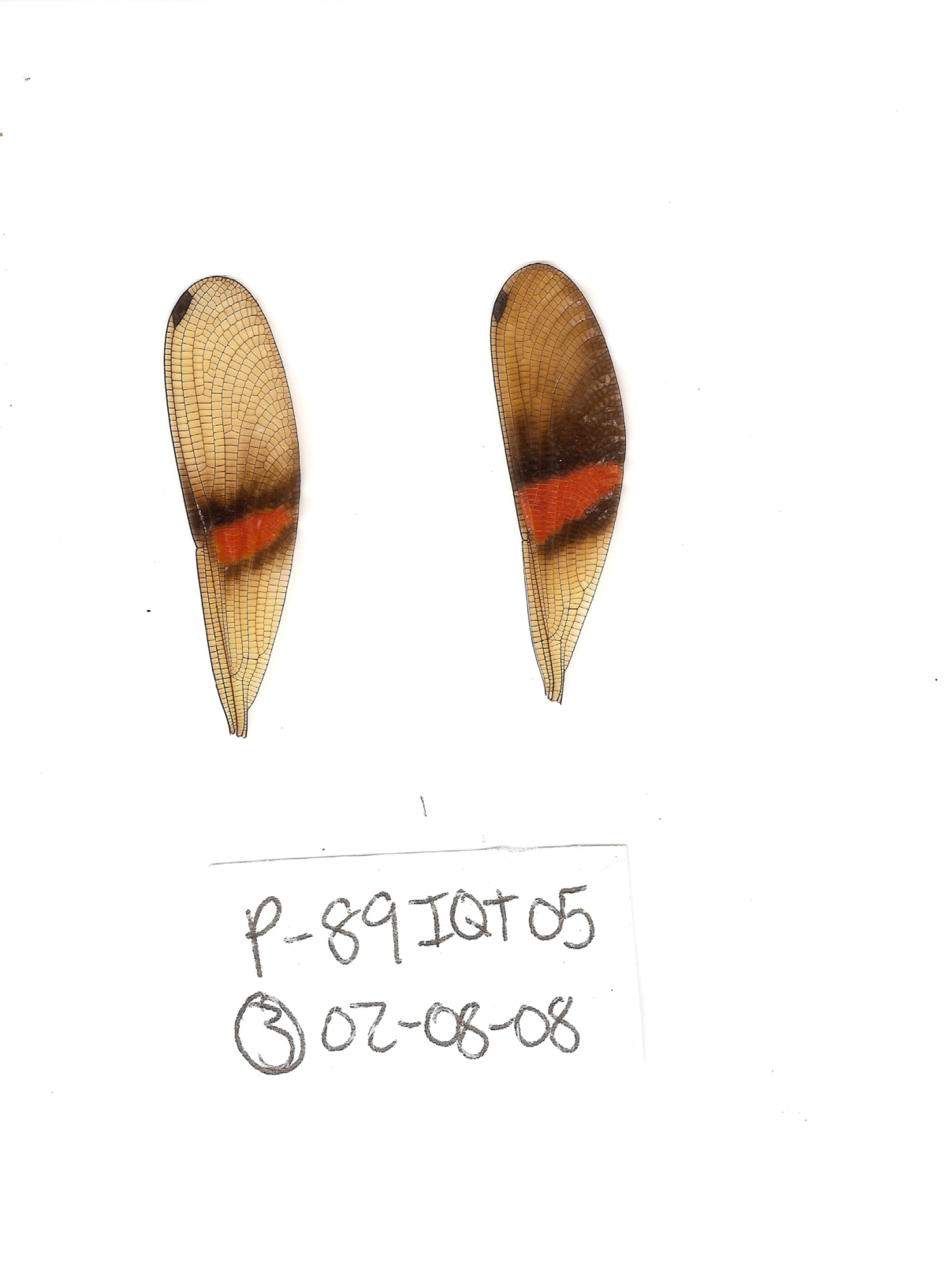

Supplement: S2 File — Compressed folder containing everything needed to run the analyses presented in this paper, including images, data, and a Mathematica notebook. (ZIP) [file pone.0125074.s002.zip › Supplementary file/images/scan0034.png]

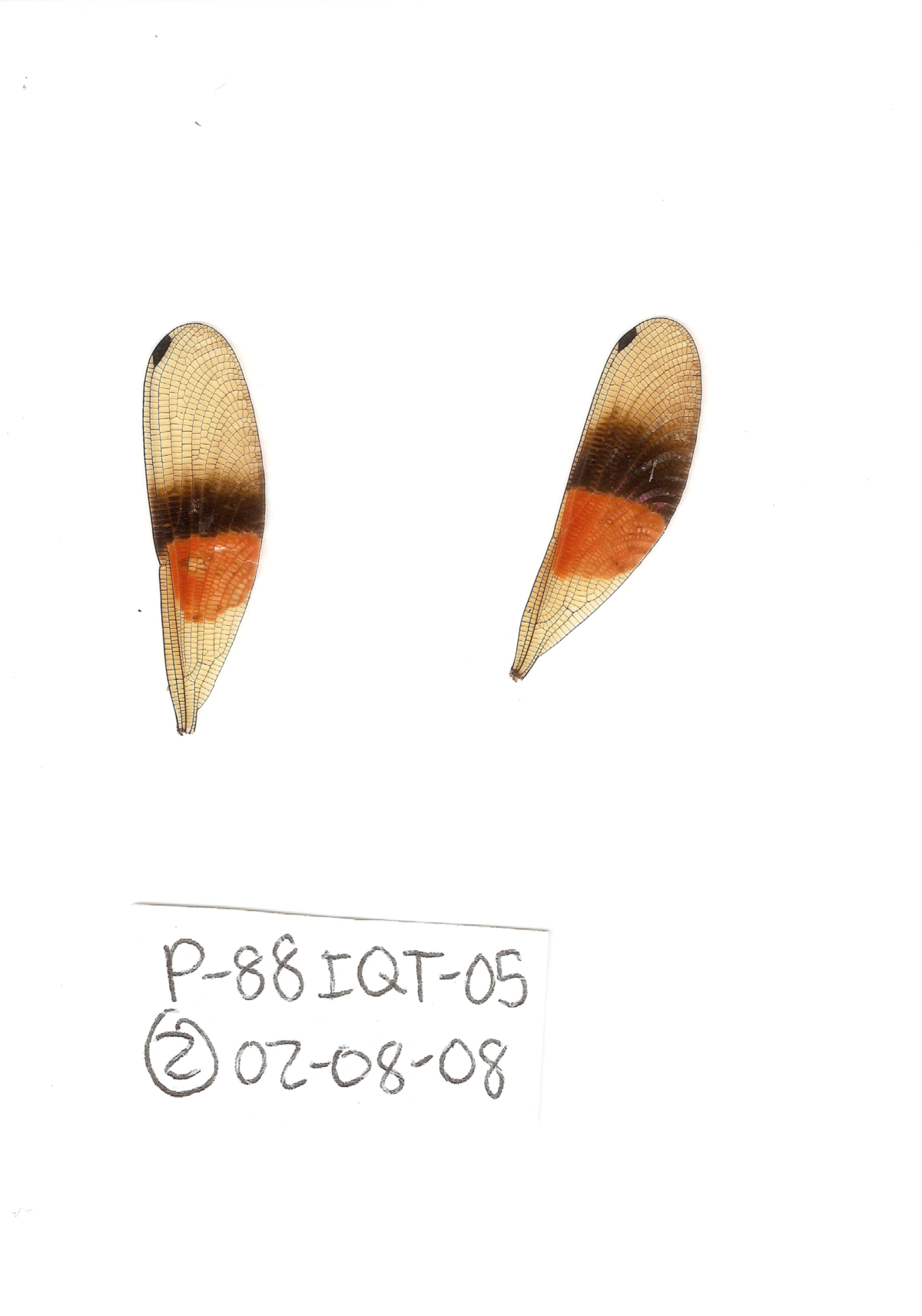

Supplement: S2 File — Compressed folder containing everything needed to run the analyses presented in this paper, including images, data, and a Mathematica notebook. (ZIP) [file pone.0125074.s002.zip › Supplementary file/images/scan0035.png]

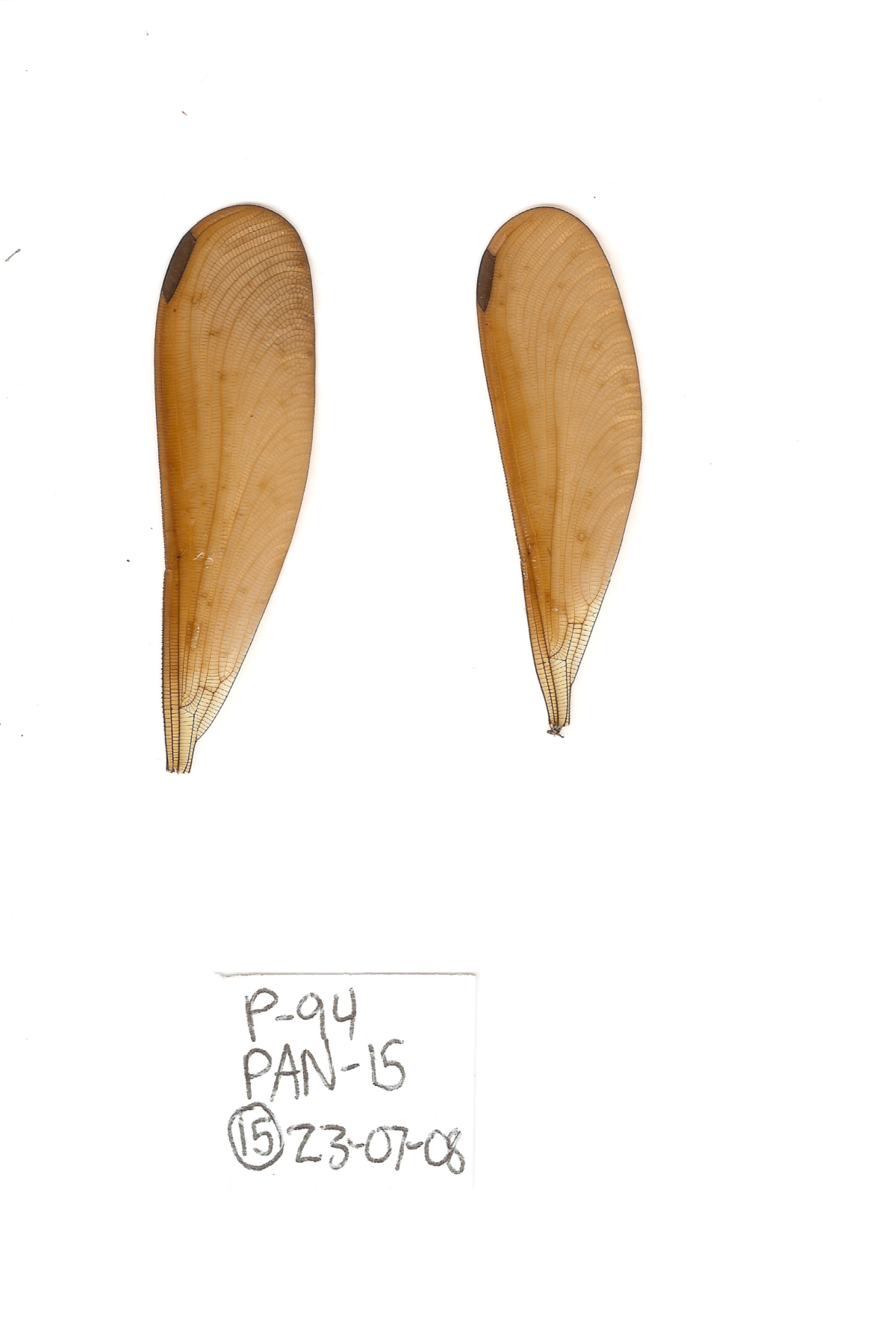

Supplement: S2 File — Compressed folder containing everything needed to run the analyses presented in this paper, including images, data, and a Mathematica notebook. (ZIP) [file pone.0125074.s002.zip › Supplementary file/images/scan0036.png]

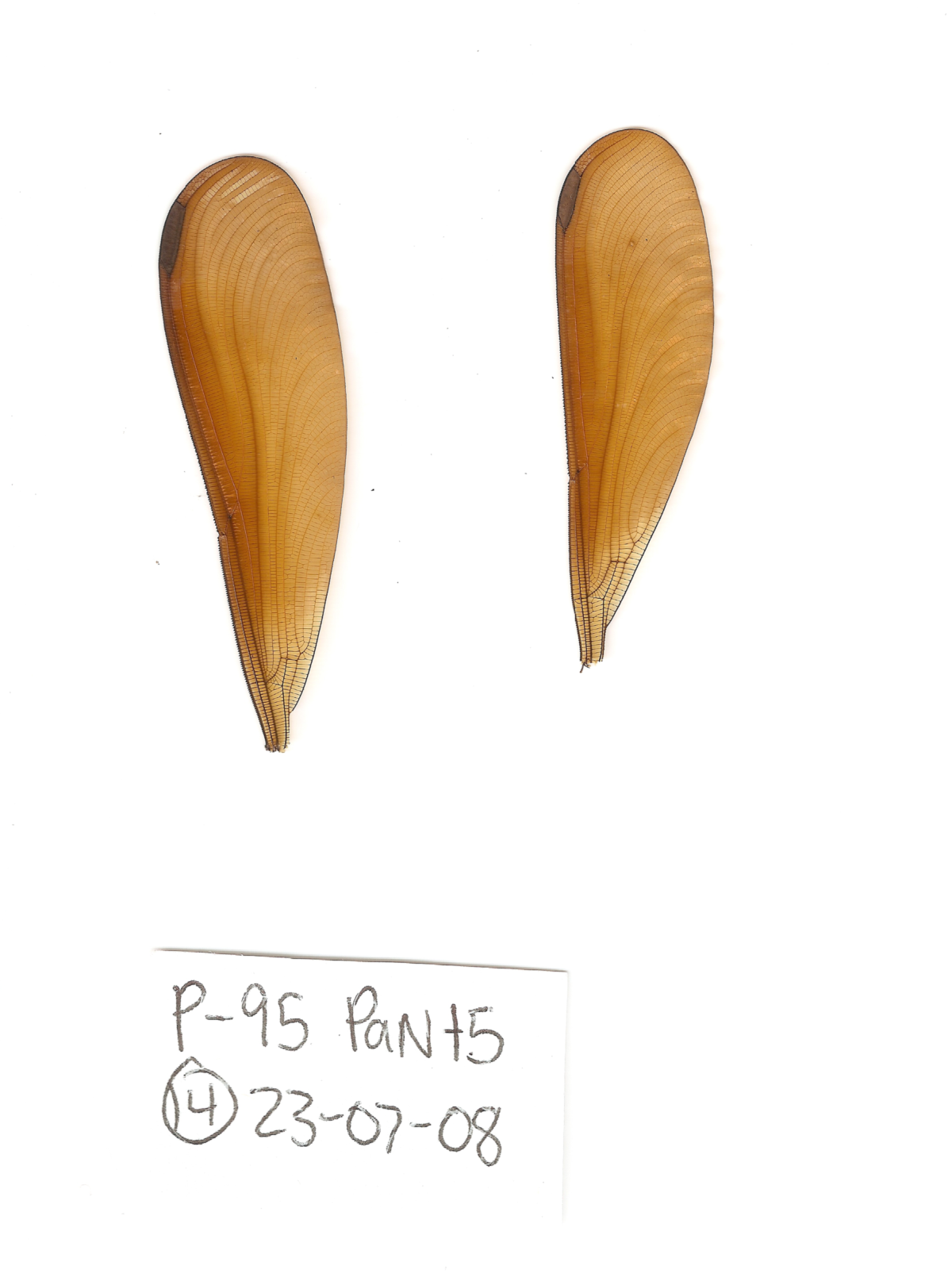

Supplement: S2 File — Compressed folder containing everything needed to run the analyses presented in this paper, including images, data, and a Mathematica notebook. (ZIP) [file pone.0125074.s002.zip › Supplementary file/images/scan0037.png]
